# Green Synthesis and Characterization of Silver Nanoparticles Using *Pleurotus eous* and Their Antioxidant and Antimicrobial Activities

**DOI:** 10.1155/ijm/4151399

**Published:** 2026-08-06

**Authors:** Mohammed Al Qutaibi, Suresh R. Kagne

**Affiliations:** ^1^ Department of Medical Microbiology, Ibb University, Ibb, Yemen, ibb-univ.net; ^2^ Department of Microbiology, Badrinarayan Barwale College, Dr. Babasaheb Ambedkar Marathwada University, Aurangabad, India, bamu.ac.in

**Keywords:** AgNPs, antimicrobials, antioxidants, biosynthesis, nanobiotechnologies, *P. eous*

## Abstract

*Pleurotus eous* (*P. eous*) contains a range of bioactive metabolites with reported antimicrobial and antioxidant potential. This work describes the green synthesis of silver nanoparticles using an aqueous extract of *P. eous* and evaluates their physicochemical features and in vitro antimicrobial activity. Silver nanoparticles were formed through fungal extract‐mediated reduction of silver nitrate, with UV–visible spectroscopy showing a surface plasmon resonance band around 420 nm. FTIR analysis indicated the involvement of fungal biomolecules in reduction and stabilization, while XRD confirmed the crystalline, face‐centered cubic structure of metallic silver. FE‐SEM imaging revealed predominantly spherical nanoparticles with an average size of 39 ± 7.6 nm. The methanolic extract of *P. eous* showed strong antioxidant activity, with DPPH radical scavenging reaching 97.39%, and contained measurable phenolic content (1.02 ± 0.07 mg GAE g^−1^ dry weight), as supported by GC–MS profiling. Antimicrobial testing demonstrated that the biosynthesized silver nanoparticles exhibited moderate inhibitory effects against *Bacillus subtilis*, *Staphylococcus aureus*, *Escherichia coli*, *Pseudomonas aeruginosa*, *Candida albicans*, *Candida tropicalis*, and *Aspergillus niger*, with inhibition zones of 11.8–16.3 mm for bacteria and 13.3–14.3 mm for fungi at 250 *μ*g. While less potent than conventional antimicrobials, these nanoparticles demonstrated measurable activity, warranting detailed toxicity and in vivo evaluation.

## 1. Introduction

The relentless escalation of antimicrobial resistance (AMR) represents one of the most pressing global health crises of our time, rendering conventional antibiotics increasingly ineffective and necessitating urgent exploration of alternative therapeutic strategies [1]. In this context, natural products, particularly those from edible fungi, have re‐emerged as promising reservoirs of bioactive compounds with diverse pharmacological properties [2]. Among these, oyster mushrooms (*Pleurotus* spp.) stand out due to their widespread cultivation, nutritional value, and documented repertoire of antioxidant, anti‐inflammatory, and antimicrobial metabolites [3–6]. *Pleurotus eous* (pink oyster mushroom) has garnered specific interest for its rich phytochemical profile, comprising phenolic acids, terpenoids, and polysaccharides with demonstrated antibacterial efficacy, particularly against clinically relevant pathogens such as *Staphylococcus aureus* [7]. Beyond their direct bioactivity, these fungal metabolites possess inherent reducing and stabilizing capacities, positioning *P. eous* as a promising candidate for the green synthesis of metallic nanoparticles, an environmentally benign alternative to conventional chemical methods that often employ hazardous reagents [8, 9].

Silver nanoparticles (AgNPs) have emerged as particularly potent antimicrobial nanomaterials due to several distinct advantages: (1) their high surface‐area‐to‐volume ratio enhances interaction with microbial membranes; (2) they exhibit broad‐spectrum activity against bacteria, fungi, and even some viruses; (3) they can penetrate biofilms that often protect resistant pathogens; and (4) they operate through multiple simultaneous mechanisms—including membrane disruption, reactive oxygen species (ROS) generation, enzyme inhibition, and interference with DNA replication—thereby reducing the likelihood of resistance development [10–13]. Oxidative stress results from excess ROS, which can lead to cellular damage. Therefore, evaluating the antioxidant activity of *P. eous* extracts is relevant to understanding the biological basis of the observed bioactivity [14]. Recent studies have further elucidated that biogenic AgNPs synthesized using natural extracts may offer advantages in biocompatibility and synergistic bioactivity compared with chemically synthesized counterparts. However, this relationship is context‐dependent and requires case‐by‐case evaluation [15–17].

The selection of *P. eous* for this study is justified by multiple factors. First, its metabolites, including quinic acid derivatives, cinnamic acid compounds, and various terpenoids, are potent reducing agents capable of converting Ag^+^ ions to Ag^0^ nanoparticles while simultaneously providing bioactive capping layers that enhance nanoparticle stability and functionality [18, 19]. Second, *Pleurotus*‐mediated AgNPs have been widely validated for their vigorous activity against multidrug‐resistant pathogens, including MRSA, and for their enhanced antibiofilm effects. Fungal synthesis may offer reproducibility advantages over some plant‐based systems owing to more consistent metabolite production in controlled mycelial cultures. Additionally, *Pleurotus* species confer antioxidant properties that help mitigate oxidative stress, and their derived AgNPs often exhibit enhanced antioxidant and antimicrobial effects [20–22].

Despite the growing body of research on fungal‐mediated nanoparticle synthesis, critical gaps remain. Most studies either focus solely on phytochemical profiling of mushroom extracts or on nanoparticle synthesis and characterization, without a direct, statistically rigorous comparison of bioactivity between the crude extract and its derived nanomaterials. Additionally, species‐specific data for *P. eous* remain limited, particularly regarding its complete metabolite profile and its performance in green nanotechnology applications.

This study, therefore, presents a novel, integrated approach that (1) provides gas chromatography (GC–MS) profiling of the methanolic extract of *P. eous*, identifying major bioactive constituents; (2) develops a green synthesis protocol for AgNPs using aqueous extracts of *P. eous*, with thorough multi‐technique characterization (UV–Vis, FTIR, XRD, FE‐SEM); and (3) conducts a direct, statistically supported comparative evaluation of the antimicrobial and antioxidant activities of both the crude extract and the biosynthesized AgNPs against a panel of clinically relevant bacterial and fungal pathogens. By integrating phytochemistry, nanotechnology, and biomedical testing in a single study, this work provides an integrated framework applicable to other medicinal fungi. It offers preliminary evidence for the potential of *P. eous* in green nanomaterial development.

Although GC–MS profiling was performed using the MeOH extract, the identified metabolite classes provide indirect biochemical evidence supporting the reducing and capping potential of *P. eous* biomolecules involved in AgNPs biosynthesis. The exact metabolites responsible for nanoparticle reduction and stabilization remain to be experimentally confirmed through future fractionation and mechanistic studies.

## 2. Materials and Methods

### 2.1. Sample Collection and Authentication

The sample of *P. eous* was collected from B.M. Mushroom and Spawn Center, Cooch Behar, West Bengal, India, 736165 (26°36 ^′^N, 89°40 ^′^E), with permission from the cultivation owner. It was identified by Sr. Prof. Arvind S. Dhabe, Head of the Department of Botany, Dr. Babasaheb Ambedkar Marathwada University (Dr. BAMU), Aurangabad, India, using macroscopic and microscopic characteristics and standard mycological keys. The dried voucher specimen of *P. eous* was deposited in the publicly accessible herbarium of the Department of Microbiology, Dr. Rafiq Zakaria Campus, affiliated with Dr. BAMU, with accession number RZC‐MIC‐HERB‐2025‐05‐B.

### 2.2. Extract Preparation

#### 2.2.1. MeOH Extract

Ten grams of the dried mushroom sample was pulverized into a fine powder. The powdered material was mixed with 100 mL of analytical‐grade MeOH. The mixture was kept at 25°C ± 2°C for 24 h, with frequent agitation. After incubation, the extract was filtered through Whatman No. 1 filter paper, and the filtrate was concentrated to dryness under reduced pressure on a rotary evaporator (Heidolph Hei‐VAP Core, Germany) until the MeOH had evaporated completely. The dried extract was stored at 4°C for further analysis. MeOH was selected for extraction for GC–MS profiling, total phenol content (TPC), and DPPH (2,2‐diphenyl‐1‐picrylhydrazyl) assays because it efficiently extracts phenolics, cinnamic/benzoic derivatives, and mid‐polarity metabolites important for antioxidant profiling. In contrast, nanoparticle synthesis used an aqueous extract, as water is a biocompatible solvent that avoids interference from organic solvents during the reduction and capping of silver ions. Thus, MeOH was used for chemical profiling while water was used for nanoparticle fabrication to maximize analytical information and ensure a green synthesis route.

#### 2.2.2. Aqueous Extract

The dried mushroom samples were washed thoroughly for 10 min with tap water, followed by a second wash with deionized water to remove any small dirt particles that could interfere with the reaction and affect the results. Then, it was cut and smashed into small pieces with a mortar and pestle. The dried samples, weighing 10 g each, were heated in 100 mL of sterilized distilled water for 30 min at 55°C ± 2°C using a magnetic stirrer. Subsequently, the resulting brown‐colored crude extract was filtered through Whatman No. 1 filter paper, and the filtrate was preserved at 4°C for subsequent use.

### 2.3. GC–MS

GC–MS analysis was carried out to establish the phytochemical profiles of the MeOH extract from *P. eous.* The study was conducted using a Shimadzu GC‐2010 Plus system equipped with an Rtx‐WAX capillary column and a GC–MS–QP2020 mass spectrometer detector. The operating conditions were as follows: helium carrier gas (1.2 mL min^−1^), an injector temperature of 250°C, and an oven programmed from 60°C to 280°C at 5°C min^−1^. The mass spectrometer had a scan range of m/z 40–550 amu, and was in electron ionization mode at 70 eV. Phytochemicals were identified by comparing their retention times and mass spectra with those of the NIST Mass Spectral Library (version 14). Only those compounds whose fragmentation pattern was verified and whose similarity score was greater than 70% were considered. The relative percentages were calculated from peak area ratios of the total ion chromatogram (TIC).

### 2.4. Biosynthesis of AgNPs

#### 2.4.1. Silver Nitrate Solution (AgNO₃)

An AgNO₃ solution was prepared by dissolving ~0.5096 g (MW 169.87 g mol^−1^) of AgNO₃ of analytical grade in 1000 mL of deionized water. The freshly prepared (3 mM) AgNO₃ solution was stored in an amber glass flask, properly labeled, to ensure safe storage conditions.

#### 2.4.2. Biogenic AgNPs With *P. eous* Extract

The green synthesis of AgNPs involved the use of the aqueous extract of *P. eous* as a reducing and stabilizing agent. Eighty milliliters of AgNO₃ was mixed with 20 mL of the extract to get a 4:1 AgNO₃‐to‐extract ratio. The reaction mixture was incubated in the dark at room temperature for 24 h with frequent visual inspection for color change. The brown color, which is an indication of the AgNPs formation, appeared after the reaction had been incubated. After that, the mixture was centrifuged at 20,000 RPM for 10 min, and the supernatant was removed after each cycle. The pellet was washed and centrifuged three times with deionized water to ensure it was free from contamination. The purified nanoparticles were dried for 24 h at 49°C ± 2°C in a hot air oven and were then stored in dark refrigeration conditions for further characterization.

#### 2.4.3. Characterization of AgNPs

Several analytical techniques were used to characterize the biosynthesized AgNPs [23]. UV–Vis spectroscopy was employed to confirm nanoparticle formation through surface plasmon resonance (SPR) analysis. XRD analysis was applied to confirm crystallinity and phase structure. Crystallite size was estimated from the (111) diffraction peak using the Scherrer equation (D = K*λ*/*β*cos*θ*), with shape factor K = 0.9, Cu K*α* radiation (*λ* = 1.5406 Å), and *β* = 0.0157. FE‐SEM analysis was used to evaluate nanoparticle morphology and size distribution. Particle size was determined by measuring (*n* = 82) individual particles from FE‐SEM images using ImageJ. FTIR spectroscopy was used to identify functional groups associated with biomolecule‐mediated reduction and stabilization of AgNPs.

### 2.5. DPPH

The antioxidant potential of *P. eous* was evaluated using the DPPH radical scavenging assay [24]. A 0.1 mM DPPH solution was prepared by dissolving 3.94 mg of DPPH in 100 mL of high‐purity MeOH in an amber flask. A reagent blank consisting of 1 mL of MeOH and 1 mL of DPPH solution was used to zero the spectrophotometer. Ascorbic acid (0.1–0.7 mg mL^−1^) served as the positive control standard. One milliliter of *P. eous* extract at concentrations of 0.1, 0.3, 0.5, and 0.7 mg mL^−1^ was mixed with 1 mL of DPPH solution. After 30 min of dark incubation, the mixtures were measured with a UV–Vis spectrophotometer (Shimadzu, UV–1780, Japan) at 517 nm. The formula for calculating the percentage of radical scavenging (inhibition) is [(*A*_0_ − *A*_1_)/*A*_0_] × 100, where: *A*_0_ is absorbance of control and *A*_1_ is absorbance of sample. To exclude the extract’s color, a sample blank (extract without DPPH) was measured and its absorbance subtracted from *A*_1_ before calculating % inhibition.

### 2.6. TPC

The TPC of the *P. eous* MeOH extract was determined using the Folin–Ciocalteu (FC) colorimetric method [25], with gallic acid as the standard phenolic compound. Briefly, 0.5 mL of appropriately twofold‐diluted mushroom extract (to achieve an absorbance within the validated linear range of the calibration curve) was mixed with 2.5 mL of 10% (v/v) FC reagent and allowed to stand at room temperature for 5 min. Then, 2 mL of 7.5% (w/v) sodium carbonate (Na₂CO₃) was added, and the mixture was incubated in the dark at 25°C ± 2°C for 30 min. Absorbance was measured at 765 nm against a blank using a UV–vis spectrophotometer. A gallic acid calibration curve (0–100 *μ*g mL^−1^) was prepared under identical conditions. All measurements were performed in triplicate. The regression equation obtained was y = 0.0014293x + 0.2334 (*R* ^2^ = 0.9969), where y is absorbance and x is gallic acid concentration (*μ*g mL^−1^). The total phenolic content was expressed as milligrams of gallic acid equivalent per gram of dry weight (mg GAE g^−1^ DW).

### 2.7. Antimicrobial Activity

#### 2.7.1. Microbial Strain Collection and Preparation

Seven different microbial strains were collected from the Government Institute of Forensic Science in Aurangabad, India, and subsequently stored in the microbiology laboratory at the Dr. Rafiq Zakaria Campus. The strains included four bacterial isolates: *Bacillus subtilis* (NCTC 10400), *Staphylococcus aureus* (NCTC 10788), *Escherichia coli* (ATCC 8793), and *Pseudomonas aeruginosa* (NCIM 5029); and three fungal isolates: *Candida albicans* (ATCC 10231), *Candida tropicalis* (NCIM 3110), and *Aspergillus niger* (ATCC 9642). Microbial cultures were standardized to a 0.5 McFarland turbidity by spectrophotometry at 600 nm before antimicrobial testing, ensuring consistent cell density.

#### 2.7.2. Agar Well Diffusion Assay

The antimicrobial activity of the MeOH extract and *P. eous*‐mediated AgNPs was investigated with an agar well diffusion assay with modification [26].

2.7.2.1 MeOH Extract. The MeOH extract was made at concentrations of 100, 150, and 200 mg mL^−1^ with DMSO. The bacterial suspension was spread onto Mueller–Hinton agar plates. Using a sterile cork borer, 8 mm wells were created, and 100 *μ*L of the extract (equal to 10 × 10^3^, 15 × 10^3^, and 20 × 10^3^ *μ*g) was added to each well. All the bacterial samples were kept at 37°C for 24 h. CIP (1 *μ*g) and S (10 *μ*g) were used as a positive control, while DMSO was employed as a negative control.

2.7.2.2 *P. eous*‐Mediated AgNPs. The microbial standardized inoculum (100 *μ*L) was spread onto Mueller–Hinton agar (bacteria) or Sabouraud Dextrose agar (fungi). On the same agar plates, different volumes of 2.5 mg mL^−1^ AgNPs (20–100 *μ*L) were used to create wells, resulting in concentrations ranging from 50 to 250 *μ*g mL^−1^. CIP (1 *μ*g) and S (10 *μ*g) were used as positive controls for bacteria, while fluconazole (4 *μ*g) served as the positive control for fungi. AgNO₃ and DMSO solutions were used as controls to distinguish the antimicrobial effect of Ag+ and the extract from that of AgNPs. Bacterial plates were kept at 37°C for 24 h, and fungal plates were kept at 30°C for 48 h. A uniform agar plate thickness was maintained throughout the assay, and the plates were left at room temperature for 15 min after sample loading to allow adequate diffusion of the extracts and nanoparticles before incubation.

### 2.8. Data Analysis

All experimental data were expressed as mean ± standard deviation (SD) for triplicate measurements (*n* = 3). Statistical analyses were performed using the individual replicate measurements. For antioxidant data, a two‐way ANOVA was applied with sample type and concentration as factors, including their interaction, followed by Tukey’s HSD post hoc test. For antimicrobial data, one‐way ANOVA was used to compare treatment groups at the highest tested dose, followed by Tukey’s HSD post hoc test at *p* < 0.05.

## 3. Results

### 3.1. GC–MS

The GC–MS analysis of *P. eous* MeOH extract identified several compounds with established or potential antimicrobial properties, each contributing to the extract’s overall bioactivity (Table 1 and Table S1). Notably, carvacrol, a natural monoterpenoid, is well documented for its broad‐spectrum antibacterial and antifungal effects, primarily through disruption of microbial membranes, and is widely used as a food additive due to its safety and efficacy [38–40]. In addition, the detection of 3‐nonynoic acid, structurally related to nonanoic acid, is significant since nonanoic acid has indicated potent antifungal activity, particularly against *C. albicans* and dermatophytes such as *Trichophyton mentagrophytes*, and is recognized for its ability to inhibit spore germination and mycelial growth in pathogenic fungi [29, 30]. Quinic acid, another compound present in the extract, is known for its astringent properties and has been shown to possess broad‐spectrum antimicrobial activity, especially against Gram‐positive and Gram‐negative bacteria. However, its antifungal effect is less pronounced [31, 41–43]. The antimicrobial action of quinic acid and its derivatives is attributed to their ability to disrupt bacterial membranes and inhibit biofilm formation [41–43]. Benzoic acid derivatives, such as 2‐hydroxy‐4‐methoxy‐3,5,6‐trimethylbenzoic acid, are frequently discussed for their anti‐inflammatory, hepatoprotective, and antimicrobial activities. These compounds have indicated efficacy against both bacterial and fungal pathogens, with some derivatives showing selective activity and low toxicity in mammalian cells [31, 44–46]. Similarly, cinnamic acid derivatives (e.g., 3,4‐dimethoxycinnamic acid) are well established for their antimicrobial potential, exhibiting activity against a wide range of bacteria and fungi. They are considered promising candidates for drug development due to their low toxicity and structural versatility [47–50]. The presence of thiosulfuric acid esters and eseroline derivatives in the extract may contribute to both toxicity and antimicrobial activity, as sulfur‐containing compounds are known for their bioactive properties. However, their safety profiles require further investigation [51].

**Table 1:** Compounds identified by GC–MS in *P. eous* extract (compound name, formula, bioactivity, and chemical structure).

Compound name	Formula	Biological activity	Chemical structure	Refs.
Carvacrol	C_10_H1_40_	Antibacterial, antifungal, anticancer, antiproliferative, and antioxidant	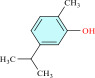	[27, 28]
3‐Nonynoic acid	C_9_H_14_O_2_	Potential antifungal, herbicidal, and anti‐inflammatory	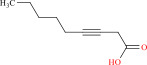	[29, 30]
Quinic acid	C_7_H_12_O_6_	Antioxidant, anticancer, anti‐Inflammatory, antimicrobial	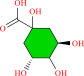	[31–33]
3,4‐Dimethoxy cinnamic acid	C_11_H_12_O_4_	Antioxidant, antimicrobial potential antioxidant; anticancer effects	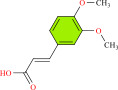	[34, 35]
2‐Hydroxy‐4‐methoxy‐3,5,6‐trimethylbenzoic acid	C_11_H_14_O_4_	Anti‐inflammatory, hepatoprotective, and antimicrobial defense mechanisms	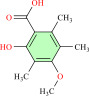	[36]
Eseroline, N, N‐diethylcarbamate(ester)	C_18_H_27_N_3_O_2_	Opioid activity; possible antimicrobial properties	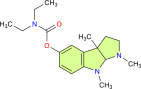	[37]

### 3.2. AgNPs Characterization

**Figure 1:**
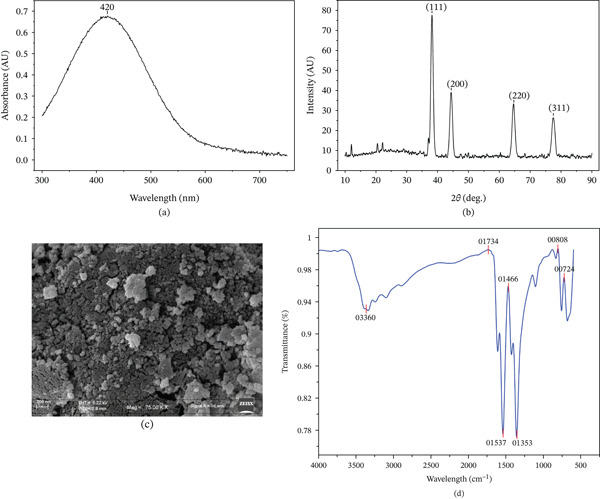
Characterization of AgNPs synthesized with *P. eous*. (a) UV–Visible absorption spectrum of AgNPs produced with *P. eous*. The intense SPR peak detected at about 420 nm. (b) X_−_ray diffraction (XRD) pattern of synthesized nanoparticles. The diffractogram indicates a crystalline nature of the sample with four main peaks at 2*θ* values 38.30°, 44.18°, 65.1°, and 76.9°. (c) The agglomeration and distribution of AgNPs with FE‐SEM. Scale bar 200 nm, magnification 75,000×. (d) The key peaks of absorbance in the FTIR analysis.

The biosynthesized AgNPs exhibited a distinct SPR peak at 420 nm in the UV–vis spectrum, confirming nanoparticle formation (Figure 1a). XRD analysis showed prominent diffraction peaks at 2*θ* values of 38.30°, 44.18°, 65.10°, and 76.90°, which match the (111), (200), (220), and (311) planes of face‐centered cubic silver, confirming the crystalline nature of the nanoparticles. Crystallite size was calculated with the Scherrer equation to be 9.4 nm (Figure 1b). FE‐SEM imaging demonstrated that the nanoparticles were predominantly spherical to quasi‐spherical, with a degree of aggregation and a mean particle diameter of 39 ± 7.6 nm (Figure 1c). FTIR analysis revealed characteristic absorption bands corresponding to O▬H (~3360 cm^−1^), N▬H (~1537 cm^−1^), and C〓O (~1734 cm^−1^) functional groups, indicating the involvement of phenolics and proteinaceous biomolecules from the *P. eous* extract in reducing and stabilizing the nanoparticles (Figure 1d).

### 3.3. DPPH

**Figure 2:**
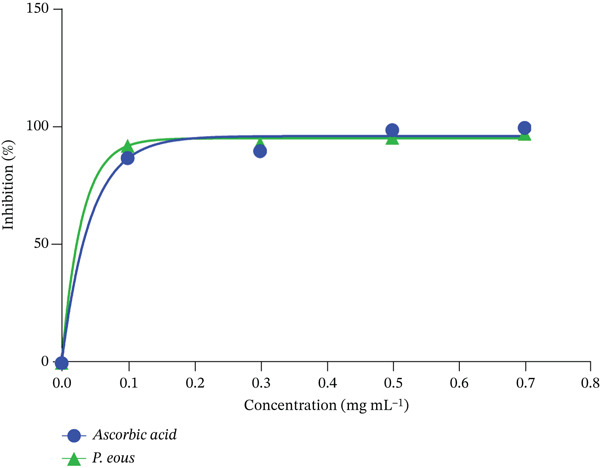
Radical scavenging activity of mushroom extracts from *P. eous*, compared with ascorbic acid (positive control), as determined by the DPPH assay. The results are expressed as percentage inhibition of DPPH free radicals at various concentrations of the extract and standard. The dose‐dependent increase in inhibition indicates strong antioxidant potential of *P. eous*.

**Figure 3:**
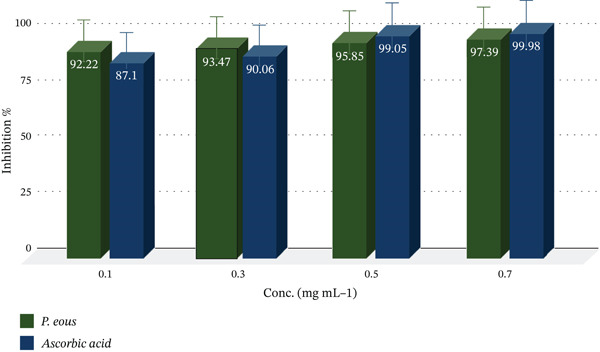
Percentage inhibition exhibited by *P. eous* extract compared with ascorbic acid (positive control) at different concentrations (0.1–0.7 mg mL^−1^). Bars represent mean inhibition values, and error bars indicate standard deviation, demonstrating a concentration‐dependent inhibitory effect for both treatments.

The control DPPH solution exhibited an absorbance of 0.845 ± 0.002 at 517 nm. The MeOH extract of *P. eous* exhibited strong DPPH radical‐scavenging activity, with inhibition values of 92.22%, 93.47%, 95.85%, and 97.39% at 0.1, 0.3, 0.5, and 0.7 mg mL^−1^, respectively (Table S2). Ascorbic acid displayed 87.10%, 90.05%, 99.05%, and 99.89% inhibition at the same concentrations. Both samples demonstrated an apparent dose‐dependent increase in antioxidant capacity. Notably, *P. eous* exhibited slightly higher activity than ascorbic acid at the lower concentrations (0.1–0.3 mg mL^−1^), while ascorbic acid showed superior inhibition at higher concentrations (0.5–0.7 mg mL^−1^). Overall, both the extract and the standard exhibited strong antioxidant potential, with all inhibition values exceeding 85%, consistent with the trend illustrated in Figure 2. The concentration‐dependent antioxidant activity of *P. eous* in comparison with ascorbic acid is illustrated in Figure 3.

### 3.4. TPC

The MeOH extract of *P. eous* exhibited an absorbance of 0.306 ± 0.005 at 765 nm, which fell within the linear range of the calibration curve. Using the regression equation (y = 0.0014293x + 0.2334, *R* ^2^ = 0.9969), the equivalent gallic acid concentration was 50.82 *μ*g GAE mL^−1^. After correcting for the two‐fold dilution, the final concentration was 101.64 *μ*g GAE mL^−1^. Given that 10 g of dried mushroom powder was extracted with 100 mL of MeOH (0.1 g mL^−1^), the total phenolic content was 1.02 ± 0.07 mg GAE g^−1^ DW. This value falls within the range reported for *Pleurotus* species (0.8–10.0 mg GAE g^−1^ DW) [52–54], consistent with the typical MeOH extraction of edible mushrooms. Overall, the results indicate that *P. eous* has a moderate level of total phenolics compared with other *Pleurotus* species. It should be noted that the Folin–Ciocalteu assay measures overall reducing capacity and may respond to other reducing substances; therefore, the reported values represent gallic acid equivalents rather than absolute phenolic concentrations.

### 3.5. Antibacterial Activity

#### 3.5.1. MeOH Extract

At lower concentrations (10 − 15 × 10^3^ *μ* *g*), activity was minimal or absent for *S. aureus* and *E. coli*; however, *B. subtilis* and *P. aeruginosa* showed weak but measurable inhibition (11–12 mm) across all tested concentrations. Only at the highest dose (20 × 10^3^ *μ* *g*) did all four strains exhibit quantifiable inhibition zones. At 20 × 10^3^ *μ* *g*, the extract yielded zones of 13.0 ± 2.0 mm for *B. subtilis* and 11.3 ± 0.5 mm for *S. aureus*, 16.5 ± 0.5 mm for *E. coli*, and 13.8 ± 0.7 mm for *P. aeruginosa* (Table 2).

**Table 2:** Zones of inhibition (mm) for MeOH extract of *P. eous* against bacterial strains. Each value is the mean of three replicates (± SD); “R” indicates resistance.

Bacteria	*P. eous*			NC	PC	
	10 × 10^3^ *μ* *g*	15 × 10^3^ *μ* *g*	20 × 10^3^ *μ* *g*	DMSO	CIP (1 *μ*g)	S (10 *μ*g)
*B. subtilis*	11.5 ± 0.5	12.6 ± 2.0	13 ± 2.0	0	29.8 ± 0.8	22 ± 2.0
*S. aureus*	R	R	11.3 ± 0.5	0	28.8 ± 0.8	16.7 ± 1.5
*E. coli*	R	R	16.5 ± 0.5	0	26 ± 0.5	19.3 ± 1.2
*P. aeruginosa*	11 ± 2.0	12.3 ± 1.5	13.8 ± 0.7	0	41.5 ± 0.5	18.3 ± 1.52

*Note:* Antibiotic disc controls were included on all assay plates, and the values shown represent a shared reference measurement set.

Abbreviations: CIP: ciprofloxacin (antibiotic); S: streptomycin (antibiotic).

#### 3.5.2. *P. eous*‐Mediated AgNPs

By contrast, AgNPs produced larger inhibition zones than the crude extract (Table 3). At 250 *μ*g, AgNPs produced mean inhibition zones of 16.0 ± 1.0 mm (*B. subtilis*), 15.3 ± 0.6 mm (*S. aureus*), and 16.3 ± 0.6 mm (*P. aeruginosa*). AgNPs showed less activity against *E. coli* (11.8 ± 0.3 mm) than the MeOH extract. The positive controls (CIP 1 *μ*g and S 10 *μ*g) yielded larger zones (29.8 and 22.0 mm for *B. subtilis*, 28.8 and 16.7 mm for *S. aureus*, 26 and 19.3 mm for *E. coli*, and 41.5 and 18 mm for *P. aeruginosa* [CIP and S, respectively]), while the negative controls had no effect.

**Table 3:** Antibacterial activity of *P. eous*–AgNPs (agar well diffusion). Mean zone of inhibition (mm) ± SD (*n* = 3) for various AgNPs doses and antibiotic controls, “R” indicates resistance.

Microorganism	AgNPs		CIP (1 *μ*g)	S (10 *μ*g)	DMSO	AgNO₃ (*μ*L)		
	Conc. (*μ*g)	*M* *e* *a* *n* ± *S* *D*(mm)	*M* *e* *a* *n* ± *S* *D*(mm)	*M* *e* *a* *n* ± *S* *D*(mm)	*M* *e* *a* *n* ± *S* *D*(mm)	10	20	25
*B. subtilis*	250	16 ± 1.0	29.8 ± 0.8	22 ± 2.0	0	R	R	R
	200	15.8 ± 0.3						
	150	15.6 ± 0.6						
	100	15.5 ± 0.5						
	50	14.3 ± 0.6						
*S. aureus*	250	15.3 ± 0.6	28.8 ± 0.8	16.7 ± 1.5	0	R	R	R
	200	14.3 ± 0.6						
	150	14.1 ± 0.3						
	100	13.3 ± 0.6						
	50	11.3 ± 0.6						
*E. coli*	250	11.8 ± 0.3	26 ± 0.5	19.3 ± 1.2	0	R	R	R
	200	11.5 ± 0.5						
	150	11.3 ± 0.6						
	100	R						
	50	R						
*P. aeruginosa*	250	16.3 ± 0.6	41.5 ± 0.5	18.3 ± 1.52	0	R	11	12
	200	15.5 ± 0.5						
	150	14.3 ± 0.6						
	100	13.3 ± 0.6						
	50	12.5 ± 0.5						

*Note:* Antibiotic disc controls were included on all assay plates, and the values shown represent a shared reference measurement set.

Abbreviations: CIP: ciprofloxacin (antibiotic); S: streptomycin (antibiotic).

### 3.6. Antifungal Activity

The AgNPs, at a concentration of 250 *μ*g, inhibited the growth of *C. albicans* (13.3 ± 0.6 mm), *C. tropicalis* (14.3 ± 0.6 mm), and *A. niger* (14.3 ± 0.6 mm). Fluconazole produced larger zones (21.2 ± 0.8 mm for *C. albicans*, 21 ± 0.9 mm for *C. tropicalis*, 17.2 ± 0.8 mm for *A. niger*) (Table 4).

**Table 4:** Zones of inhibition (mm) for *P. eous*–AgNPs against fungal strains. Each value is the mean of three replicates (± SD); “R” indicates resistance.

Microorganism	Conc. (*μ*g)	*M* *e* *a* *n* ± *S* *D*(mm)	DMSO	AgNO_3_ (*μ*L)	FLC (4 *μ*g)
*C. albicans*	250	13.3 ± 0.6	0	R	21.2 ± 0.8
	200	12.5 ± 0.5			
	150	12.3 ± 0.6			
	100	11.3 ± 0.6			
	50	R			
*C. tropicalis*	250	14.3 ± 0.6	0	R	21 ± 0.9
	200	13.5 ± 0.5			
	150	13.3 ± 0.6			
	100	12.3 ± 0.6			
	50	11.3 ± 0.6			
*A. niger*	250	14.3 ± 0.6	0	R	17.2 ± 0.8
	200	13.5 ± 0.9			
	150	13.3 ± 0.6			
	100	13.3 ± 0.8			
	50	13.2 ± 0.3			

Abbreviation: FLC: fluconazole.

**Figure 4:**
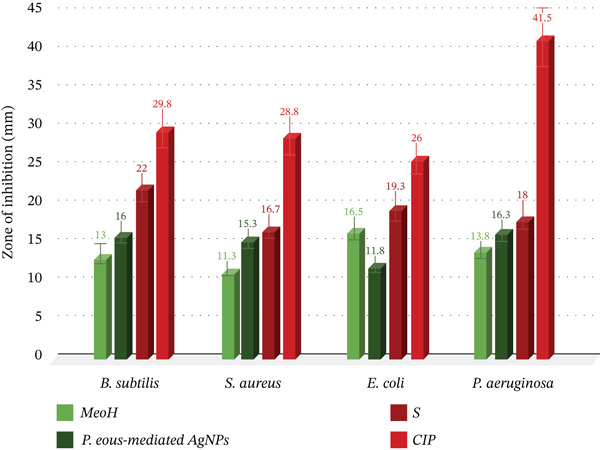
Antibacterial activity expressed as zones of inhibition (mm) for MeOH extract, *P. eous*‐mediated AgNPs, streptomycin (S), and ciprofloxacin (CIP) against *B. subtilis*, *S. aureus*, *E. coli*, and *P. aeruginosa*. Values represent mean inhibition diameters, illustrating the enhanced antibacterial efficacy of AgNPs relative to the crude extract and comparison with standard antibiotics.

**Figure 5:**
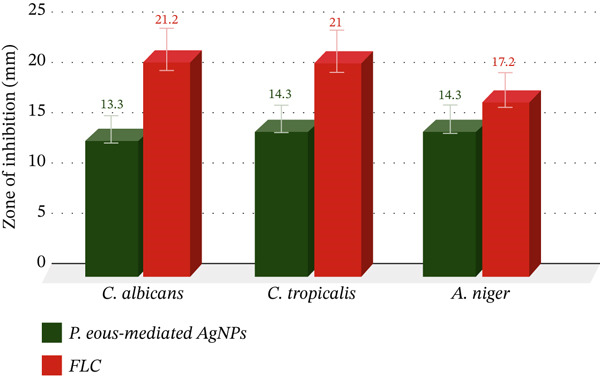
Antifungal activity expressed as zones of inhibition (mm) of *P. eous*‐mediated AgNPs compared with fluconazole (FLC) against *C. albicans*, *C. tropicalis*, and *A. niger*. Bars represent mean inhibition values, and error bars indicate standard deviation, highlighting the comparative antifungal efficacy of biosynthesized AgNPs relative to the standard antifungal agent.

The antibacterial and antifungal efficacy of the MeOH extract, biosynthesized AgNPs, and standard antibiotics against the tested bacterial and fungal strains is presented in Table S3 and in Figures 4 and 5.

### 3.7. Statistical Comparison of Treatments

The antioxidant activity of both the *P. eous* MeOH extract and ascorbic acid increased progressively with concentration (0.1–0.7 mg mL^−1^). At lower concentrations (0.1–0.3 mg mL^−1^), the extract showed slightly higher radical‐scavenging activity than ascorbic acid (*p* < 0.01), whereas ascorbic acid exhibited significantly greater inhibition at higher concentrations (0.5–0.7 mg mL^−1^, *p* < 0.01). Overall, both treatments demonstrated strong concentration‐dependent antioxidant activity, with highly significant effects of sample type (F (1, 16) = 43.64, *p* < 0.001), concentration (F (3, 16) = 1194.90, *p* < 0.001), and their interaction (F (3, 16) = 304.38, *p* < 0.001).

For antibacterial activity, standard antibiotics (CIP and S) produced the largest inhibition zones (25–30 mm), significantly exceeding those of both the *P. eous* extract and biosynthesized AgNPs (*p* < 0.05). The MeOH extract exhibited measurable antibacterial activity mainly at the highest tested concentration (20 × 10^3^ *μ*g). In contrast, lower concentrations showed limited or no detectable inhibition against some strains, particularly *S. aureus* and *E. coli*. In contrast, the biosynthesized AgNPs demonstrated significantly greater antibacterial activity than the crude extract against *B. subtilis*, *S. aureus*, and *P. aeruginosa* (*p* < 0.05), producing inhibition zones ranging from 11.8 to 16.3 mm. However, the MeOH extract showed greater inhibition against *E. coli* than the AgNPs. Overall differences among antibacterial treatments were highly significant (F(4, 10) = 176.98, *p* < 0.001), indicating that nanoparticle biosynthesis enhanced the antimicrobial efficacy of *P. eous* against most of the tested bacterial strains. For fungal pathogens, the biosynthesized AgNPs exhibited measurable antifungal activity against *C. albicans*, *C. tropicalis*, and *A. niger*, with inhibition zones ranging from 11.3 to 14.3 mm. Although these effects were significantly lower than those of fluconazole (17.2–21.2 mm, *p* < 0.01), the AgNPs consistently exhibited greater antifungal activity than the negative control (*p* < 0.05). Overall, the results confirm the broad‐spectrum but moderate antimicrobial potential of the biosynthesized AgNPs.

## 4. Discussion

### 4.1. Antioxidant Activity

The DPPH assay demonstrated that *P. eous* extract exhibited 92.22% radical‐scavenging inhibition at 0.1 mg mL^−1^ and increased to 97.39% at 0.7 mg mL^−1^. Ascorbic acid showed greater inhibition at higher concentrations. These results align with reported values for other *Pleurotus* species (e.g., 63%–97% inhibition, depending on the extraction method). *P. eous* MeOH, ethyl acetate, and hot water extracts showed inhibition between 4.3% and 87.9% at 2–50 mg mL^−1^ [55]. MeOH, chloroform, and hot water extracts of *P. ostreatus* and related species showed high inhibition (up to 97.9%), indicating potent antioxidant potential and supporting their value as natural sources of bioactive compounds for oxidative stress management [56–58]. All tested concentrations produced inhibition exceeding 85%, indicating that the IC_50_ is below the minimum tested concentration (0.1 mg mL^−1^). To accurately determine the IC_50_, future studies should include lower extract concentrations. The phenol content of the extract (1.02 mg GAE g^−1^) and the antioxidant activity measured by the DPPH assay are consistent with those of other *Pleurotus* species [59]. The antioxidant capacity may correlate with antimicrobial activity, as phenolic compounds are known to exhibit both properties; however, mechanistic studies are needed to determine whether the observed antimicrobial effects are mediated by the same compounds responsible for radical scavenging.

### 4.2. Phytochemical Profile and AgNPs Formation

The results indicate that *P. eous* contains compounds with antimicrobial and antioxidant activities and can mediate the synthesis of AgNPs under laboratory conditions. GC–MS of the MeOH extract identified numerous phenolics and terpenoids known for their bioactivity. For example, compounds such as quinic acid, 2‐hydroxy‐4‐methoxy‐3,5,6‐trimethylbenzoic acid, and eseroline derivative have documented antimicrobial and antioxidant effects [31–33, 37, 60–62]. Collectively, these findings, supported by the literature on *P. eous* and related natural products, indicate that the extract contains a diverse array of biologically active compounds with attractive antimicrobial properties.

### 4.3. Characterization of AgNPs

The current UV–vis, XRD, and SEM data suggest the typical formation of AgNPs, indicating that *P. eous* biomolecules potentially reduced and capped the AgNPs. This is in line with other fungal‐mediated syntheses. For instance, numerous studies have demonstrated that oyster mushrooms produce spherical AgNPs with similar optical signatures and diameters of less than 100 nm [63–65]. The crystallite size derived from the Scherrer equation (9.4 nm) was considerably smaller than the mean FE‐SEM diameter (39 nm), consistent with polycrystalline particles comprising multiple coherent diffraction domains, a pattern commonly reported for biogenic AgNPs [66]. The FE‐SEM images also show particle aggregation, which may contribute to the larger apparent diameters. The functional groups identified by FTIR include phenolic and proteinaceous moieties, both of which reduce Ag^+^ and stabilize the particles [67]. Absorption bands corresponding to O▬H stretching (~3360 cm^−1^), N▬H (~1537 cm^−1^), and ester/carboxylic C〓O stretching (~1734 cm^−1^) were observed, indicating the involvement of phenolic hydroxyl groups and proteinaceous biomolecules. These functional groups are consistent with roles in reduction and capping, but mechanistic confirmation (e.g., biochemical assays, proteomics, membrane integrity assays) is required.

### 4.4. Antimicrobial Activity of *P. eous* and AgNPs

The antibacterial tests showed that the *P. eous* MeOH extract exhibited very weak activity compared with AgNPs and standard antibiotics, particularly at high concentrations, consistent with findings in other oyster mushrooms. A study in 2024 revealed that *P. eous* extract could exert the maximum inhibitory effect on *S. aureus*, and SEM imaging revealed morphological evidence consistent with membrane damage in bacteria exposed to the extract [7]. In the current study, the MeOH extract showed weak‐to‐moderate activity predominantly at the highest tested concentration (20 × 10^3^ *μ*g). At this concentration, inhibition zones ranged from 11.3 (*S. aureus*) to 16.5 mm (*E. coli*). At lower concentrations (10 × 10^3^–15 × 10^3^ *μ*g), activity was minimal or indistinguishable from that of the controls. These zones were smaller than those of standard antibiotics. The discrepancy between the strong findings of Dash et al. and the relatively small inhibition zones observed in the present study may be due to differences in extraction methods, assay conditions, or extract composition [7].

Antibacterial testing of AgNPs demonstrated broad‐spectrum efficacy, with inhibition zones of up to ~16 mm at 250 *μ*g. This supports reports that AgNPs have broad‐spectrum action [22, 68–70]. The AgNPs inhibition zones against *B. subtilis* and *P. aeruginosa* (~16 mm) are comparable with those reported in similar studies. For example, other mushroom‐based AgNPs frequently result in inhibition zones of sizes ranging from 12 to 20 mm against *B. subtilis* and *P. aeruginosa* at comparable doses [71, 72]. *E. coli* showed the lowest susceptibility to AgNPs (11.8 mm), which may reflect strain‐specific resistance mechanisms rather than a general Gram‐negative pattern, since *P. aeruginosa*, also Gram‐negative, exhibited the highest inhibition zone (16.3 mm) of all tested strains. The differential susceptibility among Gram‐negative species warrants further investigation [73]. While AgNPs enhanced activity against most strains, the high‐concentration crude extract (20 × 10^3^ *μ*g) showed greater inhibition only against *E. coli* (16.5 ± 0.5 mm vs. 11.8 ± 0.3 mm for AgNPs), suggesting that the crude extract’s phytochemical profile is more effective against this strain.

These findings partially support the hypothesis, as *P. eous*‐mediated AgNPs showed enhanced antimicrobial activity compared with the crude extract. The *P. eous* extract, which contains moderate phenolic content, possesses the capacity of a natural antioxidant. When used to fabricate AgNPs, it confers measurable broad‐spectrum antibacterial and antifungal activity upon the biosynthesized AgNPs. Although AgNPs were less potent than standard drugs in these assays, their moderate broad‐spectrum activity and green synthesis route warrant further mechanistic and safety studies. Future work should include in vivo efficacy and toxicity studies, as well as the elucidation of the nanosilver‐killing mechanisms in target microbes, as suggested by others in the field.

Nevertheless, the statistical results indicate that all antibiotic treatments are significantly stronger (*p* < 0.05) than AgNPs; importantly, AgNPs are significantly more active than the extract and AgNO₃ alone (*p* < 0.05). These findings indicate that nanoformulation significantly enhanced the antimicrobial efficacy of *P. eous*‐derived biomolecules compared with the crude extract alone [74]. The antifungal results follow a similar pattern*; P. eous*‐mediated AgNPs inhibited *Candida* spp. and *A. niger* (13–14 mm), albeit less than fluconazole (17–21 mm). This aligns with studies showing that AgNPs can damage fungal cells by altering cell membranes and generating oxidative stress, producing moderate inhibition zones on par with or sometimes exceeding those of plant extracts [64, 75, 76]. The current findings reinforce the conclusion that *Pleurotus*‐derived AgNPs exhibit genuine antifungal activity, although their potency is somewhat lower than against bacteria. The statistical tests (*p* < 0.001) confirm that all treatment differences are highly significant, underscoring the reliability of these trends.

### 4.5. Proposed Mechanisms of AgNPs Biosynthesis and Antimicrobial Action

**Figure 6:**
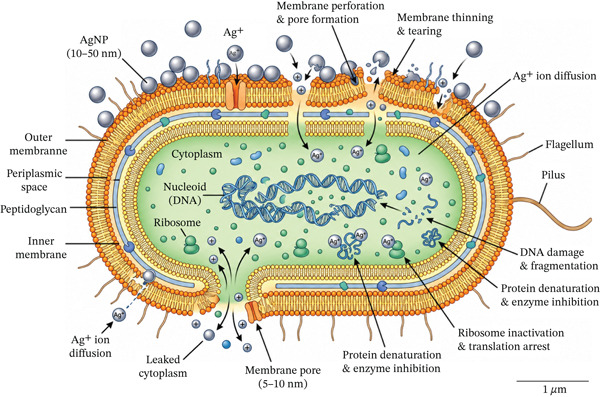
Proposed antimicrobial mechanisms of *P. eou*s‐derived AgNPs. The nanoparticles exert antimicrobial effects through membrane disruption, ROS generation, protein and DNA interference, and Ag^+^ ion release.

Based on the FTIR analysis, GC–MS profiling, and antimicrobial findings, a tentative mechanism for AgNPs biosynthesis and antimicrobial action is proposed. The biosynthesis of AgNPs using *P. eous* extract is primarily driven by phytochemicals that act as both reducing and stabilizing agents. Phenolic compounds, terpenoids, quinic acid derivatives, and other reducing biomolecules identified or inferred in the *P. eous* extract donate electrons to Ag^+^ ions, reducing them to metallic silver (Ag^0^) [23, 26]. This process is facilitated by functional groups such as hydroxyl (▬OH), carbonyl (C〓O), and amine (▬NH) groups, which were confirmed in the FTIR analysis [77]. These compounds have been reported to exhibit antimicrobial activity in the literature; however, the antimicrobial activity of the crude extract and AgNPs may be due to a combination of constituents and nanoparticle effects. Isolation and bioassay‐guided fractionation are needed to identify active components. Following reduction, these biomolecules adsorb onto the nanoparticle surface, forming a stabilizing capping layer that prevents aggregation and contributes to nanoparticle uniformity (Figure 6). This dual role of reducing and capping action explains the formation of spherical AgNPs observed in FE‐SEM images and the characteristic SPR peak at 420 nm [77, 78].

### 4.6. Study Limitations and Future Perspectives

Since all tested concentrations exhibited inhibition values exceeding 85%, the IC_50_ value was below the minimum concentration tested (0.1 mg mL^−1^). Therefore, future experiments should include lower concentration ranges to enable accurate IC_50_ estimation and more robust comparisons of antioxidant activity.

MeOH was used only for phytochemical extraction, and the MeOH filtrate was dried to constant weight under reduced pressure before assay; all bioassays used dried extract reconstituted in DMSO and included DMSO as a negative control. Crucially, AgNPs were synthesized using the aqueous extract (no MeOH present), washed repeatedly, and dried. Therefore, MeOH did not participate in nanoparticle formation, nor is it likely to account for the observed bioactivity of AgNPs. The comparison between the MeOH extract and biosynthesized AgNPs was intended to evaluate whether the nanoscale formulation enhances the biological activity associated with *P. eous*‐derived biomolecules, rather than to compare solvent‐equivalent extracts directly. Although aqueous and methanolic extraction differ in selectivity, both solvent systems have been shown to recover overlapping classes of bioactive metabolites from *Pleurotus* species, including phenolics and terpenoids, which are precisely the compound classes identified by GC–MS in the present study and known to serve as reducing and capping agents in nanoparticle biosynthesis [79–81]. Aqueous extracts are preferred for green synthesis of nanoparticles because they are environmentally friendly, clinically adaptable, biocompatible, cost‐effective, and provide water‐soluble mycochemicals that act as reducing and stabilizing agents [82]. In addition, because of instrument access limitations and the scarcity of the mushroom raw material, a comparison between aqueous extract spectra and nanoparticle spectra could not be included in the current study.

Although the biosynthesized AgNPs were characterized using UV–Vis, FTIR, XRD, and FE‐SEM analyses, parameters such as zeta potential, hydrodynamic size distribution (DLS), and transmission electron microscopy (TEM) imaging were not included in this study. Therefore, conclusions regarding nanoparticle colloidal stability, hydrodynamic diameter, and ultrastructural morphology should be interpreted cautiously. These were omitted due to facility limitations, but are planned for future optimization studies.

The agar well diffusion assay demonstrated quantifiable antibacterial and antifungal activity across a wide concentration range, providing a preliminary indication of the nanoparticles’ minimum inhibitory range. However, this study did not determine the precise minimum inhibitory concentration (MIC) values due to facility limitations. These are the fundamental next steps for establishing therapeutic relevance. While the differences observed among treatments were statistically significant (*p* < 0.05), zone‐of‐inhibition assays are diffusion‐dependent and may not directly represent MIC or MBC values. Future studies should also explore the mechanism of action and safety in vivo. Moreover, cytotoxicity testing on mammalian cell lines and the biocompatibility of the biosynthesized AgNPs remain to be established. Since AgNPs can exhibit dose‐dependent toxicity, comprehensive in vitro and in vivo safety evaluations are required before any therapeutic or biomedical applications can be considered.

## 5. Conclusion

The aqueous extract of *P. eous* reduced silver nitrate to crystalline, spherical AgNPs with a mean diameter of 39 ± 7.6 nm as confirmed by UV–Vis (SPR at 420 nm), XRD, FE‐SEM, and FTIR analyses. The methanolic extract exhibited strong DPPH radical‐scavenging activity (up to 97.39% inhibition at 0.7 mg mL^−1^) and a moderate total phenolic content (1.02 ± 0.07 mg GAE g^−1^ DW), consistent with the phenolic and terpenoid compounds identified by GC–MS. The biosynthesized AgNPs demonstrated broad‐spectrum antimicrobial activity against all tested bacterial and fungal pathogens, producing inhibition zones of 11.8–16.3 mm for bacteria and 13.3–14.3 mm for fungi at 250 *μ*g, with activity significantly greater than that of the crude extract and AgNO_3_ alone (*p* < 0.05), though lower than that of standard antibiotics and antifungals. Together, these results confirm that *P. eous* serves as an effective reducing and capping agent for the synthesis of green AgNPs and that the nanoformulation significantly enhances the antimicrobial potential of its biomolecules. MIC/MBC determination, cytotoxicity profiling, and in vivo efficacy studies are needed before any therapeutic application can be considered.

**Nomenclature**
AgNPs: silver nanoparticles
ANOVA: analysis of variance
DLS: Dynamic Light Scattering
DPPH: 2,2_−_Diphenyl_−_1_−_picrylhydrazyl
FE_−_SEM: field emission scanning electron microscopy
FTIR: Fourier transform infrared spectroscopy
IC_50_
: half maximal inhibitory concentration
MBC: minimum bactericidal concentration
MeOH: methanol
MIC: minimum inhibitory concentration
ROS: reactive oxygen species
TEM: transmission electron microscope
TPC: total phenolic content
UV–Vis: ultraviolet–visible spectroscopy
XRD: X_−_ray diffraction
*μ*g mL^−1^
: micrograms per milliliter

## Author Contributions

M.A.Q. (conceptualization, methodology, formal analysis, writing, visualization, and correspondence). S.R.K. (project administration, supervision, reviewing, and validation).

## Funding

No funding was received for this manuscript.

## Disclosure

All authors approved the manuscript before submission.

## Ethics Statement

Not applicable, this article does not contain any studies with human participants or animals.

## Consent

The authors have nothing to report.

## Conflicts of Interest

The authors declare no conflicts of interest.

## Supporting Information

Additional supporting information can be found online in the Supporting Information section.

## Supporting information


**Supporting Information 1** Table S1: Complete GC–MS chromatogram with retention times, peak areas, and similarity indices.

**Supporting Information 2** Table S2: Raw DPPH absorbance values used for percentage inhibition calculation.

**Supporting Information 3** Table S3: Raw antimicrobial inhibition zone measurements (triplicate values) used for statistical analysis.

## Data Availability

All generated and analyzed data during this study are included in this published article and its supporting information files.

## References

[1] Church N. A. and McKillip J. L. , Antibiotic Resistance Crisis: Challenges and Imperatives, Biologia. (2021) 76, no. 5, 1535–1550, 10.1007/s11756-021-00697-x.

[2] Krishnamoorthi R. , Mahalingam P. U. , and Malaikozhundan B. , Edible Mushroom Extract Engineered Ag NPs as Safe Antimicrobial and Antioxidant Agents With No Significant Cytotoxicity on Human Dermal Fibroblast Cells, Inorganic Chemistry Communications. (2022) 139, 109362, 10.1016/j.inoche.2022.109362.

[3] Obodai M. , Owusu E. , Shiwenger G. O. , Asante I. K. , and Dzomeku M. , "Phytochemical and Mineral Analysis of 12 Cultivated Oyster Mushrooms (Pleurotus species)." Advances in Life, Science and Technology. (2014) 26.

[4] Ahmed M. , Abdullah N. , and Nuruddin M. M. , Yield and Nutritional Composition of Oyster Mushrooms: An Alternative Nutritional Source for Rural People, Sains Malaysiana. (2016) 45, no. 11, 1609–1615.

[5] Kim J.-H. , Kim S.-J. , Park H.-R. , Choi J.-I. , Young-Cheoul J. , Nam K.-C. , Kim S.-J. , and Lee S.-C. , The Different Antioxidant and Anticancer Activities Depending on the Color of Oyster Mushrooms, Journal of Medicinal Plants Research. (2009) 3, no. 12, 1016–1020.

[6] Medihi N. I. , Haiyee Z. A. , Sukor R. , and Raseetha S. , Exploring the Functional Properties and Nutritional Values of Colored Oyster Mushrooms Species (Pleurotus, Agaricomycetes): A Review, International Journal of Medicinal Mushrooms. (2024) 26, no. 6, 25–38, 10.1615/IntJMedMushrooms.2024053563. 38808753

[7] Dash P. , Kar B. , Gochhi M. , Ghosh G. , Rai V. K. , Das C. , Pradhan D. , Rajwar T. K. , Halder J. , Dubey D. , and Manoharadas S. , Antimicrobial Properties of the Edible Pink Oyster Mushroom, Pleurotus eous: In-Vivo and In-Vitro Studies, Microbial Pathogenesis. (2024) 196, 106915, 10.1016/j.micpath.2024.106915, 39243990. 39243990

[8] Santos D. , Alves C. , Seckler M. M. , Ingle A. P. , Gupta I. , Galdiero S. , Galdiero M. , Gade A. , and Rai M. , Silver Nanoparticles: Therapeutical Uses, Toxicity, and Safety Issues, Journal of Pharmaceutical Sciences. (2014) 103, no. 7, 1931–1944, 10.1002/jps.24001, 24824033. 24824033

[9] Magar N. , Nepal P. , Magar A. T. , Magar J. T. , Oli H. B. , and Bhattarai D. P. , Green Synthesis of Silver Nanoparticles Using Camellia sinensis (tea) Leaves Extract: Characterization and Study of Its Antimicrobial Activities, Characterization and Application of Nanomaterials. (2026) 9, no. 1, 135–140, 10.24294/can8340.

[10] Khalandi B. , Asadi N. , Milani M. , Davaran S. , Abadi A. J. N. , Abasi E. , and Akbarzadeh A. , A Review on Potential Role of Silver Nanoparticles and Possible Mechanisms of Their Actions on Bacteria, Drug Research. (2017) 67, no. 2, 70–76, 10.1055/s-0042-113383. 27824432

[11] Sharma D. , Gulati S. S. , Sharma N. , and Chaudhary A. , Sustainable Synthesis of Silver Nanoparticles Using Various Biological Sources and Waste Materials: A Review, Emergent Materials. (2022) 5, no. 6, 1649–1678, 10.1007/s42247-021-00292-5.

[12] Malaikozhundan B. , Vijayakumar S. , Vaseeharan B. , Jenifer A. A. , Chitra P. , Prabhu N. M. , and Kannapiran E. , Two Potential Uses for Silver Nanoparticles Coated With Solanum nigrum Unripe Fruit Extract: Biofilm Inhibition and Photodegradation of Dye Effluent, Microbial Pathogenesis. (2017) 111, 316–324, 10.1016/j.micpath.2017.08.039, 28867634. 28867634

[13] Malaikozhundan B. , Vaseeharan B. , Vijayakumar S. , Sudhakaran R. , Gobi N. , and Shanthini G. , Antibacterial and Antibiofilm Assessment of Momordica charantia Fruit Extract Coated Silver Nanoparticle, Biocatalysis and Agricultural Biotechnology. (2016) 8, 189–196, 10.1016/j.bcab.2016.09.007.

[14] Mejia-Mendez J. L. , Reza-Zaldívar E. E. , Sanchez-Martinez A. , Ceballos-Sanchez O. , Navarro-López D. E. , Marcelo Lozano L. , Armendariz-Borunda J. , Tiwari N. , Jacobo-Velázquez D. A. , Sanchez-Ante G. , and López-Mena E. R. , Exploring the Cytotoxic and Antioxidant Properties of Lanthanide-Doped ZnO Nanoparticles: A Study With Machine Learning Interpretation, Journal of Nanobiotechnology. (2024) 22, no. 1, 10.1186/s12951-024-02957-9, 39523303. PMC11552316 39523303

[15] Ahmad F. , Ashraf N. , Ashraf T. , Zhou R.-B. , and Yin D.-C. , Biological Synthesis of Metallic Nanoparticles (MNPs) by Plants and Microbes: Their Cellular Uptake, Biocompatibility, and Biomedical Applications, Applied Microbiology and Biotechnology. (2019) 103, no. 7, 2913–2935, 10.1007/s00253-019-09675-5, 30778643. 30778643

[16] Du Y. and Yuan X. , Coupled Hybrid Nanoparticles for Improved Dispersion Stability of Nanosuspensions: A Review, Journal of Nanoparticle Research. (2020) 22, no. 9, 10.1007/s11051-020-04991-8.

[17] Casals E. , Gusta M. F. , Bastus N. , Rello J. , and Puntes V. , Silver Nanoparticles and Antibiotics: A Promising Synergistic Approach to Multidrug-Resistant Infections, Microorganisms. (2025) 13, no. 4, 10.3390/microorganisms13040952, 40284788. PMC12029289 40284788

[18] Yadav S. , Nadar T. , Lakkakula J. , and Wagh N. S. , Biogenic Synthesis of Nanomaterials: Bioactive Compounds as Reducing and Capping Agents, Biogenic Nanomaterials for Environmental Sustainability: Principles, Practices, and Opportunities, 2024, Springer International Publishing, 147–188, 10.1007/978-3-031-45956-6_6.

[19] Vaseghi Z. , Nematollahzadeh A. , and Tavakoli O. , Green Methods for the Synthesis of Metal Nanoparticles Using Biogenic Reducing Agents: A Review, Reviews in Chemical Engineering. (2018) 34, no. 4, 529–559, 10.1515/revce-2017-0005.

[20] Fareid M. A. , El-Sherbiny G. M. , Askar A. A. , Abdelaziz A. M. , Hegazy A. M. , Aziz R. A. , and Hamada F. A. , Impeding Biofilm-Forming Mediated Methicillin-Resistant Staphylococcus aureus and Virulence Genes Using a Biosynthesized Silver Nanoparticle–Antibiotic Combination, Biomolecules. (2025) 15, no. 2, 10.3390/biom15020266, 40001569. PMC11852608 40001569

[21] Hayat P. , Khan I. , Rehman A. , Jamil T. , Hayat A. , Rehman M. U. , Ullah N. , Sarwar A. , Alharbi A. A. , Dablool A. S. , Daudzai Z. , Alamri A. S. , Alhomrani M. , and Aziz T. , Myogenesis and Analysis of Antimicrobial Potential of Silver Nanoparticles (AgNPs) Against Pathogenic Bacteria, Molecules. (2023) 28, no. 2, 10.3390/molecules28020637, 36677695. PMC9863364 36677695

[22] Bhardwaj K. , Sharma A. , Tejwan N. , Bhardwaj S. , Bhardwaj P. , Nepovimova E. , Shami A. , Kalia A. , Kumar A. , Abd-Elsalam K. A. , and Kuča K. , Pleurotus Macrofungi-Assisted Nanoparticle Synthesis and Its Potential Applications: A Review, Journal of Fungi. (2020) 6, no. 4, 10.3390/jof6040351, 33317038. PMC7770583 33317038

[23] Dhachinamoorthi D. , Tharun Sai C. , Kumari R. , and Sasidhar B. , Bioinspired Methods for Silver Nanoparticle Production: Applications in Biomedicine, Journal of Innovations in Applied Pharmaceutical Science (JIAPS). (2024) 2, 9–16, 10.37022/jiaps.v9i1.567.

[24] Brand-Williams W. , Cuvelier M.-E. , and Berset C. L. W. T. , Use of a Free Radical Method to Evaluate Antioxidant Activity, LWT-Food Science and Technology. (1995) 28, no. 1, 25–30, 10.1016/S0023-6438(95)80008-5.

[25] Pérez M. , Dominguez-López I. , and Lamuela-Raventós R. M. , The Chemistry Behind the Folin–Ciocalteu Method for the Estimation of (Poly) Phenol Content in Food: Total Phenolic Intake in a Mediterranean Dietary Pattern, Journal of Agricultural and Food Chemistry. (2023) 71, no. 46, 17543–17553, 10.1021/acs.jafc.3c04022, 37948650. 37948650 PMC10682990

[26] Balouiri M. , Sadiki M. , and Ibnsouda S. K. , Methods for In Vitro Evaluating Antimicrobial Activity: A Review, Journal of Pharmaceutical Analysis. (2016) 6, no. 2, 71–79, 10.1016/j.jpha.2015.11.005, 29403965. 29403965 PMC5762448

[27] Singh J. , Luqman S. , and Meena A. , Carvacrol as a Prospective Regulator of Cancer Targets/Signalling Pathways, Current Molecular Pharmacology. (2023) 16, no. 5, 542–558, 10.2174/1874467215666220705142954, 35792130. 35792130

[28] Sampaio L. A. , Pina L. T. S. , Serafini M. R. , Tavares D. D. S. , and Guimaraes A. G. , Antitumor Effects of Carvacrol and Thymol: A Systematic Review, Frontiers in Pharmacology. (2021) 12, 702487, 10.3389/fphar.2021.702487, 34305611. 34305611 PMC8293693

[29] Lee J.-H. , Kim Y.-G. , Khadke S. K. , and Lee J. , Antibiofilm and Antifungal Activities of Medium-Chain Fatty Acids Against Candida albicans via Mimicking of the Quorum-Sensing Molecule Farnesol, Microbial Biotechnology. (2021) 14, no. 4, 1353–1366, 10.1111/1751-7915.13710, 33252828. 33252828 PMC8313291

[30] Jang Y.-W. , Jung J.-Y. , Lee I.-K. , Kang S.-Y. , and Yun B.-S. , Nonanoic Acid, an Antifungal Compound From Hibiscus syriacus Ggoma, Mycobiology. (2012) 40, no. 2, 145–146, 10.5941/MYCO.2012.40.2.145, 22870060. 22870060 PMC3408307

[31] Ercan L. and Doğru M. , Antioxidant and Antimicrobial Capacity of Quinic aCid, Bitlis Eren Üniversitesi Fen Bilimleri Dergisi. (2022) 11, no. 4, 1018–1025, 10.17798/bitlisfen.1167047.

[32] Benali T. , Bakrim S. , Ghchime R. , Benkhaira N. , El Omari N. , Balahbib A. , Taha D. , Zengin G. , Hasan M. M. , Bibi S. , and Bouyahya A. , Pharmacological Insights Into the Multifaceted Biological Properties of Quinic Acid, Biotechnology and Genetic Engineering Reviews. (2024) 40, no. 4, 3408–3437, 10.1080/02648725.2022.2122303, 36123811. 36123811

[33] Ekhtiar M. , Ghasemi-Dehnoo M. , Azadegan-Dehkordi F. , and Bagheri N. , Evaluation of Anti-Inflammatory and Antioxidant Effects of Ferulic Acid and Quinic Acid on Acetic Acid-Induced Ulcerative Colitis in Rats, Journal of Biochemical and Molecular Toxicology. (2025) 39, no. 2, e70169, 10.1002/jbt.70169, 39957712. 39957712

[34] Su P. , Shi Y. , Wang J. , Shen X. , and Zhang J. , Anticancer Agents Derived From Natural Cinnamic Acids, Anti-Cancer Agents in Medicinal Chemistry. (2015) 15, no. 8, 980–987, 10.2174/1871520615666150130111120. 25634446

[35] Feng L.-S. , Cheng J.-B. , Wen-Qi S. , Li H.-Z. , Xiao T. , Chen D.-A. , and Zhang Z.-L. , Cinnamic Acid Hybrids as Anticancer Agents: A Mini-Review, Archiv der Pharmazie. (2022) 355, no. 7, e2200052, 10.1002/ardp.202200052, 35419808. 35419808

[36] Atni O. K. , Munir E. , and Pasaribu N. , Diversity of Bioactive Compounds From Parmotrema xanthinum as Antimicrobial Potential Through In-Vitro and In-Silico Assessment, Biodiversitas: Journal of Biological Diversity. (2024) 25, no. 11, 10.13057/biodiv/d251143.

[37] FuJi W. and KyungSeun Y. , Studies on the Chemical Components and Antibacterial Activities of Essential Oil From Cassia Bark, China Condiment. (2010) 10, 94–96.

[38] Kachur K. and Suntres Z. , The Antibacterial Properties of Phenolic Isomers, Carvacrol and Thymol, Critical Reviews in Food Science and Nutrition. (2020) 60, no. 18, 3042–3053, 10.1080/10408398.2019.1675585, 31617738. 31617738

[39] Hajibonabi A. , Yekani M. , Sharifi S. , Nahad J. S. , Dizaj S. M. , and Memar M. Y. , Antimicrobial Activity of Nanoformulations of Carvacrol and Thymol: New Trend and Applications, OpenNano. (2023) 13, 100170, 10.1016/j.onano.2023.100170.

[40] Li Q. , Shuna Y. , Han J. , Jiulin W. , You L. , Shi X. , and Wang S. , Synergistic Antibacterial Activity and Mechanism of Action of Nisin/Carvacrol Combination Against Staphylococcus aureus and Their Application in the Infecting Pasteurized Milk, Food Chemistry. (2022) 380, 132009, 10.1016/j.foodchem.2021.132009, 35077986. 35077986

[41] Heena S. K. , Kaur V. , Panwar H. , Sharma P. , and Jangra R. , Isolation of Quinic Acid From Dropped Citrus reticulata Blanco Fruits: Its Derivatization, Antibacterial Potential, Docking Studies, and ADMET Profiling, Frontiers in Chemistry. (2024) 12, 1372560, 10.3389/fchem.2024.1372560. 38698937 PMC11064019

[42] Bai J. , Yanping W. , Wang X. , Liu X. , Zhong K. , Huang Y. , Chen Y. , and Gao H. , In *vitro* and in *vivo* characterization of the Antibacterial Activity and Membrane Damage Mechanism of Quinic Acid against *Staphylococcus aureus* , Journal of Food Safety. (2018) 38, no. 1, e12416, 10.1111/jfs.12416.

[43] Jiang Y.-H. , Shi X.-C. , Ting W. , Hao D. , Pang Y.-B. , Zhou R. , Yin H.-P. et al., Synthesis and Antifungal Activity of Novel Amide Derivatives From Quinic Acid Against the Sweet Potato Pathogen Ceratocystis fimbriata, Pest Management Science. (2025) 81, no. 3, 1286–1298, 10.1002/ps.8527, 39501798. 39501798

[44] Krátký M. , Konečná K. , Brokešová K. , Maixnerová J. , Trejtnar F. , and Vinšová J. , Optimizing the Structure of (Salicylideneamino) Benzoic Acids: Towards Selective Antifungal and Anti-Staphylococcal Agents, European Journal of Pharmaceutical Sciences. (2021) 159, 105732, 10.1016/j.ejps.2021.105732, 33493669. 33493669

[45] Chen J. , Jia X. , Youjun H. , Zhao X. , Cheng Y. , Li L. , Zhong S. , You J. , and Zou T. , Benzoic Acid as a Dietary Supplement Mitigates Inflammation and Intestinal Injury in Acute Enterotoxigenic Escherichia coli-Infected Mice Without Adverse Effects in Healthy Mice, Food & Function. (2025) 16, no. 8, 3195–3210, 10.1039/D5FO00514K, 40190113. 40190113

[46] Zarafu I. R. I. N. A. , Pătrașcu B. I. A. N. C. A. , Măruțescu L. U. M. I. N. I. Ț. A. , Bleotu C. , Limban C. , Tatibouet A. , Chifiriuc M. C. , Nuță D. I. A. N. A. C. A. M. E. L. I. A. , and Ioniță P. E. T. R. E. , Bioevaluation of the Antimicrobial and Anti-Proliferative Potential of Some Derivatives of 3, 5-Dinitro-4-Methoxyamino-Benzoic Acid, Farmacia. (2020) 68, no. 1, 8–14, 10.31925/farmacia.2020.1.2.

[47] Sova M. , Antioxidant and Antimicrobial Activities of Cinnamic Acid Derivatives, Mini Reviews in Medicinal Chemistry. (2012) 12, no. 8, 749–767, 10.2174/138955712801264792. 22512578

[48] Jităreanu A. , Tătărîngă G. , Zbancioc A.-M. , Tuchiluş C. , and Stănescu U. , Antimicrobial Activity of Some Cinnamic Acid Derivatives, Revista Medico-chirurgicala a Societatii de Medici si Naturalisti din Iasi. (2011) 115, no. 3, 965–971, 22046817. 22046817

[49] Zawiła T. , Swolana D. , Rok J. , Rzepka Z. , and Wojtyczka R. D. , Evaluation of the Antibacterial Activity of Cinnamic Acid and Its Derivatives: Synergistic Effects With Cloxacillin, Molecules. (2025) 30, no. 3, 10.3390/molecules30030660, 39942764. PMC11820451 39942764

[50] Guzman J. D. , Natural Cinnamic Acids, Synthetic Derivatives and Hybrids With Antimicrobial Activity, Molecules. (2014) 19, no. 12, 19292–19349, 10.3390/molecules191219292, 25429559. 25429559 PMC6271800

[51] Kendre B. V. , Landge M. G. , and Bhusare S. R. , Synthesis and Biological Evaluation of Some Novel Pyrazole, Isoxazole, Benzoxazepine, Benzothiazepine and Benzodiazepine Derivatives Bearing an Aryl Sulfonate Moiety as Antimicrobial and Anti-Inflammatory Agents, Arabian Journal of Chemistry. (2019) 12, no. 8, 2091–2097, 10.1016/j.arabjc.2015.01.007.

[52] Gąsecka M. , Mleczek M. , Siwulski M. , and Niedzielski P. , Phenolic Composition and Antioxidant Properties of Pleurotus ostreatus and Pleurotus eryngii Enriched With Selenium and Zinc, European Food Research and Technology. (2016) 242, no. 5, 723–732, 10.1007/s00217-015-2580-1.

[53] Kılıç C. , Gürgen A. , Yıldız S. , Can Z. , and Değirmenci A. , Total Phenolics, Tannin Contents, Antioxidant Properties, Protein and Sensory Analysis of Pleurotus ostreatus, Pleurotus citrinopileatus and Pleurotus djamor Cultivated on Different Sawdusts, Maderas. Ciencia y tecnología. (2023) 26, 10.22320/s0718221x/2024.20.

[54] Radzki W. , Tutaj K. , Skrzypczak K. , Michalak-Majewska M. , and Gustaw W. , Ethanolic Extracts of Six Cultivated Mushrooms as a Source of Bioactive Compounds, Applied Sciences. (2024) 14, no. 1, 10.3390/app14010066.

[55] Krupodorova T. , Barshteyn V. , Gafforov Y. , Rašeta M. , Zaichenko T. , and Blume Y. , Comparative Evaluation of Free Radical Scavenging Activity and Total Metabolite Profiles Among 30 Macrofungi Species, Bioresources and Bioprocessing. (2025) 12, no. 1, 10.1186/s40643-025-00841-4, 39982581. PMC11845661 39982581

[56] Effiong M. E. , Umeokwochi C. P. , Afolabi I. S. , and Chinedu S. N. , Comparative Antioxidant Activity and Phytochemical Content of Five Extracts of Pleurotus ostreatus (Oyster Mushroom), Scientific Reports. (2024) 14, no. 1, 10.1038/s41598-024-54201-x, 38361132. PMC10869810 38361132

[57] Norizham H. , Anuar M. F. , Hilmi A. , Loh D. W. H. , Marimuthu S. , and Ahmad R. , Study on Phytochemicals and Antioxidants of Pleurotus sp. Cultivated Through Submerged Fermentation, Semarak International Journal of Petroleum and Chemical Engineering. (2024) 1, no. 1, 1–14, 10.37934/sijpce.1.1.114.

[58] Yim H. S. , Chye F. Y. , Tan C. T. , Ng Y. C. , and Ho C. W. , Antioxidant Activities and Total Phenolic Content of Aqueous Extract of Pleurotus ostreatus (Cultivated Oyster Mushroom), Malaysian Journal of Nutrition. (2010) 16, no. 2, 281–291, 22691933. 22691933

[59] Rahimah S. B. , Djunaedi D. D. , Soeroto A. Y. , and Bisri T. , The The Phytochemical Screening, Total Phenolic Contents and Antioxidant Activities In Vitro of White Oyster Mushroom (Pleurotus ostreatus) Preparations, Journal of Medical Sciences. (2019) 7, no. 15, 2404–2412, 10.3889/oamjms.2019.741. PMC6814466 31666837

[60] Velika B. and Kron I. , Antioxidant Properties of Benzoic Acid Derivatives Against Superoxide Radical, Free Radicals and Antioxidants. (2012) 2, no. 4, 62–67, 10.5530/ax.2012.4.11.

[61] Pais J. P. , Magalhães M. , Antoniuk O. , Barbosa I. , Freire R. , Pires D. , Valente E. , Testa B. , Anes E. , and Constantino L. , Benzoic Acid Derivatives as Prodrugs for the Treatment of Tuberculosis, Pharmaceuticals. (2022) 15, no. 9, 10.3390/ph15091118, 36145340. PMC9502840 36145340

[62] Manuja R. , Sachdeva S. , Jain A. , and Chaudhary J. , A Comprehensive Review on Biological Activities of p-Hydroxy Benzoic Acid and Its Derivatives, International Journal of Pharmaceutical Sciences Review and Research. (2013) 22, no. 2, 109–115.

[63] Owaid M. N. , Green Synthesis of Silver Nanoparticles by Pleurotus (Oyster Mushroom) and Their bioactivity: Review, Environmental Nanotechnology, Monitoring & Management. (2019) 12, 100256, 10.1016/j.enmm.2019.100256.

[64] Musa S. F. , Yeat T. S. , Kamal L. Z. M. , Tabana Y. M. , Ahmed M. A. , El Ouweini A. , Lim V. , Keong L. C. , and Sandai D. , Pleurotus Sajor-Caju can be Used to Synthesize Silver Nanoparticles With Antifungal Activity Against Candida albicans, Journal of the Science of Food and Agriculture. (2018) 98, no. 3, 1197–1207, 10.1002/jsfa.8573, 28746729. 28746729

[65] Irshad A. , Sarwar N. , Sadia H. , Riaz M. , Sharif S. , Shahid M. , and Khan J. A. , Silver Nano-Particles: Synthesis and Characterization by Using Glucans Extracted From Pleurotus Ostreatus, Nanoscience. (2020) 10, no. 8, 3205–3214, 10.1007/s13204-019-01103-4.

[66] Hassanzadeh-Tabrizi S. A. , Precise Calculation of Crystallite Size of Nanomaterials: A Review, Journal of Alloys and Compounds. (2023) 968, 171914, 10.1016/j.jallcom.2023.171914.

[67] Ballottin D. , Fulaz S. , Souza M. L. , Corio P. , Rodrigues A. G. , Souza A. O. , Gaspari P. M. , Gomes A. F. , Gozzo F. , and Tasic L. , Elucidating Protein Involvement in the Stabilization of the Biogenic Silver Nanoparticles, Nanoscale Research Letters. (2016) 11, no. 1, 10.1186/s11671-016-1538-y, 27356560. PMC4927534 27356560

[68] Naraian R. and Abhishek A. K. B. , Green Synthesis and Characterization of Silver NPs Using Oyster Mushroom Extract for Antibacterial Efficacy, Journal of Chemistry, Environmental Sciences and its Applications. (2020) 7, no. 1, 13–18, 10.15415/jce.2020.71003.

[69] Maurya S. , Bhardwaj A. K. , Gupta K. K. , Agarwal S. , Kushwaha A. , Vk C. , Pathak R. K. , Gopal R. , Uttam K. N. , Singh A. K. , and Verma V. , Green Synthesis of Silver Nanoparticles Using Pleurotus and Its Bactericidal Activity, Cellular and Molecular Biology. (2016) 62, 10.4172/1165-158X.1000131.

[70] Eltarahony M. , Zaki S. , ElKady M. , and Abd-El-Haleem D. , Biosynthesis, Characterization of Some Combined Nanoparticles, and Its Biocide Potency Against a Broad Spectrum of Pathogens, Journal of Nanomaterials. (2018) 2018, no. 1, 5263814, 10.1155/2018/5263814.

[71] Al-Dbass A. M. , Al Daihan S. , Al-Nasser A. A. , Al-Suhaibani L. S. , Almusallam J. , Alnwisser B. I. , Saloum S. , Alotaibi R. S. , Alessa L. A. , and Bhat R. S. , Biogenic Silver Nanoparticles From Two Varieties of Agaricus bisporus and Their Antibacterial Activity, Molecules. (2022) 27, no. 21, 10.3390/molecules27217656, 36364482. PMC9654042 36364482

[72] Deeb E. , Bahig A. , Faheem G. G. , and Bakhit M. S. , Biosynthesis of Silver Nanoparticles by *Talaromyces funiculosus* for Therapeutic Applications and Safety Evaluation, Scientific Reports. (2025) 15, no. 1, 10.1038/s41598-025-95899-7, 40258887. PMC12012204 40258887

[73] Ezebialu C. U. , Eze E. A. , Engwa G. A. , and Udeh A. S. , Synergic Antibacterial Activity of Some Wild Mushroom Extracts With Some Antibiotics Against Resistant Gram negative Bacteria, International Journal of Pharmaceutical Research. (2021) 13, no. 3, 1482–1490, 10.31838/ijpr/2021.13.03.210.

[74] Ghasemi S. , Harighi B. , and Ashengroph M. , Biosynthesis of Silver Nanoparticles Using Pseudomonas canadensis, and Its Antivirulence Effects Against Pseudomonas tolaasii, Mushroom Brown Blotch Agent, Scientific Reports. (2023) 13, no. 1, 10.1038/s41598-023-30863-x, 36871050. PMC9985599 36871050

[75] Mussin J. and Giusiano G. , Biogenic Silver Nanoparticles as Antifungal Agents, Frontiers in Chemistry. (2022) 10, 1023542, 10.3389/fchem.2022.1023542, 36277355. 36277355 PMC9583421

[76] Andrejč C. , Darija Ž. K. , and Marevci M. K. , Antioxidant, Antibacterial, Antitumor, Antifungal, Antiviral, Anti-Inflammatory, and Nevro-Protective Activity of Ganoderma lucidum: An Overview, Frontiers in Pharmacology. (2022) 13, 934982, 10.3389/fphar.2022.934982, 35935849. 35935849 PMC9353308

[77] Ullah S. , Khalid R. , Rehman M. F. , Irfan M. I. , Abbas A. , Alhoshani A. , Anwar F. , and Amin H. M. A. , Biosynthesis of Phyto-Functionalized Silver Nanoparticles Using Olive Fruit Extract and Evaluation of Their Antibacterial and Antioxidant Properties, Frontiers in Chemistry. (2023) 11, 1202252, 10.3389/fchem.2023.1202252. 37324561 PMC10262211

[78] Dawadi S. , Katuwal S. , Gupta A. , Lamichhane U. , Thapa R. , Jaisi S. , Lamichhane G. , Bhattarai D. P. , and Parajuli N. , Current Research on Silver Nanoparticles: Synthesis, Characterization, and Applications, Journal of Nanomaterials. (2021) 2021, no. 1, 6687290, 10.1155/2021/6687290.

[79] Cirlincione F. , Vurro F. , Ailoaiei A.-M. , Shahrivari-Baviloliaei S. , Difonzo G. , Viapiana A. , Plenis A. , Pasqualone A. , and Gargano M. L. , Antioxidant and Aromatic Properties of Aqueous Extracts of Pleurotus nebrodensis as Potential Food Ingredients, Foods. (2026) 15, no. 2, 10.3390/foods15020296, 41596895. PMC12841083 41596895

[80] Ganesan G. , Selvam G. , Varatharasu A. , and Annamalai P. , Physico-Chemical and Qualitative, Quantitative Chemical Compounds Analysis of *Pleurotus florida* in Thanjavur, Tamilnadu, India, International Journal of Life Science and Pharma Res. (2023) 13, no. 2, L8–L17, 10.22376/ijlpr.2023.13.2.L8-L17.

[81] Gashaw G. and Getu A. , Phytochemical Characteristics Evaluation of Pleurotus Species Cultivated on Agricultural Wastes in Chiro, Ethiopia, Indian Journal of Agricultural Research. (2021) 55, no. 3, 10.18805/IJARe.A-526.

[82] Vanlalveni C. , Lallianrawna S. , Biswas A. , Selvaraj M. , Changmai B. , and Rokhum S. L. , Green Synthesis of Silver Nanoparticles Using Plant Extracts and Their Antimicrobial Activities: A Review of Recent literature, RSC Advances. (2021) 11, no. 5, 2804–2837, 10.1039/D0RA09941D. 35424248 PMC8694026

