# Supplementary material for: Green Synthesis and Characterization of Silver Nanoparticles Using Pleurotus eous and Their Antioxidant and Antimicrobial Activities
Source: Int J Microbiol. 2026 Aug 6;2026:4151399. doi: 10.1155/ijm/4151399 (PMC13448151; doi:10.1155/ijm/4151399)

Sample Information

Analyzed by	: Admin
Analyzed	: 17-12-2024 12:12:55 PM
Sample Type	: Unknown
Level #	: 1
Sample Name	: MS-S2
Sample ID	: 024/551
IS Amount	: [1]=1
Sample Amount	: 1
Dilution Factor	: 1
Vial #	: 8
Injection Volume	: 1.00
Data File	: D:\MIT GCMS DATA 2\phytochemical\024551.qgd
Org Data File	: D:\MIT GCMS DATA 2\phytochemical\024551.qgd
Method File	: D:\Phytochemical profile extract.qgm
Org Method File	: D:\Phytochemical profile extract.qgm
Report File	:
Tuning File	: D:\MIT GCMS DATA\Tuning files\tuning.11.07.2023.qgt
Modified by	: Admin
Modified	: 17-12-2024 12:48:56 PM

Chromatogram MS-S2 D:\MIT GCMS DATA 2\phytochemical\024551.qgd

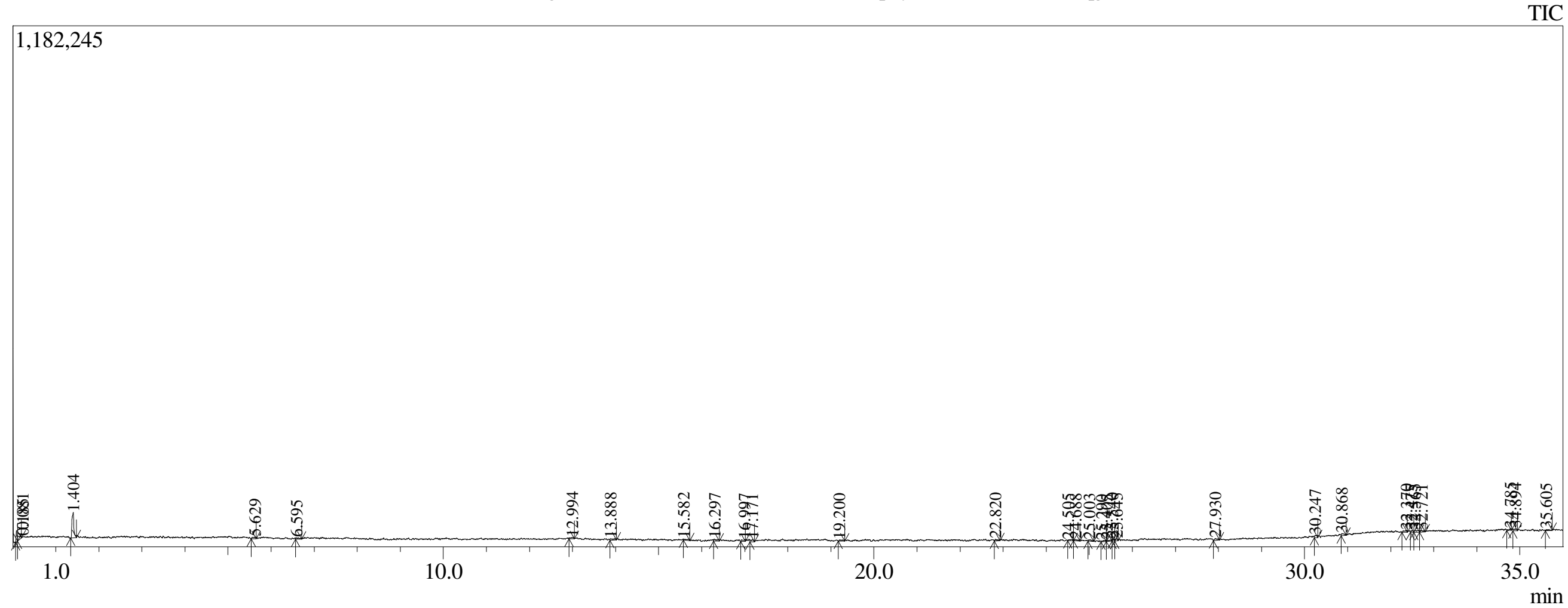

Peak Report TIC

Peak#	R.Time	I.Time	F.Time	Area	Area%	Height	Height%	Name
1	0.081	0.065	0.110	36333	4.9787	25541	13.67	3-Nonynoic acid
2	0.185	0.110	0.200	38109	5.2220	5206	2.79	2-(Triethoxysilyl)propylamine
3	1.404	1.345	1.475	178160	24.4130	57325	30.68	2-Chloroethyl methyl sulfoxide
4	5.629	5.535	5.655	19174	2.6274	3974	2.13	Quinic acid
5	6.595	6.570	6.710	16311	2.2351	3113	1.67	Eseroline, N,N-diethylcarbamate(ester)
6	12.994	12.915	13.045	15466	2.1193	2926	1.57	2-methylidene-6,10,14-trimethylpen2-methylid
7	13.888	13.865	14.020	20674	2.8329	3446	1.84	Thiosulfuric acid S-2-[[4-[[-1-pyrrolidinyl]carl
8	15.582	15.570	15.725	15046	2.0617	2353	1.26	Benzenesulfonamide, 4-chloro-3-nitro-
9	16.297	16.275	16.410	21328	2.9225	3783	2.02	Bicyclo[2.2.1]heptan-2-one, 4-methoxy-1,7,7-
10	16.997	16.905	17.010	18815	2.5782	4690	2.51	Acetonitrile, 2-[(1-ethyl-1H-1,2,3,4-tetrazol-5-
11	17.171	17.115	17.225	21681	2.9709	4732	2.53	Pregn-5-en-20-one, 3-(acetyloxy)-, 20-oxime,
12	19.200	19.170	19.325	13494	1.8491	2298	1.23	Diethylmalonic acid, hexyl 2,2,3,3,4,4,5,5-octa
13	22.820	22.795	22.925	15667	2.1468	3476	1.86	Acetamide, N-methyl-N-[4-[4-fluoro-1-hexahy
14	24.505	24.495	24.635	17389	2.3828	1834	0.98	Silane, diphenylcitronellyloxyhexyloxy-
15	24.688	24.635	24.785	17018	2.3320	3394	1.82	Pteridine, 2-ethoxy-4-(trifluoromethyl)-
16	25.003	24.970	25.105	14537	1.9920	3057	1.64	1-Ethenyl(dimethyl)silyloxynaphthalene
17	25.290	25.275	25.395	16096	2.2056	2604	1.39	2(3H)-Naphthalenone, 4,4a,5,6,7,8-hexahydro
18	25.405	25.395	25.530	19782	2.7107	2913	1.56	trans-2,3-Epoxyoctane
19	25.540	25.530	25.595	13671	1.8733	4477	2.40	Cyclopropa[3',4']benz[1',2':4,5]azuleno[1,8a-d
20	25.645	25.595	25.670	14531	1.9912	4076	2.18	Aziprotryne
21	27.930	27.885	28.020	20004	2.7411	4212	2.25	Bis(tributyltin)
22	30.247	30.225	30.305	19721	2.7023	5016	2.68	(-)-Isolongifolol, methyl ether
23	30.868	30.850	30.965	22737	3.1156	5235	2.80	2-(4-Nitrophenyl)succinic acid
24	32.370	32.260	32.385	23192	3.1780	4517	2.42	Cholestane-3,7-diol, 3-acetate, (3.beta.,5.alpha
25	32.475	32.460	32.540	13247	1.8152	3352	1.79	3,4-Dimethoxycinnamic acid, TMS derivative
26	32.565	32.540	32.670	25170	3.4490	4462	2.39	Benzoic acid, 2-hydroxy-4-methoxy-3,5,6-trim
27	32.721	32.670	32.800	17390	2.3829	3551	1.90	Trimethylsilyl 3-methyl-4-[(trimethylsilyl)oxy]
28	34.785	34.695	34.800	13573	1.8599	4531	2.42	Naphthalene, decahydro-1,4-dimethoxy-, (1.al
29	34.894	34.840	34.940	16929	2.3198	4312	2.31	Carvacrol, TBDMS derivative
30	35.605	35.595	35.750	14529	1.9909	2466	1.32	2-Cyclohexylamino-3,5-dinitropyridine

Peak#	R.Time	I.Time	F.Time	Area	Area%	Height	Height%	Name
				729774	100.0000	186872	100.00	

Library

<< Target >>
Line#:1 R.Time:0.080(Scan#:17) MassPeaks:330
RawMode:Averaged 0.075-0.085(16-18) BasePeak:52.00(397)
BG Mode:Calc. from Peak Group 1 - Event 1 Scan

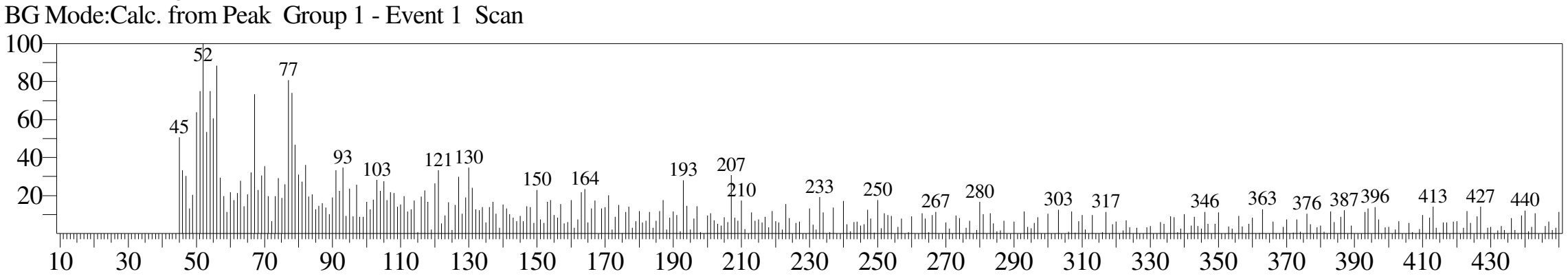

Hit#:1 Entry:28117 Library:NIST14.lib
SI:68 Formula:C9H14O2 CAS:56630-33-2 MolWeight:154 RetIndex:1290
CompName:3-Nonynoic acid

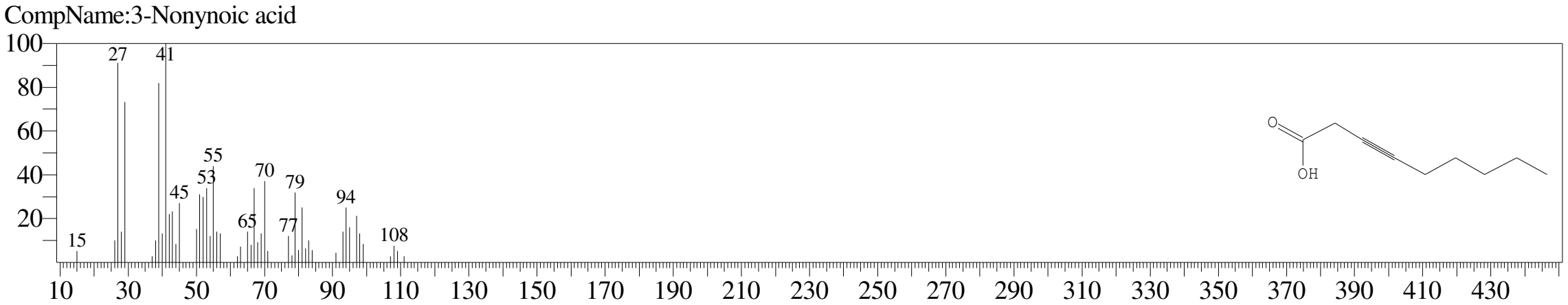

Hit#:2 Entry:35412 Library:NIST14.lib
SI:67 Formula:C11H16O CAS:21841-29-2 MolWeight:164 RetIndex:1328
CompName:1(2H)-Naphthalenone, 3,4,4a,5,8,8a-hexahydro-8a-methyl-, trans-

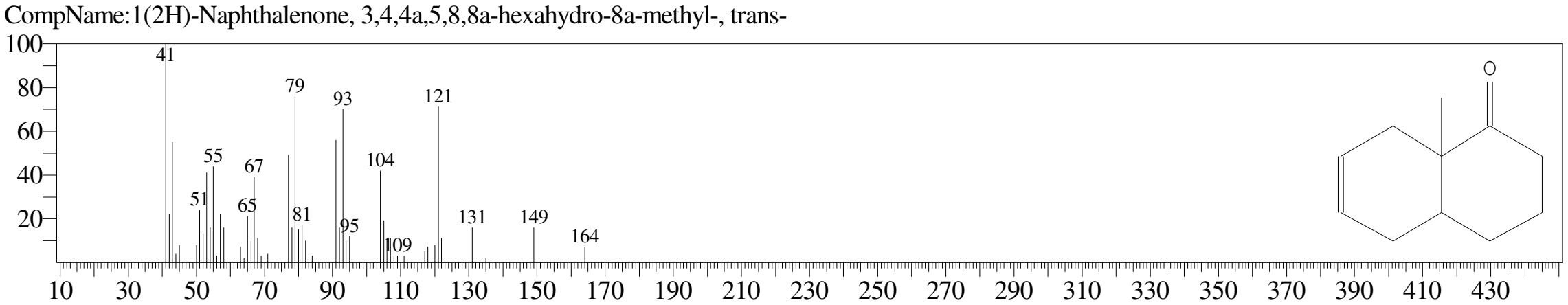

Hit#:3 Entry:47036 Library:NIST14.lib
SI:66 Formula:C9H13N3O CAS:68344-44-5 MolWeight:179 RetIndex:1824
CompName:Hydrazinecarboxamide, 2-(2,6-cyclooctadien-1-ylidene)-

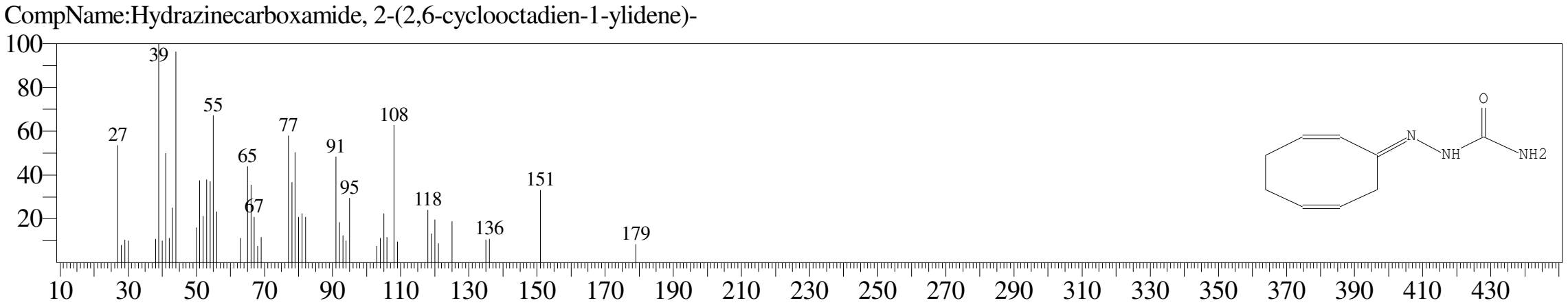

<< Target >>
Line#:2   R.Time:0.185(Scan#:38) MassPeaks:203
RawMode:Averaged 0.180-0.190(37-39) BasePeak:116.00(78)
BG Mode:Calc. from Peak Group 1 - Event 1 Scan

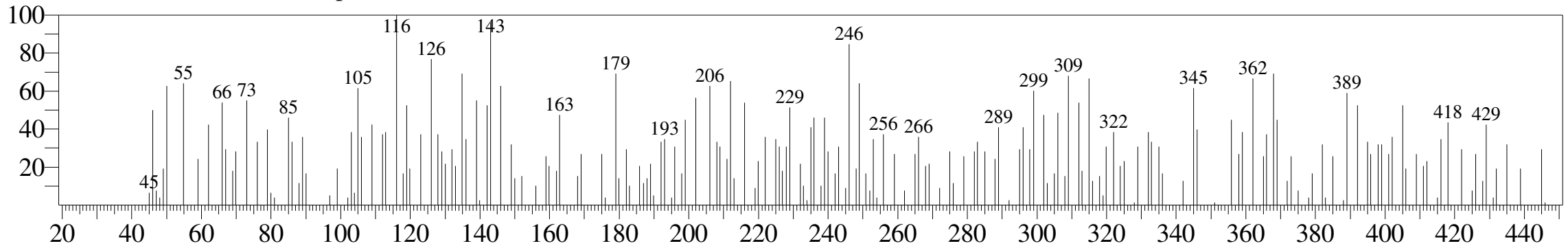

Hit#:1 Entry:84144 Library:NIST14.lib
SI:26 Formula:C9H23NO3Si CAS:0-00-0 MolWeight:221 RetIndex:1232
CompName:2-(Triethoxysilyl)propylamine

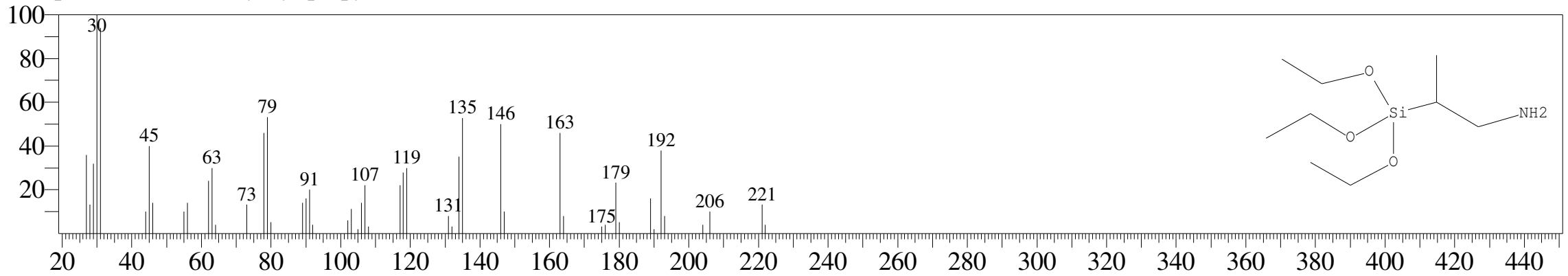

Hit#:2 Entry:77959 Library:NIST14.lib
SI:25 Formula:C11H12Cl2 CAS:0-00-0 MolWeight:214 RetIndex:1439
CompName:1,4-Dimethyl-2-(1',1'-dichloroallyl)benzene

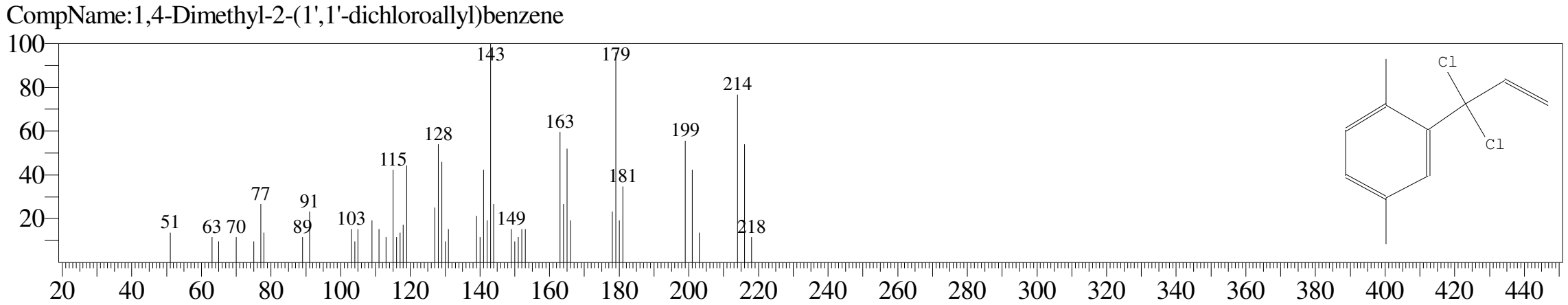

Hit#:3 Entry:221115 Library:NIST14.lib
SI:24 Formula:C25H38O2 CAS:58072-57-4 MolWeight:370 RetIndex:2375
CompName:3,5-Cyclochol-23-yn-22-ol, 6-methoxy-, (3.beta.,5.alpha.)-

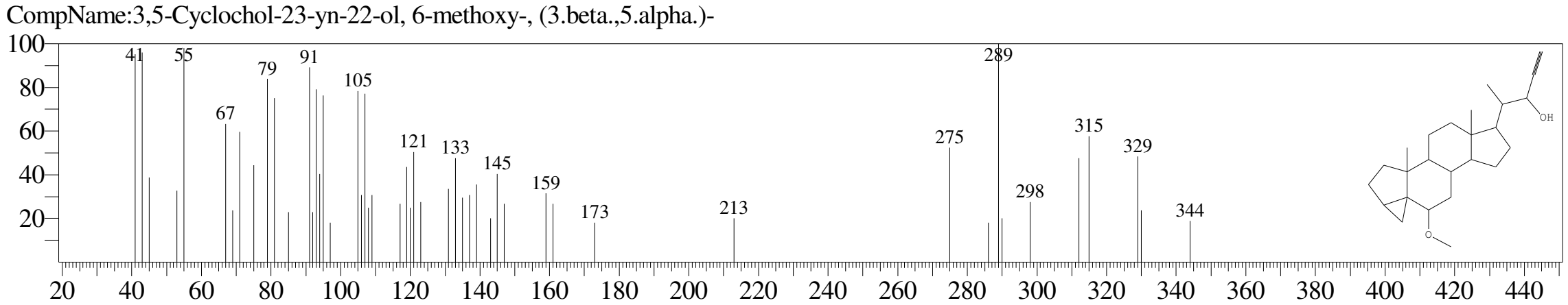

<< Target >>
Line#:3   R.Time:1.405(Scan#:282) MassPeaks:183
RawMode:Averaged 1.400-1.410(281-283) BasePeak:63.05(19843)
BG Mode:Calc. from Peak Group 1 - Event 1 Scan

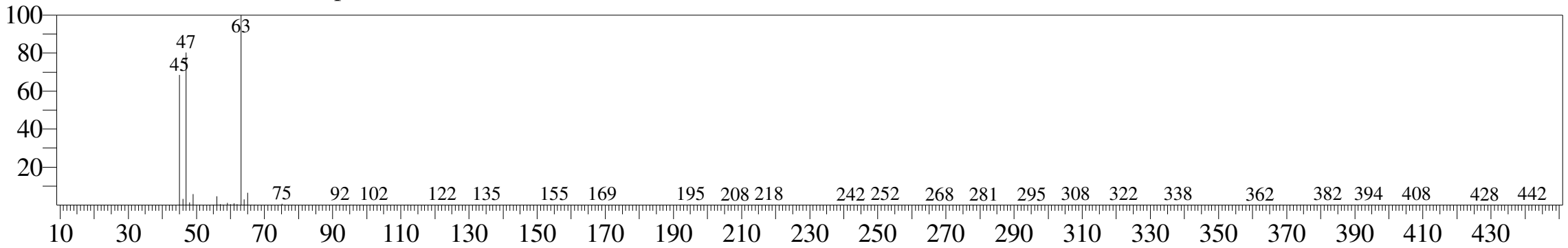

Hit#:1 Entry:11181 Library:NIST14.lib
SI:81 Formula:C3H7ClOS CAS:5331-57-7 MolWeight:126 RetIndex:1016
CompName:2-Chloroethyl methyl sulfoxide

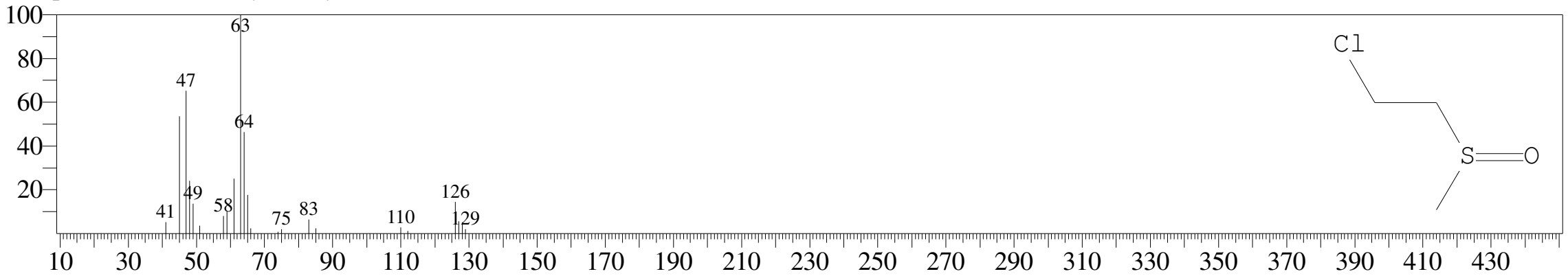

Hit#:2 Entry:33244 Library:NIST14.lib
SI:78 Formula:C6H10O5 CAS:1609-47-8 MolWeight:162 RetIndex:1028
CompName:Diethyl pyrocarbonate

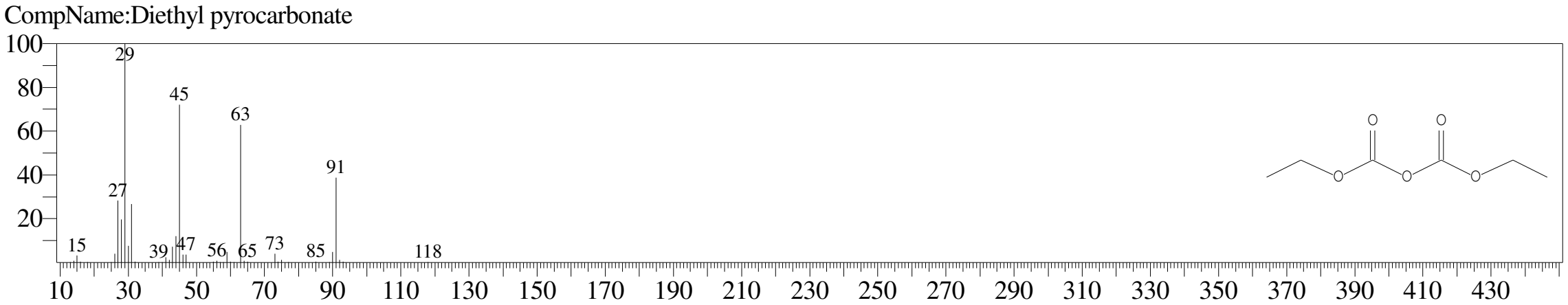

Hit#:3 Entry:1011 Library:NIST14.lib
SI:77 Formula:C2H6OS CAS:67-68-5 MolWeight:78 RetIndex:691
CompName:Dimethyl Sulfoxide

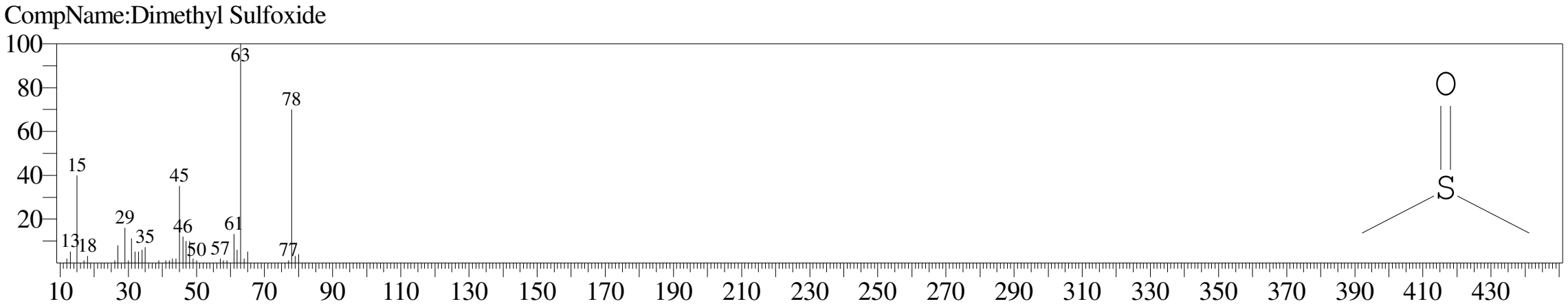

<< Target >>
Line#:4   R.Time:5.630(Scan#:1127) MassPeaks:250
RawMode:Averaged 5.625-5.635(1126-1128) BasePeak:47.00(92)
BG Mode:Calc. from Peak Group 1 - Event 1 Scan

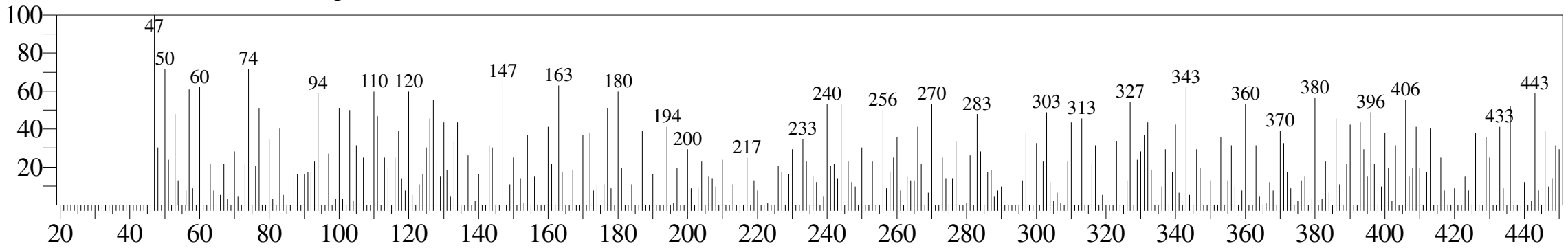

Hit#:1 Entry:57277 Library:NIST14.lib
SI:25 Formula:C7H12O6 CAS:77-95-2 MolWeight:192 RetIndex:1852
CompName:Quinic acid

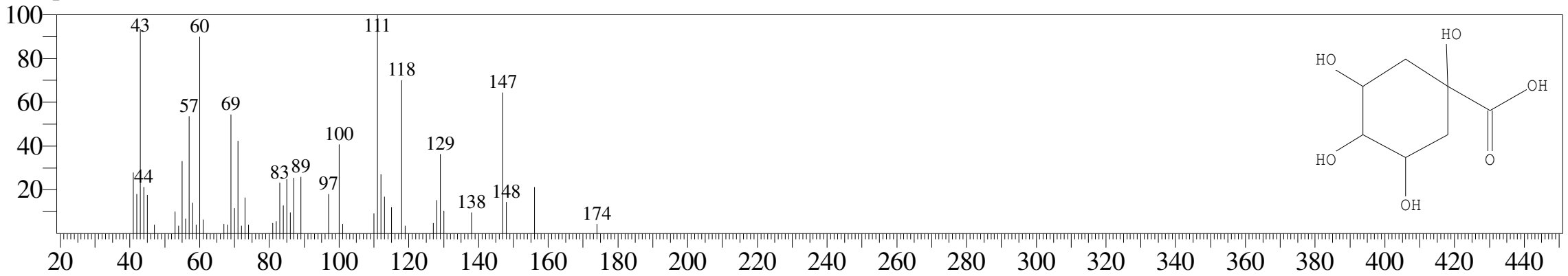

Hit#:2 Entry:142873 Library:NIST14.lib
SI:24 Formula:C16H13NO4 CAS:0-00-0 MolWeight:283 RetIndex:0
CompName:3-(2,3-Dihydro-1,4-benzodioxin-6-yl)-3-hydroxy-2H-isoindol-1-one

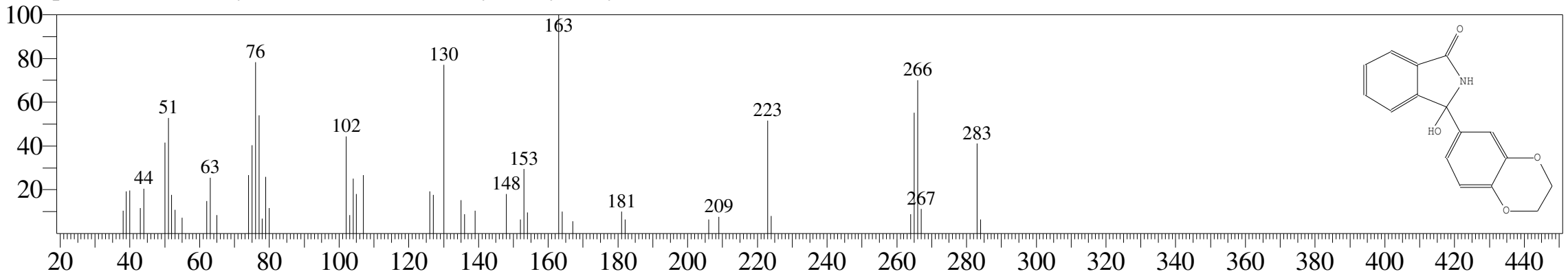

Hit#:3 Entry:272867 Library:NIST14.lib
SI:24 Formula:C27H30O15 CAS:57396-71-1 MolWeight:594 RetIndex:5384
CompName:4H-1-Benzopyran-4-one, 2-[4-[(6-deoxy-.alpha.-l-mannopyranosyl)oxy]phenyl]-8-(.beta.-d-glucopyranosyloxy)-5,7-dihydroxy-

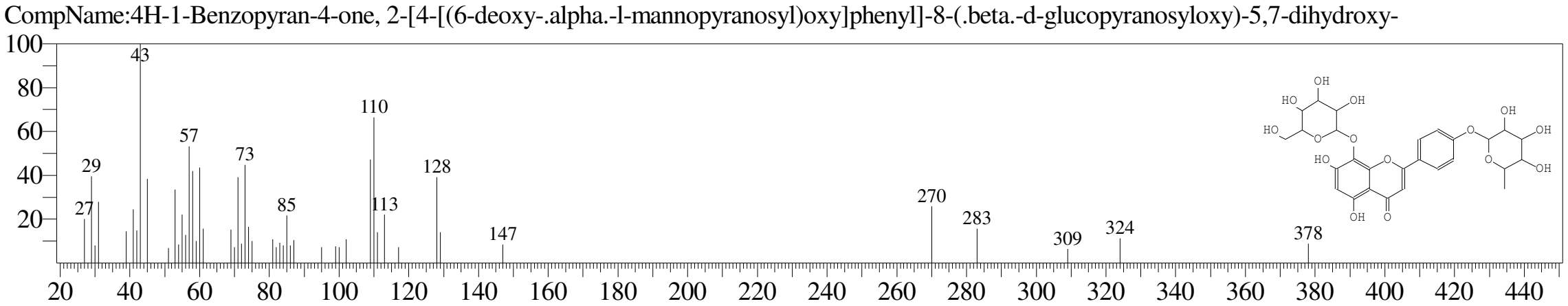

<< Target >>
Line#:5   R.Time:6.595(Scan#:1320) MassPeaks:232
RawMode:Averaged 6.590-6.600(1319-1321) BasePeak:60.00(71)
BG Mode:Calc. from Peak Group 1 - Event 1 Scan

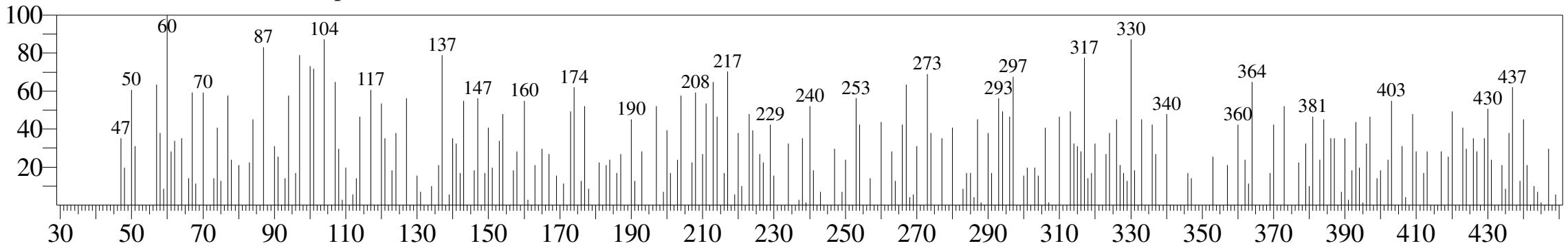

Hit#:1 Entry:175981 Library:NIST14.lib
SI:26 Formula:C18H27N3O2 CAS:114546-24-6 MolWeight:317 RetIndex:2335
CompName:Eseroline, N,N-diethylcarbamate(ester)

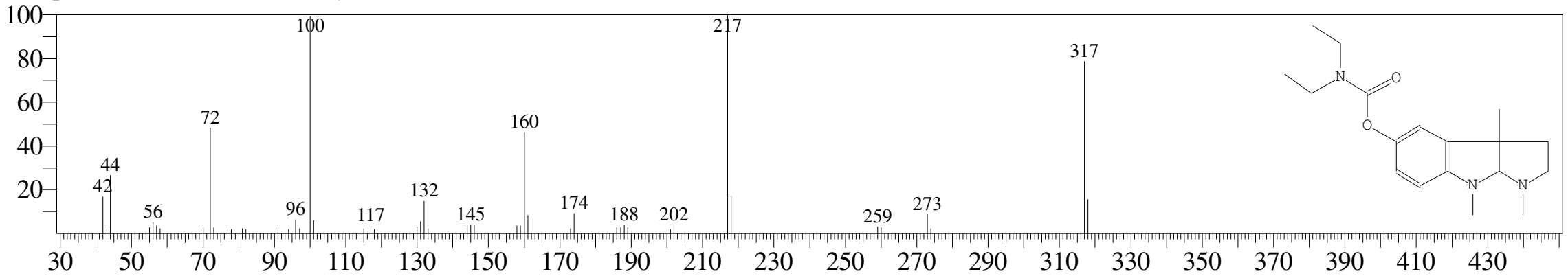

Hit#:2 Entry:188808 Library:NIST14.lib
SI:26 Formula:C15H13N3O4S CAS:36322-90-4 MolWeight:331 RetIndex:0
CompName:Piroxicam

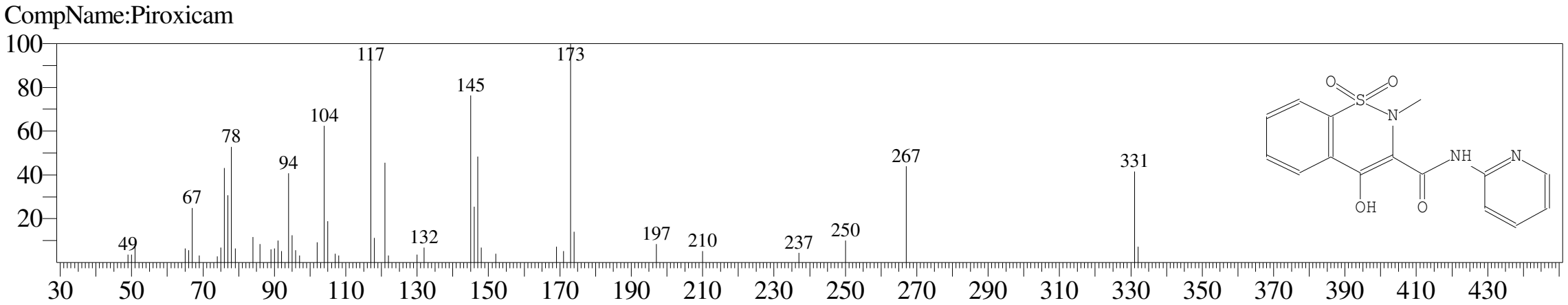

Hit#:3 Entry:188806 Library:NIST14.lib
SI:25 Formula:C15H13N3O4S CAS:36322-90-4 MolWeight:331 RetIndex:0
CompName:Piroxicam

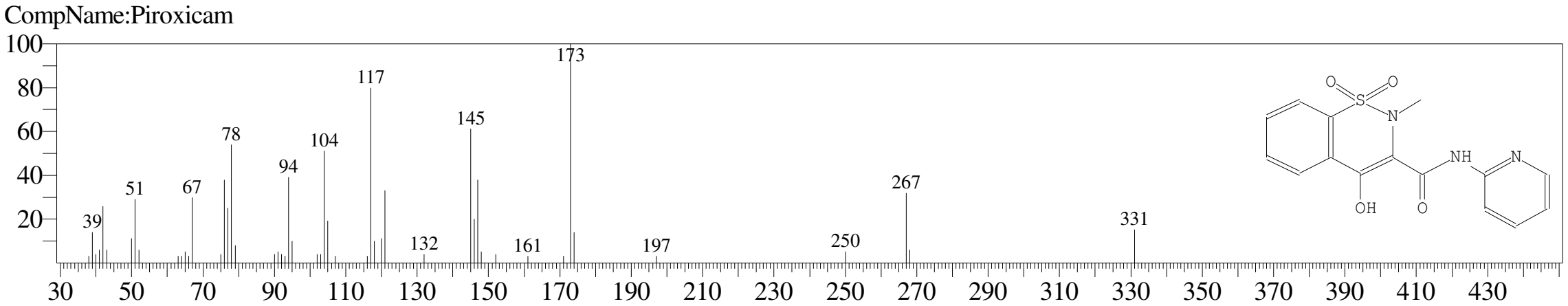

<< Target >>
Line#:6 R.Time:12.995(Scan#:2600) MassPeaks:222
RawMode:Averaged 12.990-13.000(2599-2601) BasePeak:159.00(84)
BG Mode:Calc. from Peak Group 1 - Event 1 Scan

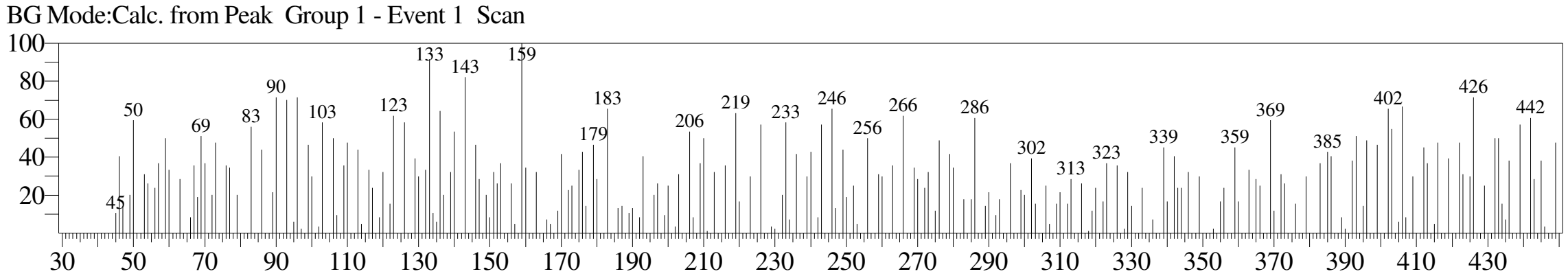

Hit#:1 Entry:219225 Library:NIST14.lib
SI:24 Formula:C22H44O2Si CAS:0-00-0 MolWeight:368 RetIndex:0
CompName:2-methylidene-6,10,14-trimethylpen2-methylidene-6,10,14-trimethylpentadecanoic acid silylated

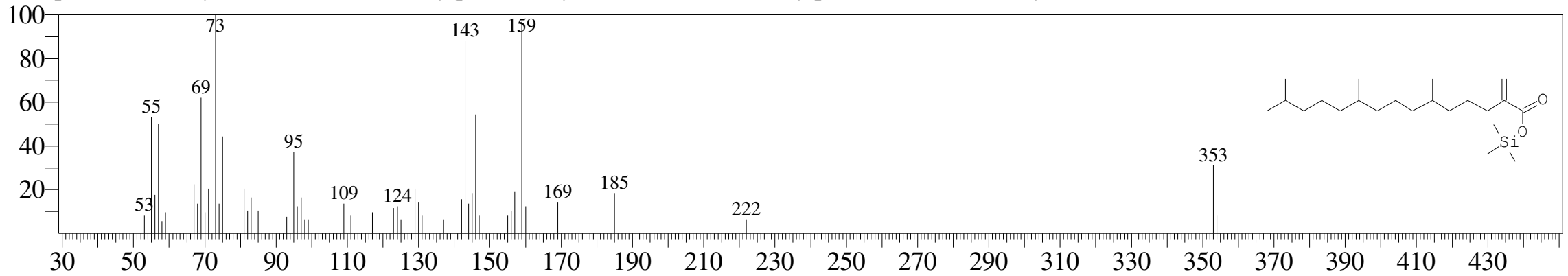

Hit#:2 Entry:192466 Library:NIST14.lib
SI:23 Formula:C10H7F6NO3S CAS:0-00-0 MolWeight:335 RetIndex:1443
CompName:Propionic acid, 3,3,3-trifluoro-2-thiophen-2-yl-2-(2,2,2-trifluoroacetylamino)-, methyl ester

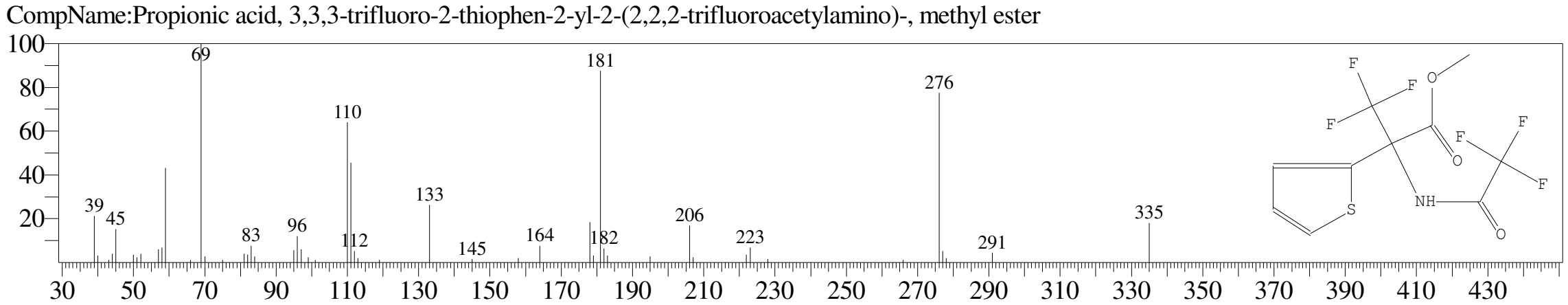

Hit#:3 Entry:160292 Library:NIST14.lib
SI:23 Formula:C13H17Cl2NOSi CAS:0-00-0 MolWeight:301 RetIndex:1981
CompName:2,3-Dichlorobenzyl alcohol, (3-cyanopropyl)dimethylsilyl ether

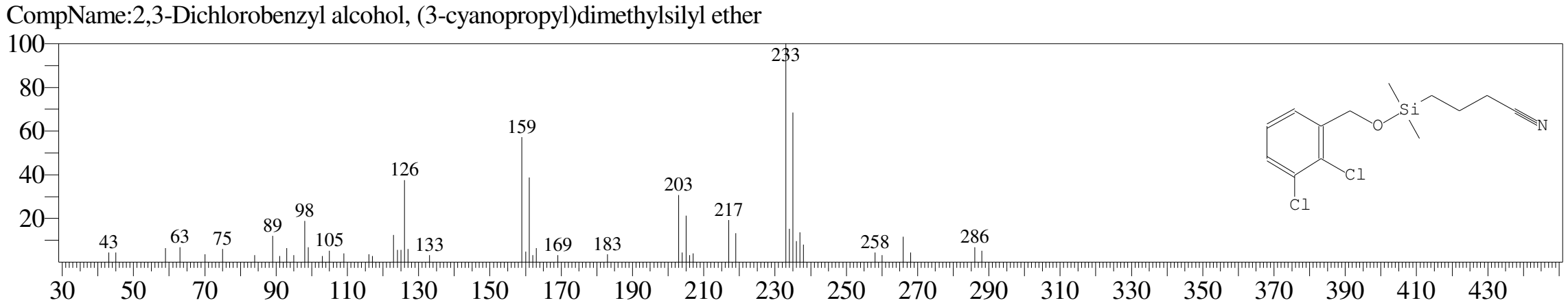

<< Target >>
Line#:7 R.Time:13.890(Scan#:2779) MassPeaks:232
RawMode:Averaged 13.885-13.895(2778-2780) BasePeak:61.00(64)
BG Mode:Calc. from Peak Group 1 - Event 1 Scan

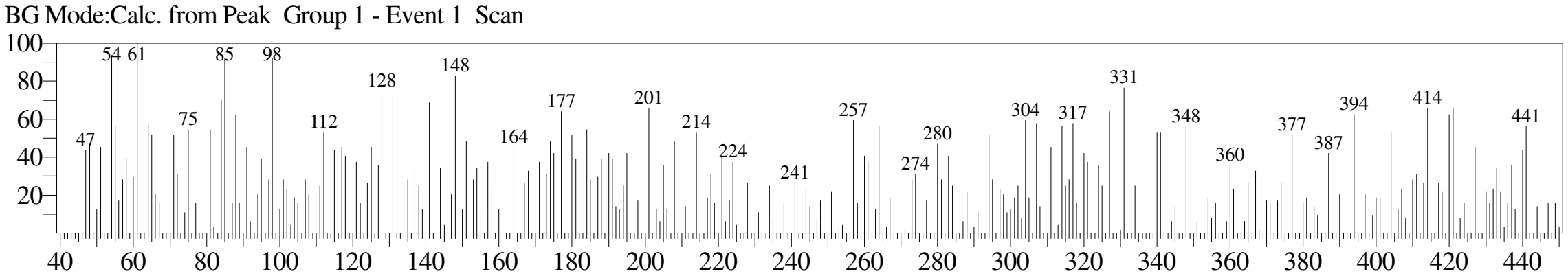

Hit#:1 Entry:168376 Library:NIST14.lib
SI:27 Formula:C11H22N2O4S2 CAS:0-00-0 MolWeight:310 RetIndex:2541
CompName:Thiosulfuric acid S-2-[[4-[[-1-pyrrolidinyl]carbonyl]butyl]amino]ethyl ester

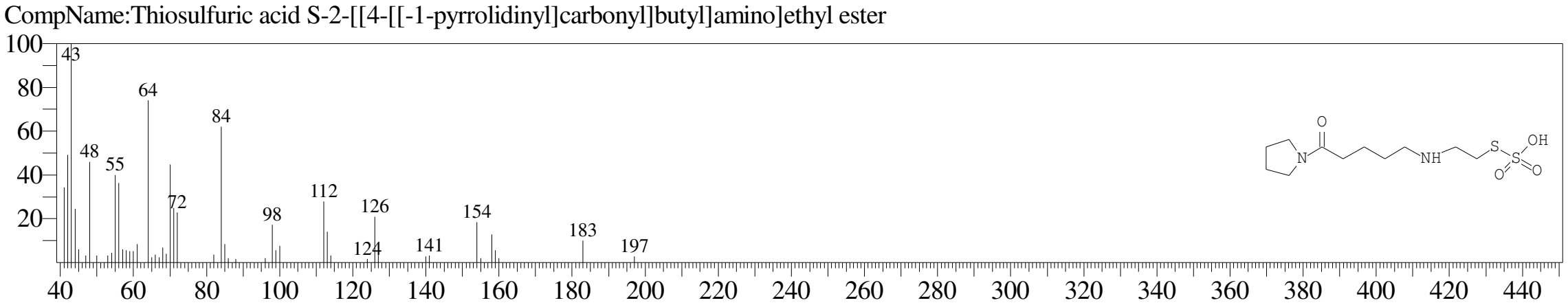

Hit#:2 Entry:263009 Library:NIST14.lib
SI:26 Formula:C18H29N3O10S CAS:21026-92-6 MolWeight:479 RetIndex:3601
CompName:Glutamine, N2-carboxy-N-[1-[(carboxymethyl)carbamoyl]-2-mercaptoethyl]-, N2-ethyl dimethyl ester, ethyl carbamate (ester), L-

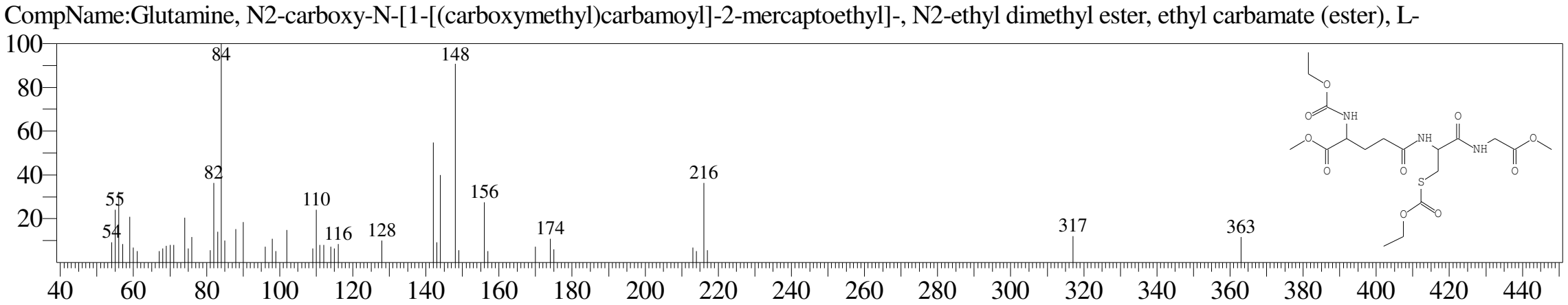

Hit#:3 Entry:175741 Library:NIST14.lib
SI:26 Formula:C15H24ClNS2 CAS:0-00-0 MolWeight:317 RetIndex:2309
CompName:1-[4-Chlorophenyl]-2-methyl-2-isopentylamino-1-propyl hydrodisulfide

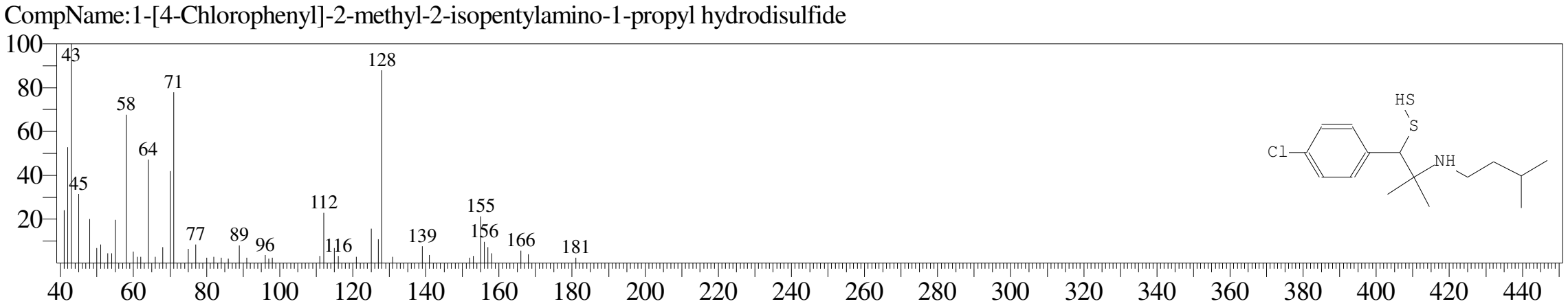

<< Target >>
Line#:8 R.Time:15.580(Scan#:3117) MassPeaks:218
RawMode:Averaged 15.575-15.585(3116-3118) BasePeak:46.00(71)
BG Mode:Calc. from Peak Group 1 - Event 1 Scan

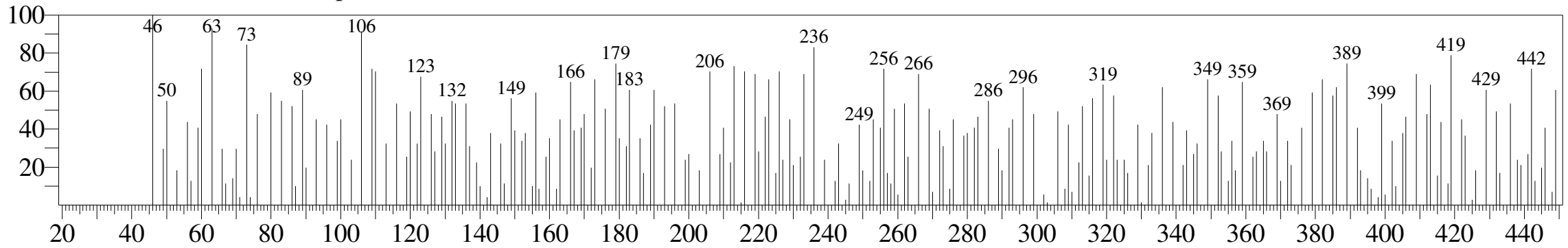

Hit#:1 Entry:97785 Library:NIST14.lib
SI:23 Formula:C6H5ClN2O4S CAS:97-09-6 MolWeight:236 RetIndex:2019
CompName:Benzenesulfonamide, 4-chloro-3-nitro-

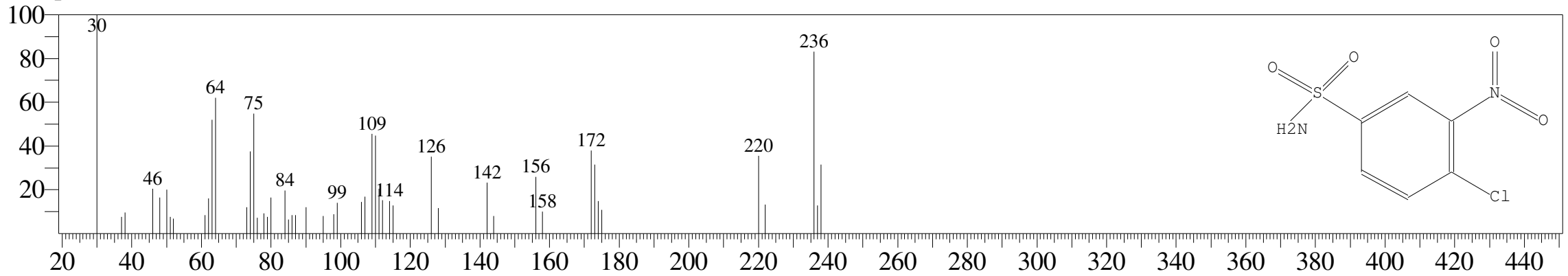

Hit#:2 Entry:188213 Library:NIST14.lib
SI:22 Formula:C19H19FO4 CAS:0-00-0 MolWeight:330 RetIndex:2322
CompName:Phthalic acid, 3-fluorobenzyl isobutyl ester

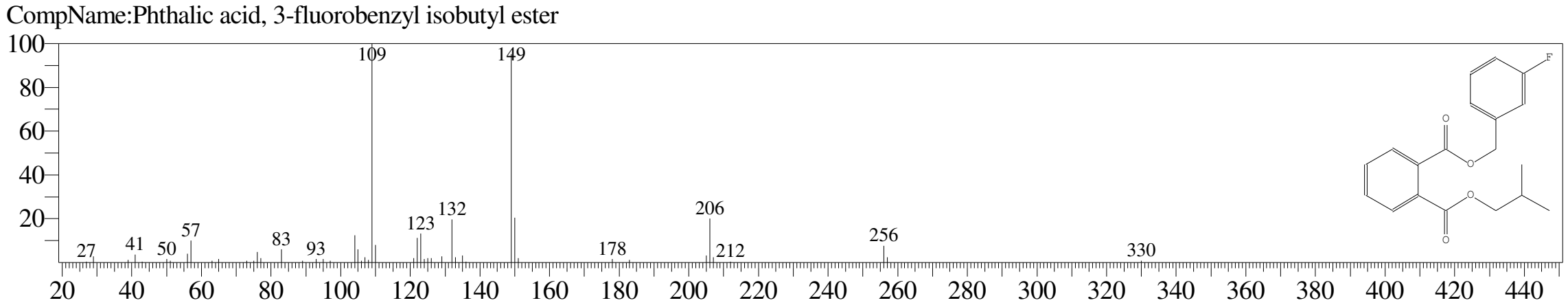

Hit#:3 Entry:188225 Library:NIST14.lib
SI:22 Formula:C19H19FO4 CAS:0-00-0 MolWeight:330 RetIndex:2322
CompName:Phthalic acid, 2-fluorobenzyl isobutyl ester

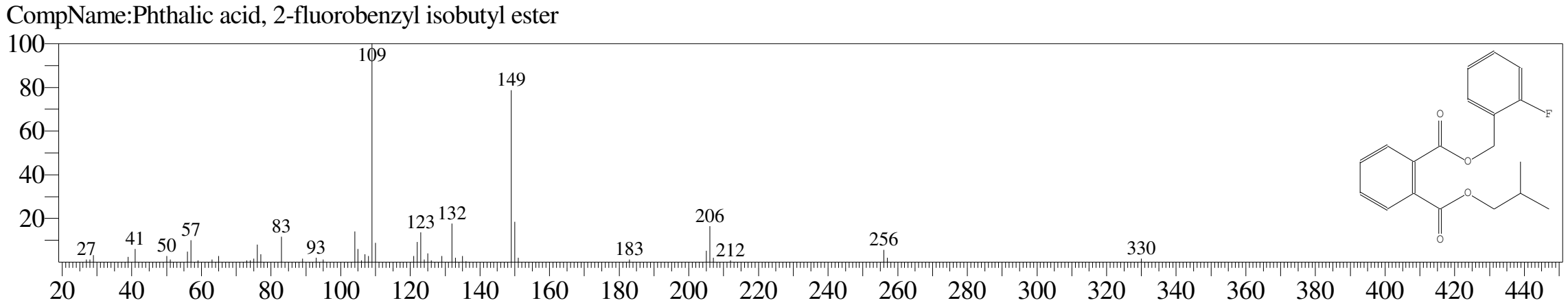

<< Target >>
Line#:9   R.Time:16.295(Scan#:3260) MassPeaks:237
RawMode:Averaged 16.290-16.300(3259-3261) BasePeak:192.00(65)
BG Mode:Calc. from Peak Group 1 - Event 1 Scan

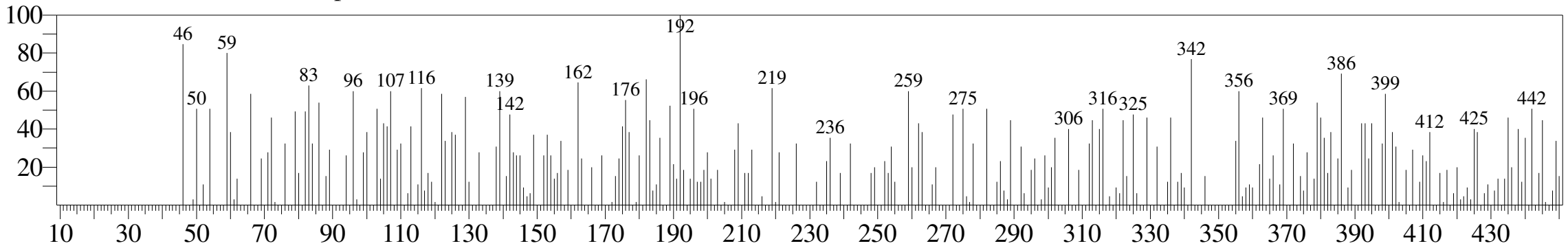

Hit#:1 Entry:50020 Library:NIST14.lib
SI:25 Formula:C11H18O2 CAS:88653-52-5 MolWeight:182 RetIndex:1270
CompName:Bicyclo[2.2.1]heptan-2-one, 4-methoxy-1,7,7-trimethyl-

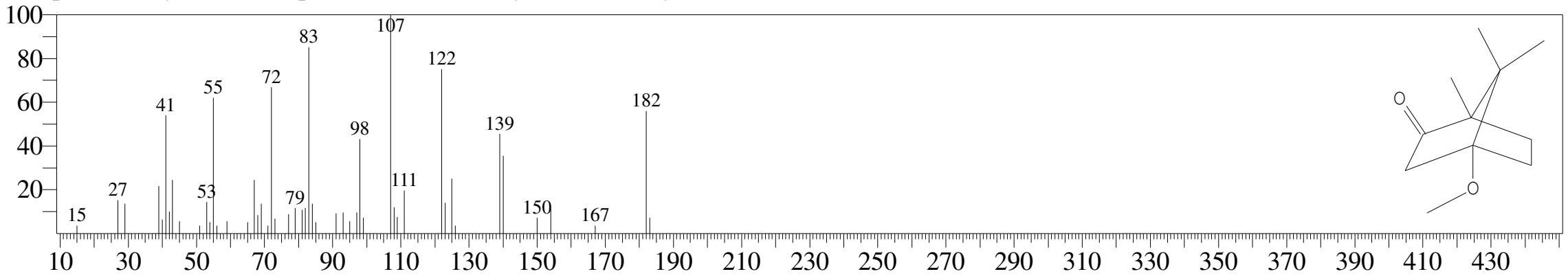

Hit#:2 Entry:102113 Library:NIST14.lib
SI:24 Formula:C13H20O4 CAS:0-00-0 MolWeight:240 RetIndex:1608
CompName:Cyclopentanecarboxylic acid, 2-(2,2-dimethyl-1,3-dioxolane-4-yl)-4-methylene-, methyl ester, cis-S,S,R-

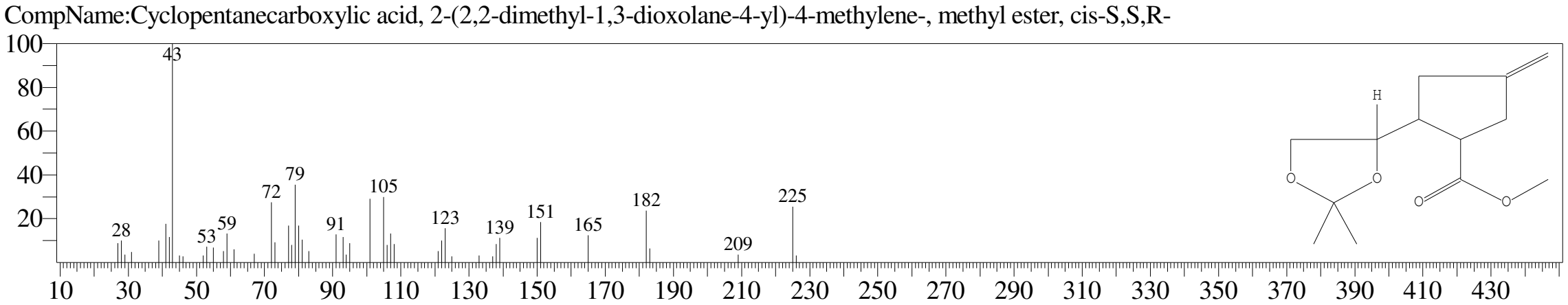

Hit#:3 Entry:28295 Library:NIST14.lib
SI:24 Formula:C10H18O CAS:38092-33-0 MolWeight:154 RetIndex:1100
CompName:trans-2,7-Dimethyl-3,6-octadien-2-ol

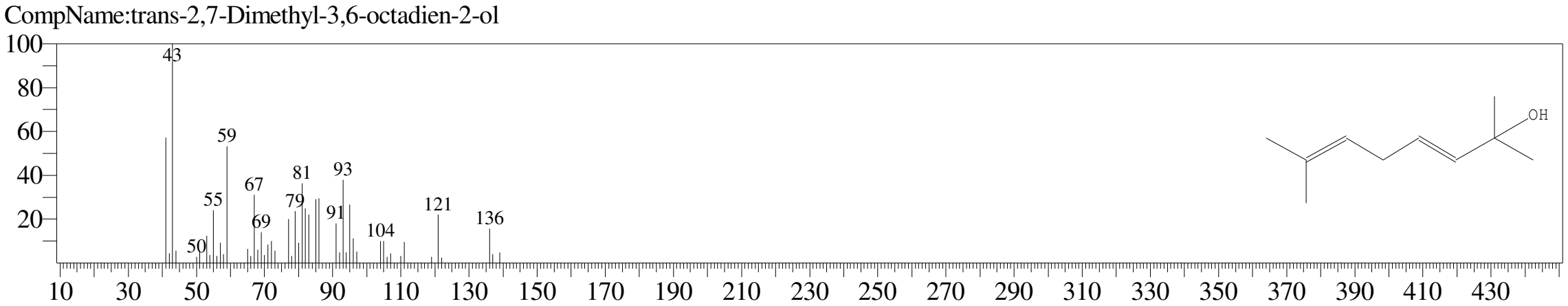

<< Target >>
Line#:10 R.Time:16.995(Scan#:3400) MassPeaks:230
RawMode:Averaged 16.990-17.000(3399-3401) BasePeak:46.00(113)
BG Mode:Calc. from Peak Group 1 - Event 1 Scan

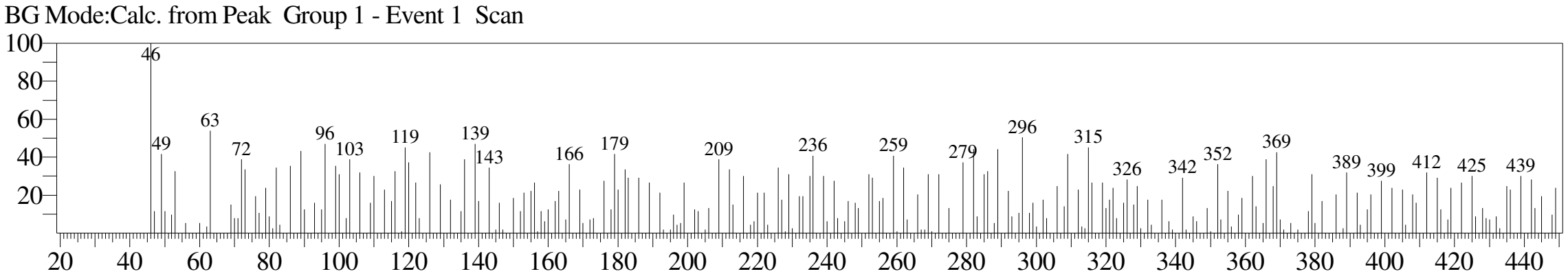

Hit#:1 Entry:39074 Library:NIST14.lib
SI:26 Formula:C5H7N5S CAS:0-00-0 MolWeight:169 RetIndex:0
CompName:Acetonitrile, 2-[(1-ethyl-1H-1,2,3,4-tetrazol-5-yl)thio]-

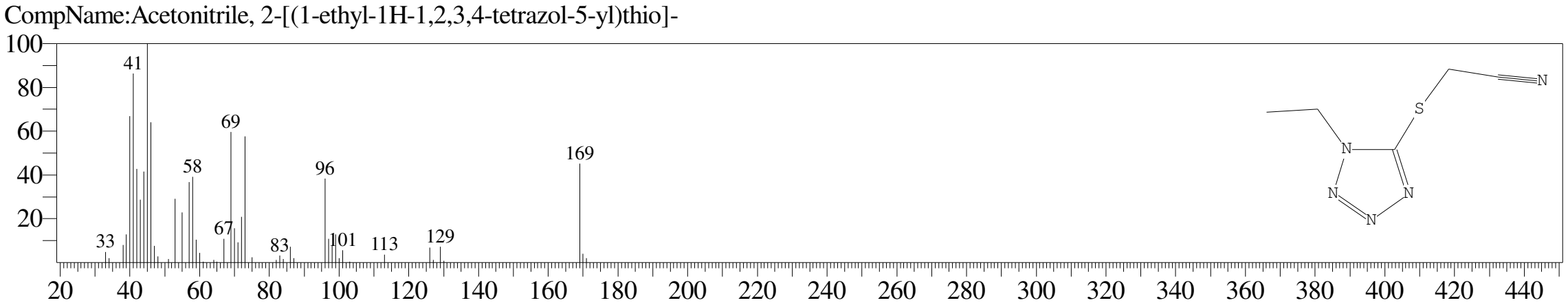

Hit#:2 Entry:49310 Library:NIST14.lib
SI:25 Formula:C7H3ClN2O2 CAS:6575-07-1 MolWeight:182 RetIndex:1534
CompName:Benzonitrile, 2-chloro-6-nitro-

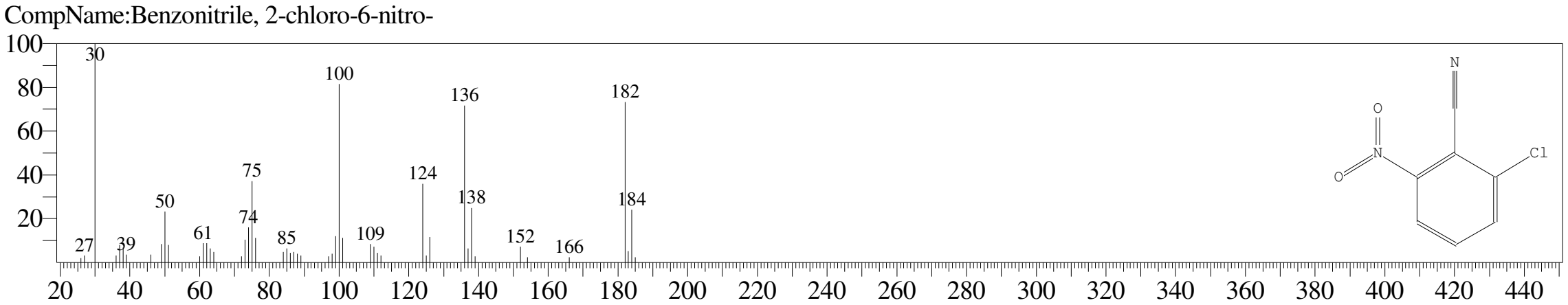

Hit#:3 Entry:49306 Library:NIST14.lib
SI:25 Formula:C7H3ClN2O2 CAS:939-80-0 MolWeight:182 RetIndex:1534
CompName:4-Chloro-3-nitrobenzonitrile

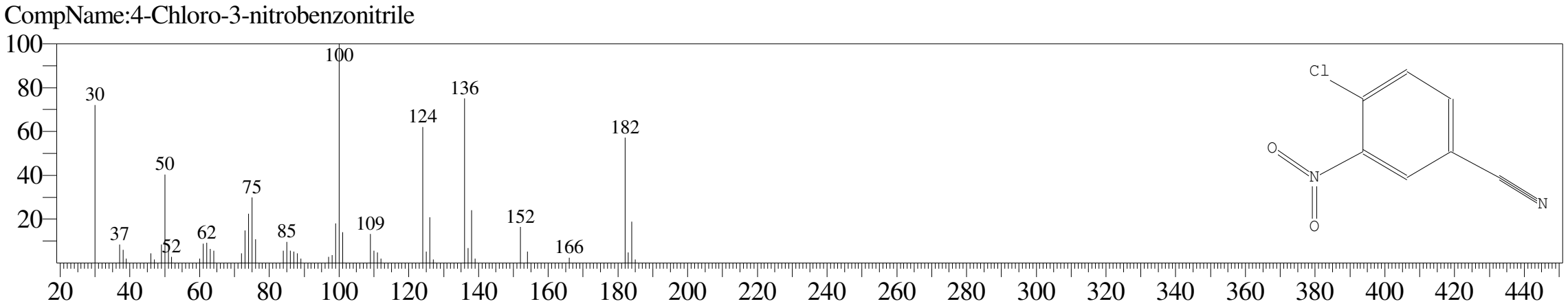

<< Target >>
Line#:11   R.Time:17.170(Scan#:3435) MassPeaks:238
RawMode:Averaged 17.165-17.175(3434-3436) BasePeak:57.00(67)
BG Mode:Calc. from Peak Group 1 - Event 1 Scan

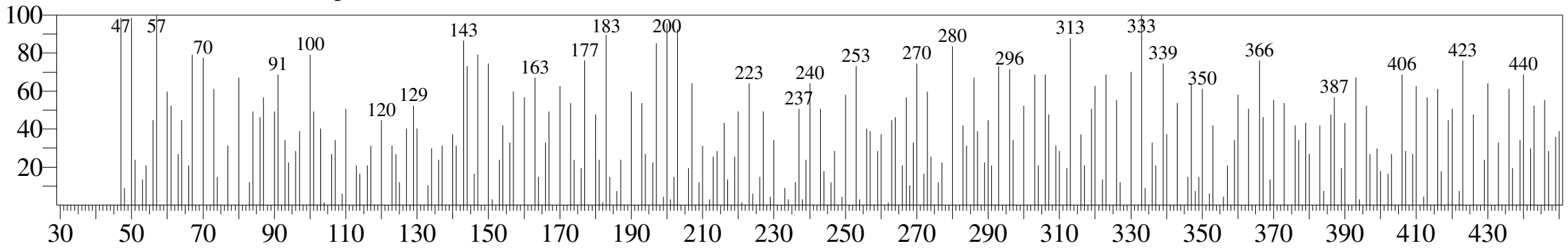

Hit#:1 Entry:222983 Library:NIST14.lib
SI:22 Formula:C23H35NO3 CAS:17916-30-2 MolWeight:373 RetIndex:2600
CompName:Pregn-5-en-20-one, 3-(acetyloxy)-, 20-oxime, (3.beta.)-

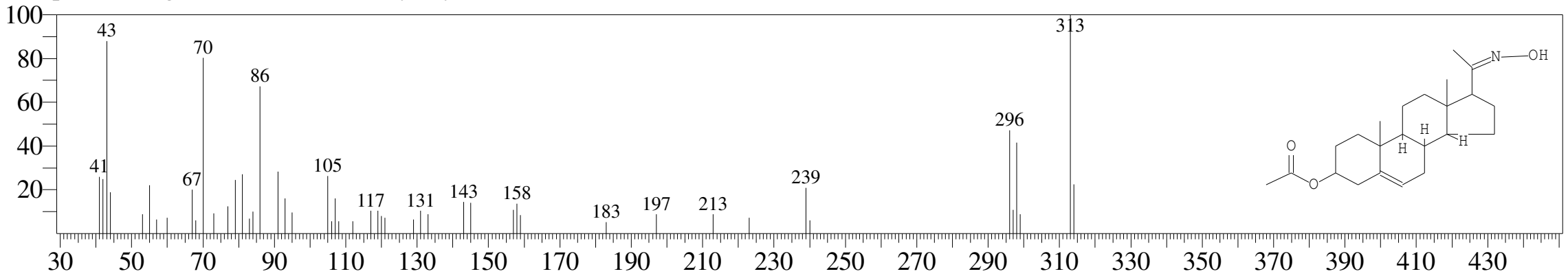

Hit#:2 Entry:269410 Library:NIST14.lib
SI:21 Formula:C30H42O8 CAS:466-06-8 MolWeight:530 RetIndex:4205
CompName:Proscillaridin

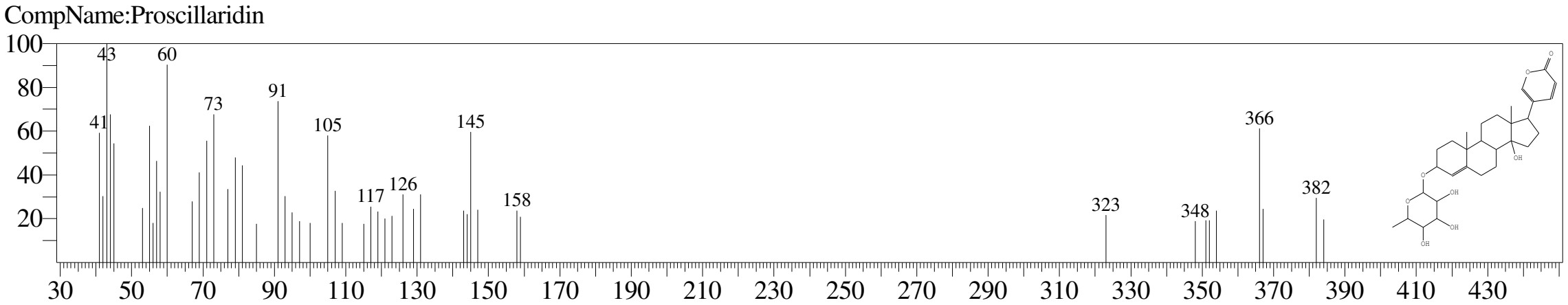

Hit#:3 Entry:207809 Library:NIST14.lib
SI:21 Formula:C19H19N3O4 CAS:296879-75-9 MolWeight:353 RetIndex:3000
CompName:Imidazolidine, 1-(4-nitrophenylbenzoyl)-3-propinoyl-2-phenyl-

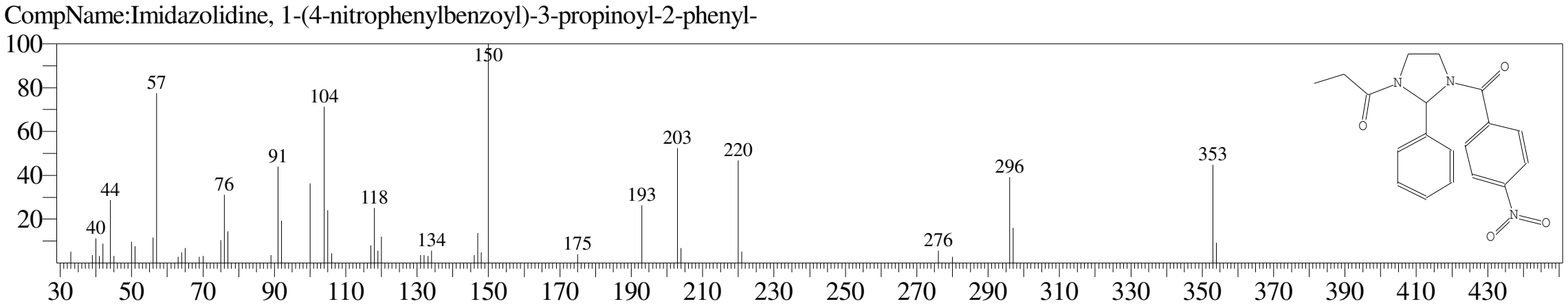

<< Target >>
Line#:12 R.Time:19.200(Scan#:3841) MassPeaks:221
RawMode:Averaged 19.195-19.205(3840-3842) BasePeak:236.00(69)
BG Mode:Calc. from Peak Group 1 - Event 1 Scan

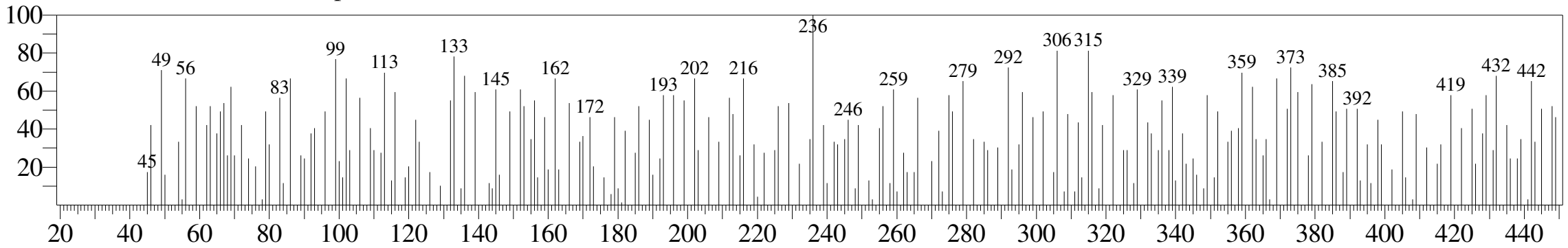

Hit#:1 Entry:258629 Library:NIST14.lib
SI:23 Formula:C18H26F8O4 CAS:0-00-0 MolWeight:458 RetIndex:1526
CompName:Diethylmalonic acid, hexyl 2,2,3,3,4,4,5,5-octafluoropentyl ester

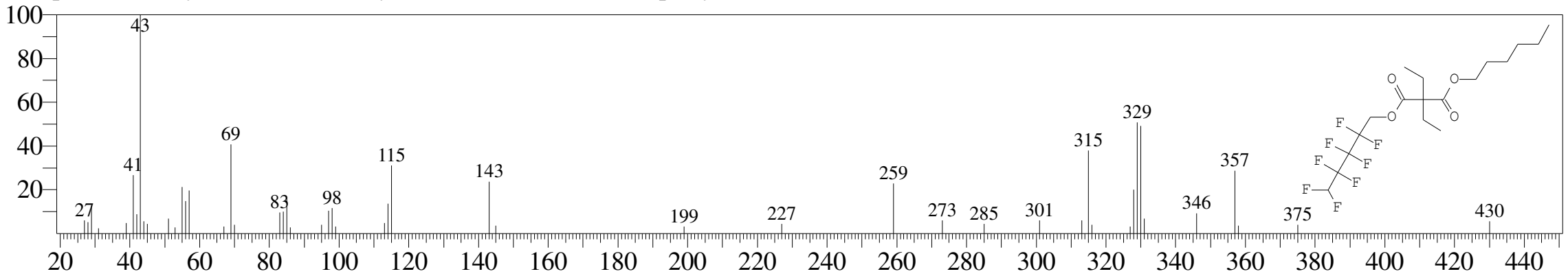

Hit#:2 Entry:254963 Library:NIST14.lib
SI:21 Formula:C17H24F8O4 CAS:0-00-0 MolWeight:444 RetIndex:1427
CompName:Diethylmalonic acid, pentyl 2,2,3,3,4,4,5,5-octafluoropentyl ester

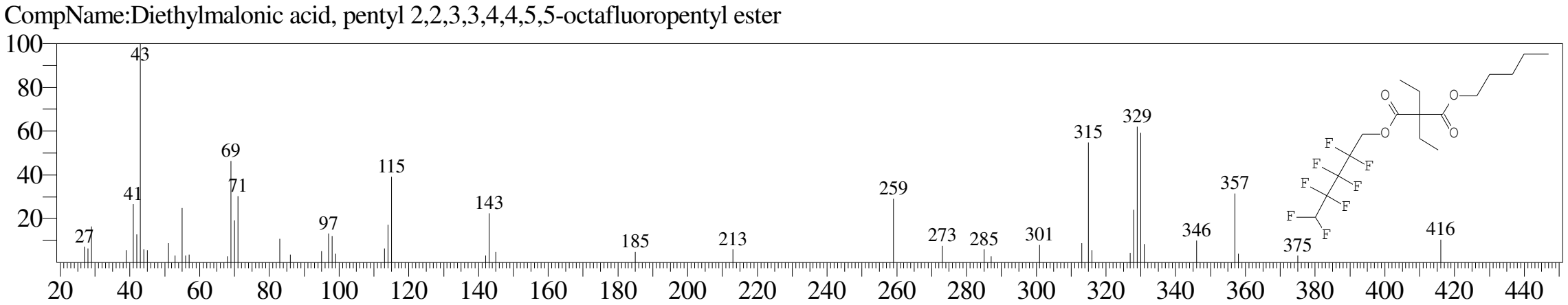

Hit#:3 Entry:260640 Library:NIST14.lib
SI:21 Formula:C16H13F8NO6 CAS:0-00-0 MolWeight:467 RetIndex:0
CompName:Succinic acid, 2,2,3,3,4,4,5,5-octafluoropentyl 3-nitrobenzyl ester

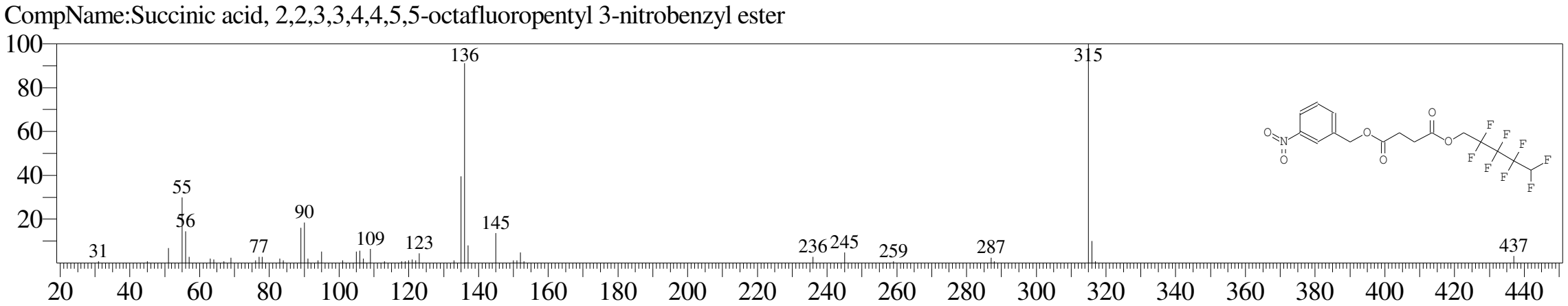

<< Target >>
Line#:13   R.Time:22.820(Scan#:4565) MassPeaks:244
RawMode:Averaged 22.815-22.825(4564-4566) BasePeak:46.00(94)
BG Mode:Calc. from Peak Group 1 - Event 1 Scan

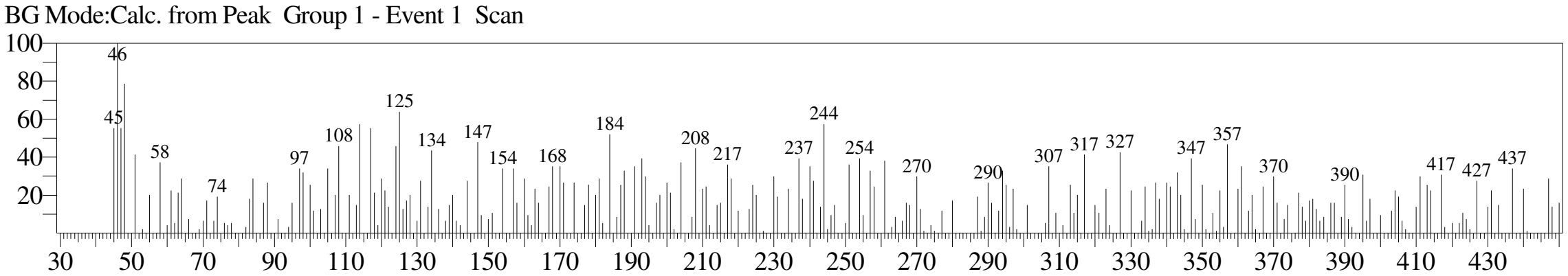

Hit#:1 Entry:89460 Library:NIST14.lib
SI:28 Formula:C12H19FN2O CAS:0-00-0 MolWeight:226 RetIndex:1582
CompName:Acetamide, N-methyl-N-[4-[4-fluoro-1-hexahydropyridyl]-2-butynyl]-

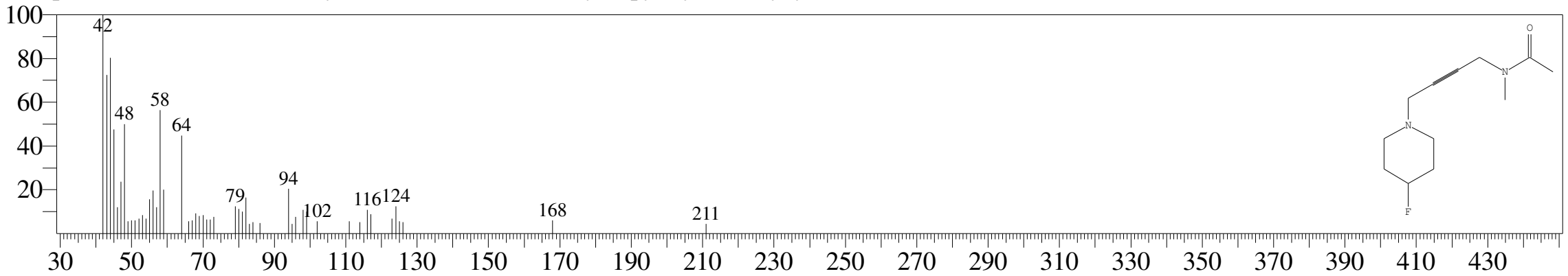

Hit#:2 Entry:175799 Library:NIST14.lib
SI:27 Formula:C16H19N3O2S CAS:0-00-0 MolWeight:317 RetIndex:2712
CompName:1-(5-tert-Butyl-thiophene-2-carbonyl)-3-(3-methyl-pyridin-2-yl)-urea

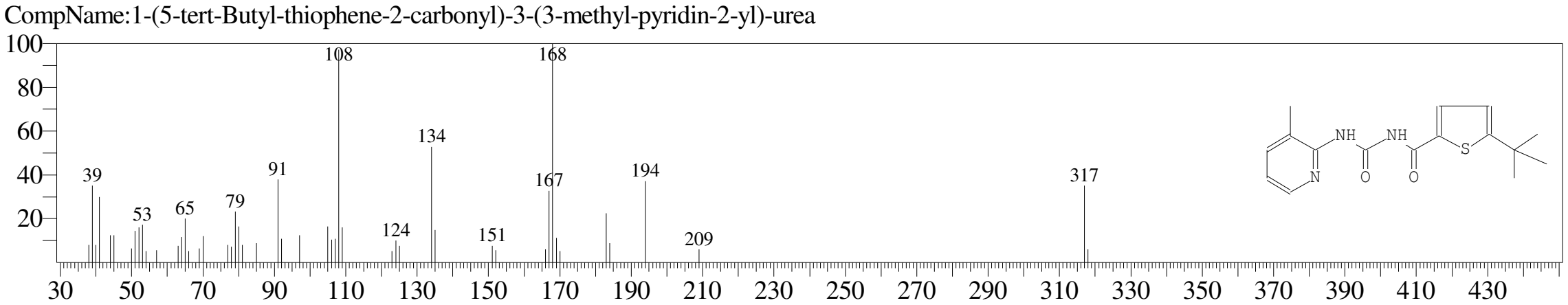

Hit#:3 Entry:153204 Library:NIST14.lib
SI:26 Formula:C14H10N6S CAS:0-00-0 MolWeight:294 RetIndex:2577
CompName:7H-[1,2,4]Triazolo[3,4-b][1,3,4]thiadiazine, 6-phenyl-3-(2-pyrazinyl)-

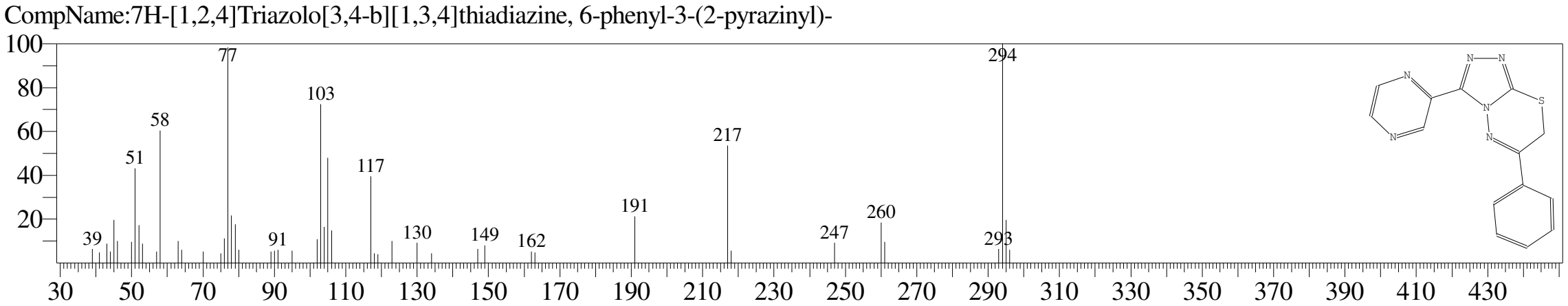

<< Target >>
Line#:14   R.Time:24.505(Scan#:4902) MassPeaks:205
RawMode:Averaged 24.500-24.510(4901-4903) BasePeak:207.00(69)
BG Mode:Calc. from Peak Group 1 - Event 1 Scan

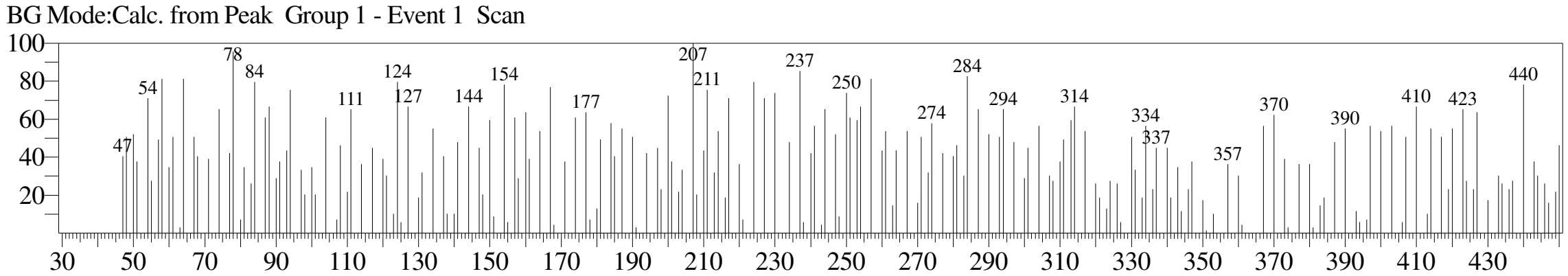

Hit#:1 Entry:253349 Library:NIST14.lib
SI:25 Formula:C28H42O2Si CAS:0-00-0 MolWeight:438 RetIndex:2940
CompName:Silane, diphenylcitronellyloxyhexyloxy-

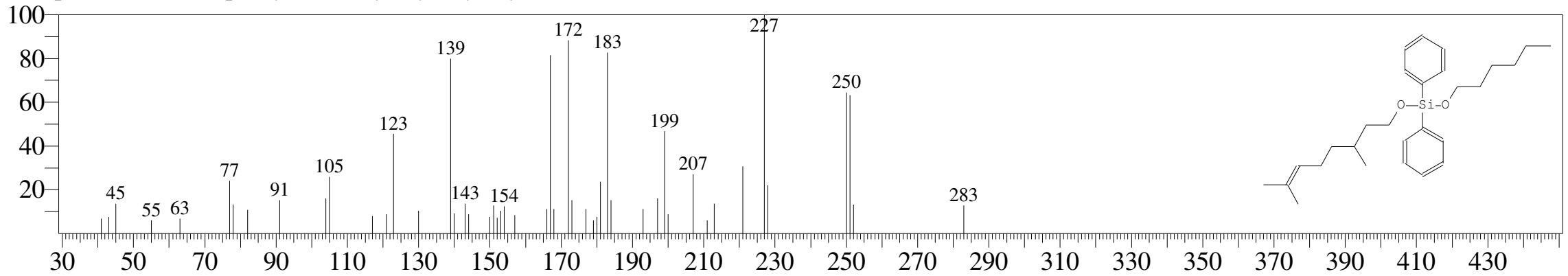

Hit#:2 Entry:253343 Library:NIST14.lib
SI:25 Formula:C28H42O2Si CAS:0-00-0 MolWeight:438 RetIndex:2876
CompName:Silane, diphenylcitronellyloxyisohexyloxy-

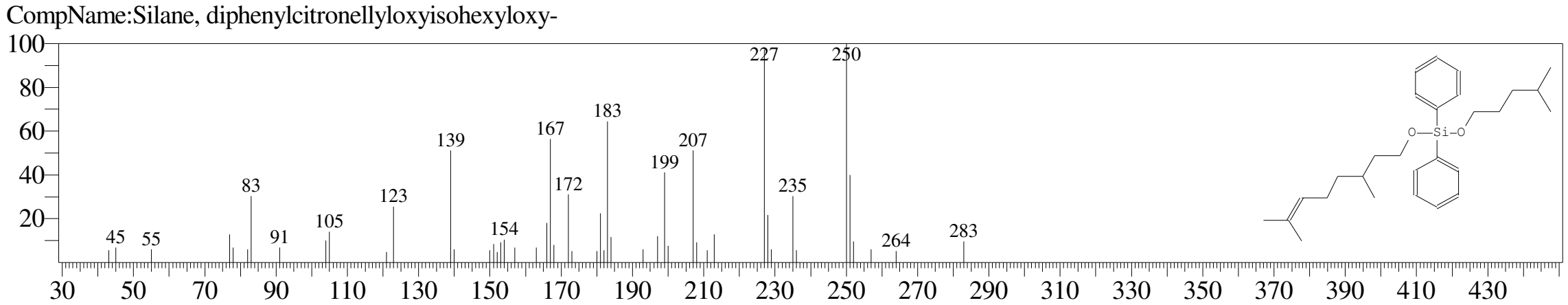

Hit#:3 Entry:112039 Library:NIST14.lib
SI:24 Formula:C10H10BrN3 CAS:0-00-0 MolWeight:251 RetIndex:0
CompName:3-(3-Bromophenyl)-4-ethyl-4H-1,2,4-triazole

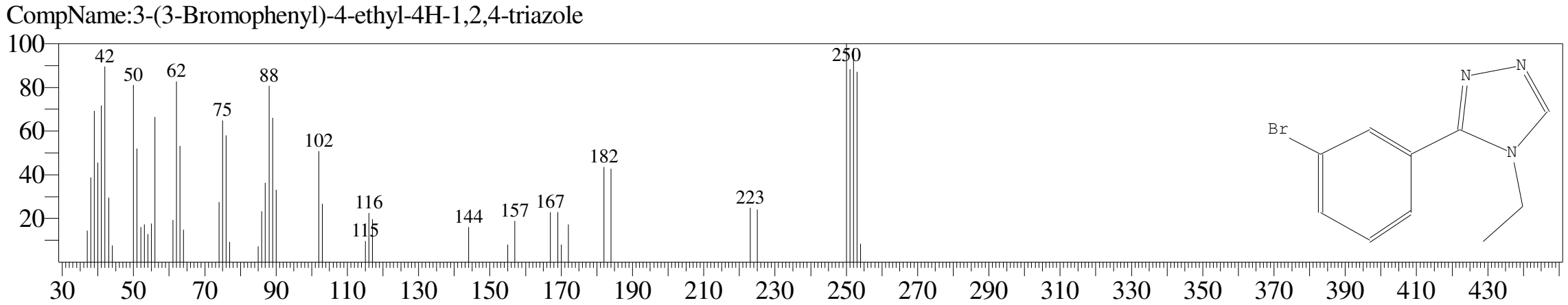

<< Target >>
Line#:15 R.Time:24.690(Scan#:4939) MassPeaks:256
RawMode:Averaged 24.685-24.695(4938-4940) BasePeak:296.00(64)
BG Mode:Calc. from Peak Group 1 - Event 1 Scan

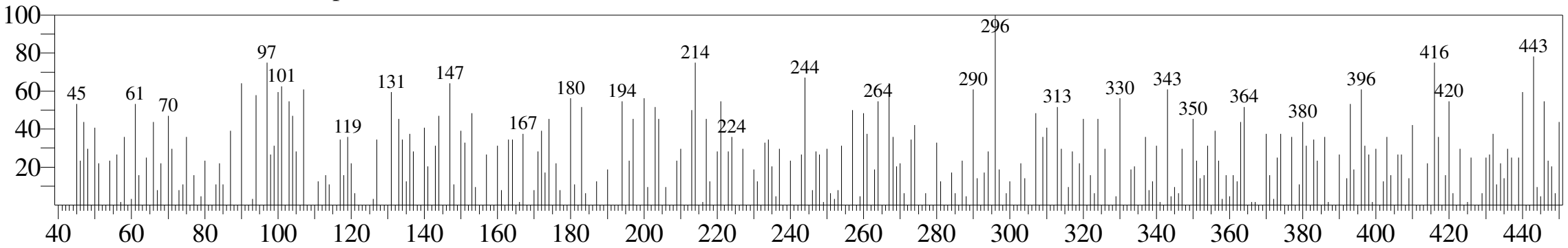

Hit#:1 Entry:105422 Library:NIST14.lib
SI:25 Formula:C9H7F3N4O CAS:32706-08-4 MolWeight:244 RetIndex:1443
CompName:Pteridine, 2-ethoxy-4-(trifluoromethyl)-

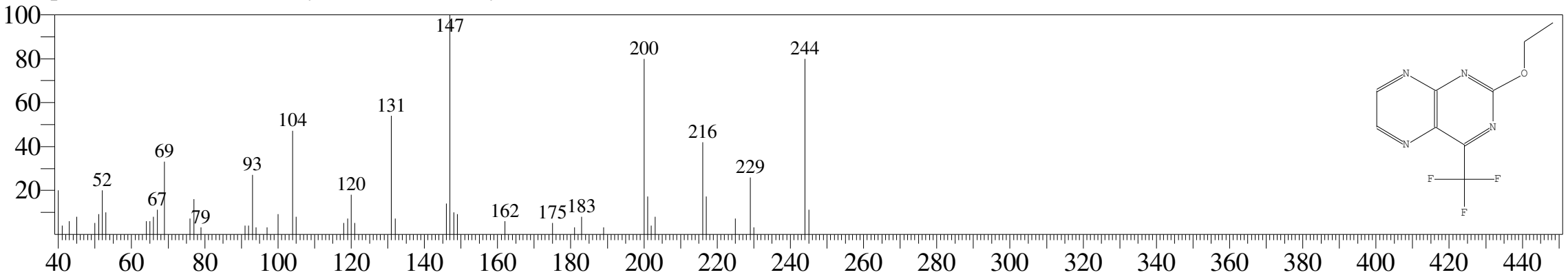

Hit#:2 Entry:244626 Library:NIST14.lib
SI:22 Formula:C19H34N4O6 CAS:33861-49-3 MolWeight:414 RetIndex:3550
CompName:Hexanoic acid, 3-(acetylamino)-6-[[3,6-bis(acetylamino)-1-oxohexyl]amino]-, methyl ester, [S-(R*,R*)]-

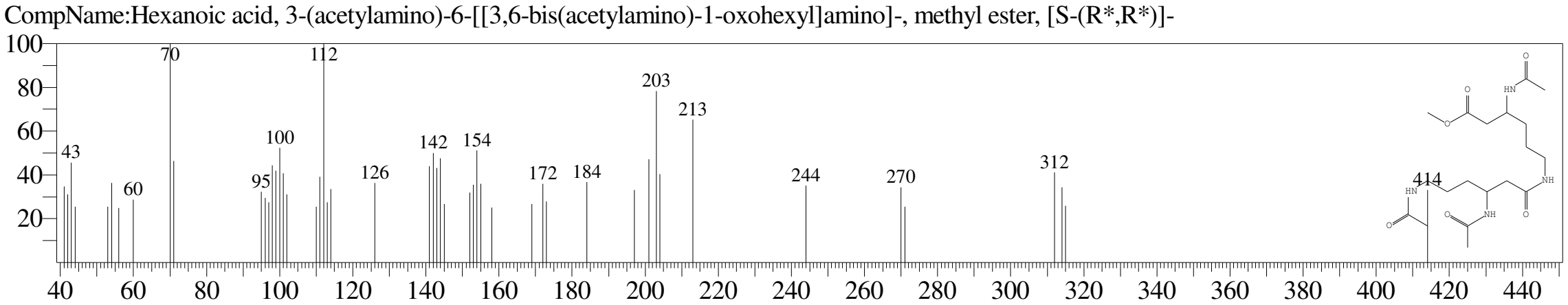

Hit#:3 Entry:267787 Library:NIST14.lib
SI:22 Formula:C20H52O5Si5 CAS:14199-72-5 MolWeight:512 RetIndex:1746
CompName:Xylitol, 5TMS derivative

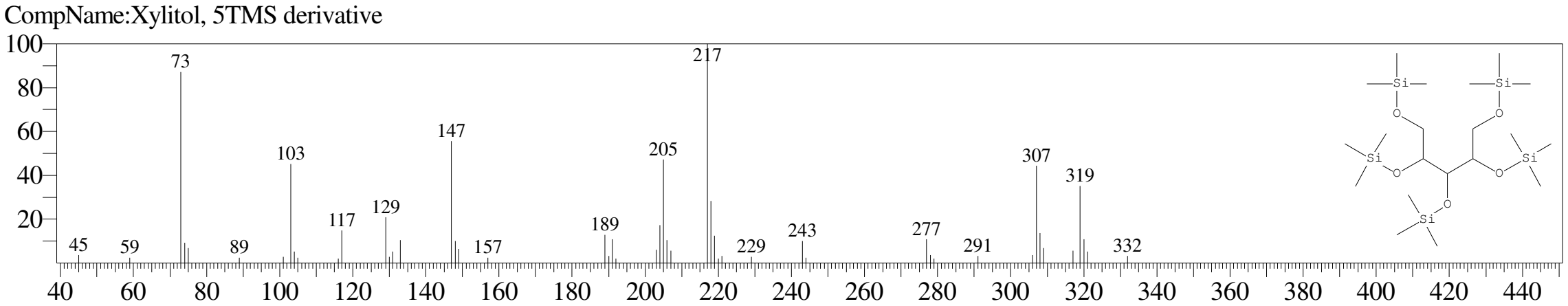

<< Target >>
Line#:16 R.Time:25.005(Scan#:5002) MassPeaks:228
RawMode:Averaged 25.000-25.010(5001-5003) BasePeak:109.00(74)
BG Mode:Calc. from Peak Group 1 - Event 1 Scan

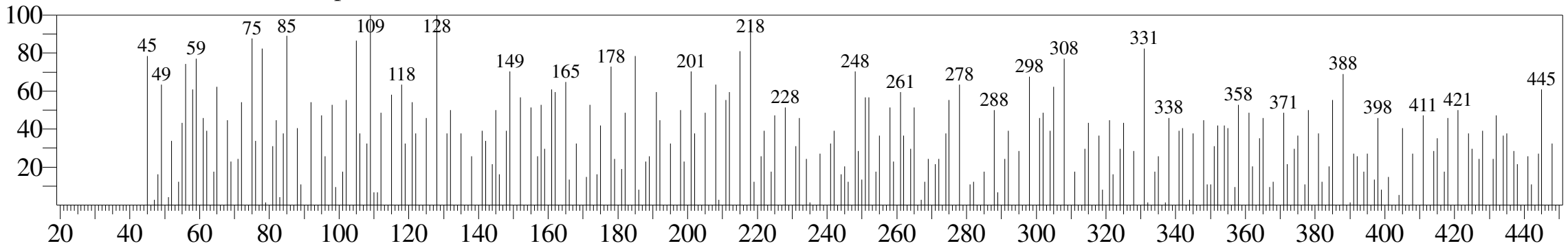

Hit#:1 Entry:91702 Library:NIST14.lib
SI:29 Formula:C14H16OSi CAS:170954-82-2 MolWeight:228 RetIndex:1619
CompName:1-Ethenyl(dimethyl)silyloxynaphthalene

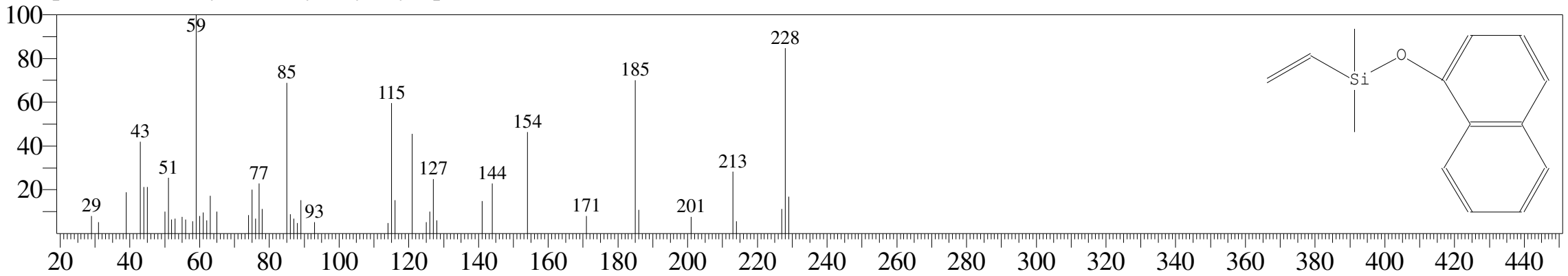

Hit#:2 Entry:109704 Library:NIST14.lib
SI:27 Formula:C14H16S2 CAS:42506-61-6 MolWeight:248 RetIndex:1949
CompName:Bithiopyranylidene, 2,2',6,6'-tetramethyl-

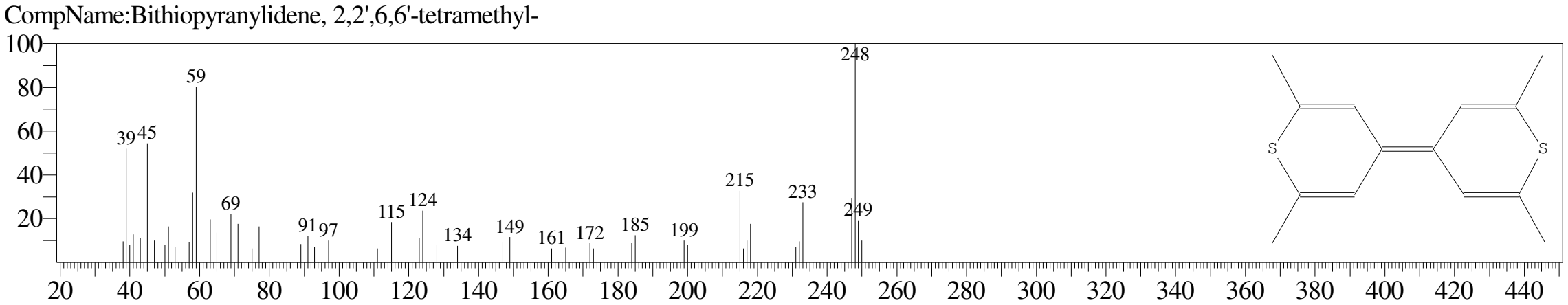

Hit#:3 Entry:147127 Library:NIST14.lib
SI:27 Formula:C16H33NO3 CAS:77611-57-5 MolWeight:287 RetIndex:1964
CompName:Acetamide, 2-[2-(hexyloxy)ethoxy]-N,N-dipropyl-

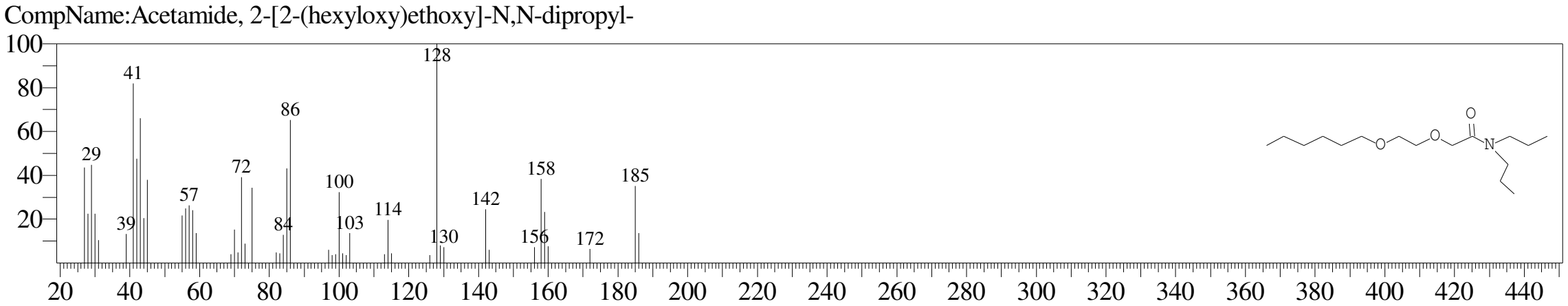

<< Target >>
Line#:17   R.Time:25.290(Scan#:5059) MassPeaks:226
RawMode:Averaged 25.285-25.295(5058-5060) BasePeak:70.00(79)
BG Mode:Calc. from Peak Group 1 - Event 1 Scan

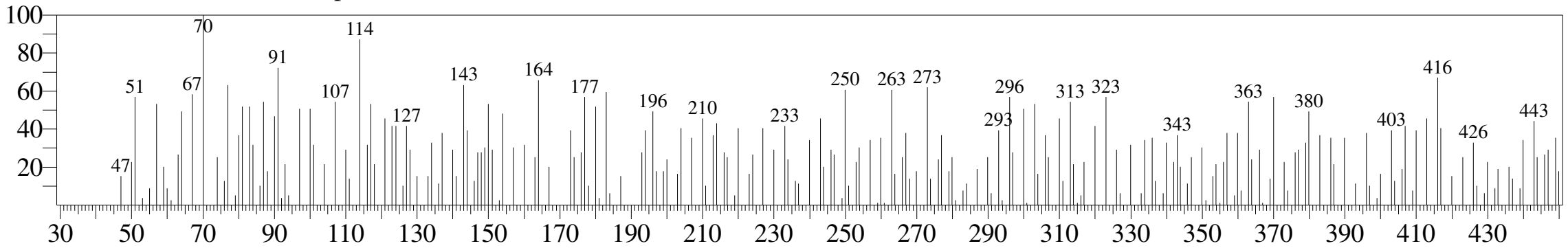

Hit#:1 Entry:58140 Library:NIST14.lib
SI:30 Formula:C13H20O CAS:55123-72-3 MolWeight:192 RetIndex:1491
CompName:2(3H)-Naphthalenone, 4,4a,5,6,7,8-hexahydro-4a,7,7-trimethyl-, (R)-

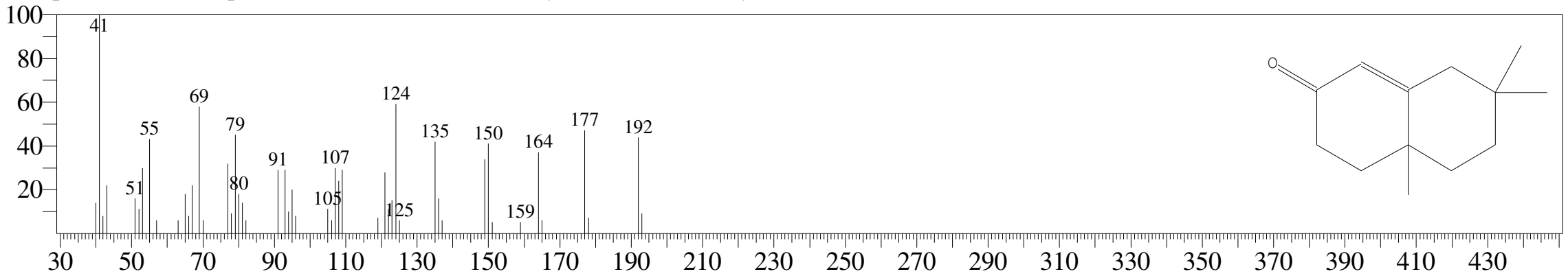

Hit#:2 Entry:144542 Library:NIST14.lib
SI:30 Formula:C20H28O CAS:116-31-4 MolWeight:284 RetIndex:2184
CompName:Retinal

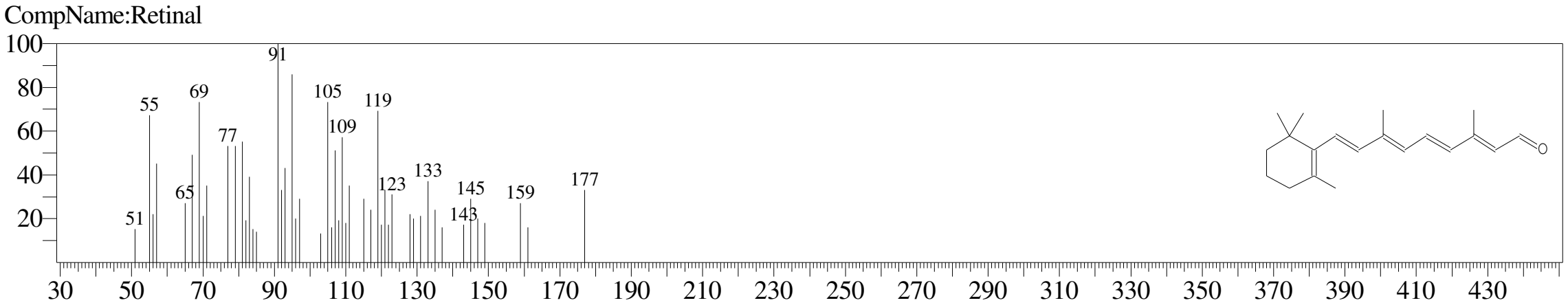

Hit#:3 Entry:74333 Library:NIST14.lib
SI:29 Formula:C11H14O4 CAS:34874-80-1 MolWeight:210 RetIndex:1899
CompName:Benzoic acid, 2-hydroxy-4-methoxy-3,5,6-trimethyl-

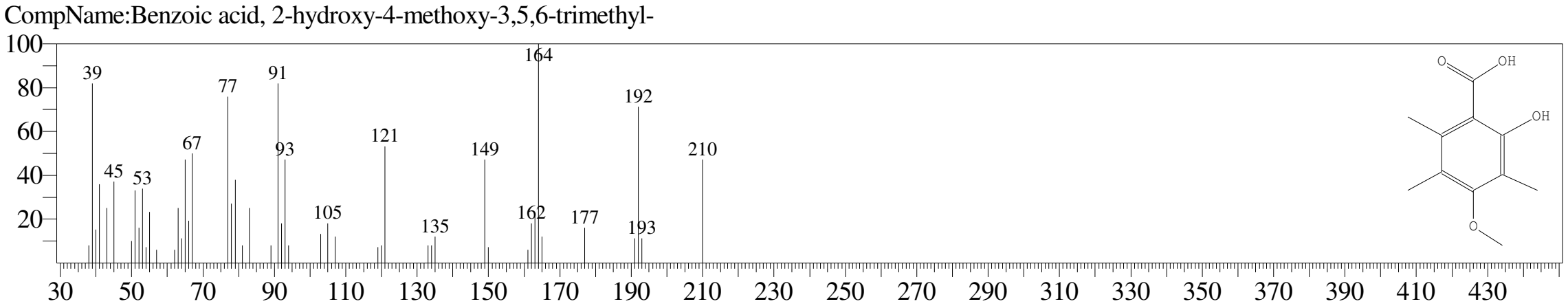

<< Target >>
Line#:18 R.Time:25.405(Scan#:5082) MassPeaks:217
RawMode:Averaged 25.400-25.410(5081-5083) BasePeak:56.00(74)
BG Mode:Calc. from Peak Group 1 - Event 1 Scan

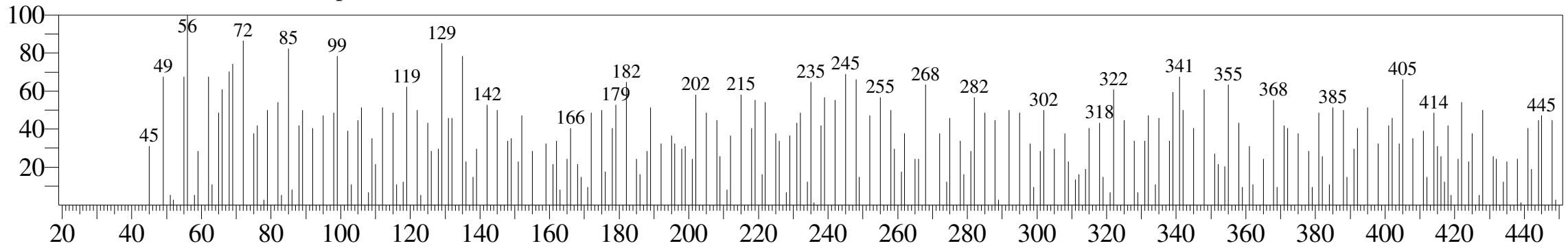

Hit#:1 Entry:12850 Library:NIST14.lib
SI:28 Formula:C8H16O CAS:28180-70-3 MolWeight:128 RetIndex:869
CompName:trans-2,3-Epoxyoctane

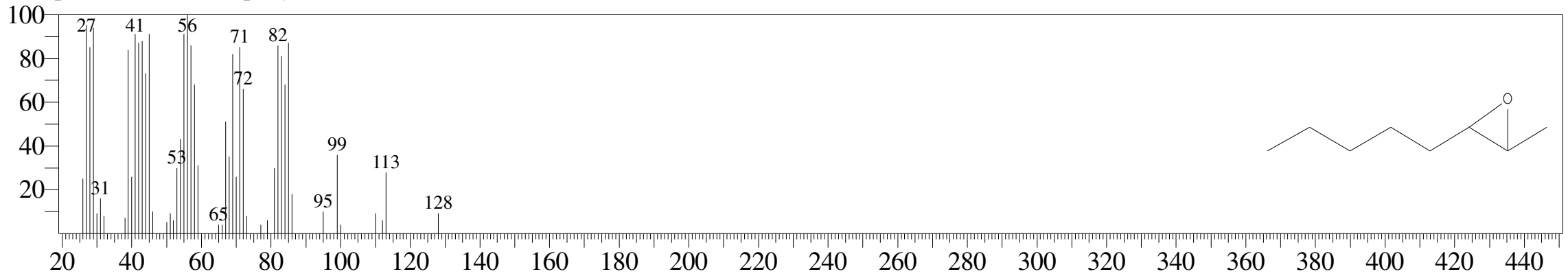

Hit#:2 Entry:29892 Library:NIST14.lib
SI:27 Formula:C10H20O CAS:54125-39-2 MolWeight:156 RetIndex:1067
CompName:trans-2,3-Epoxydecane

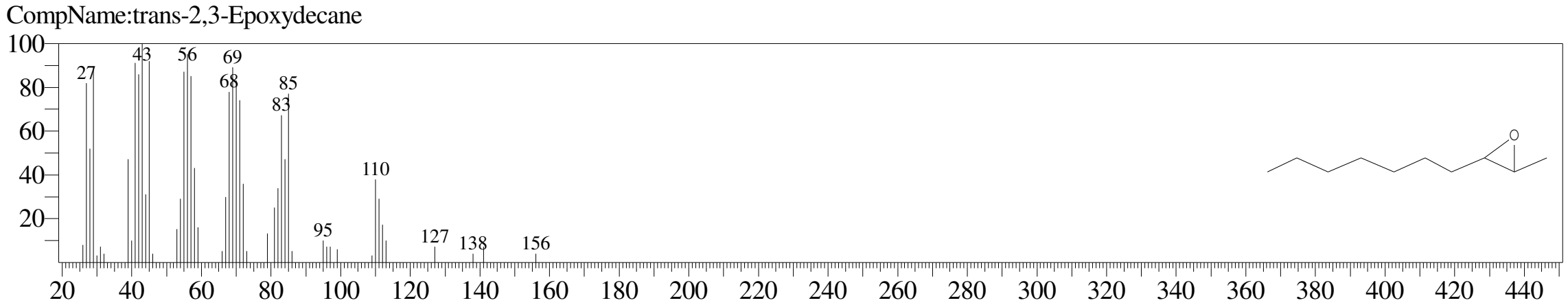

Hit#:3 Entry:12851 Library:NIST14.lib
SI:27 Formula:C8H16O CAS:23024-54-6 MolWeight:128 RetIndex:869
CompName:cis-2,3-Epoxyoctane

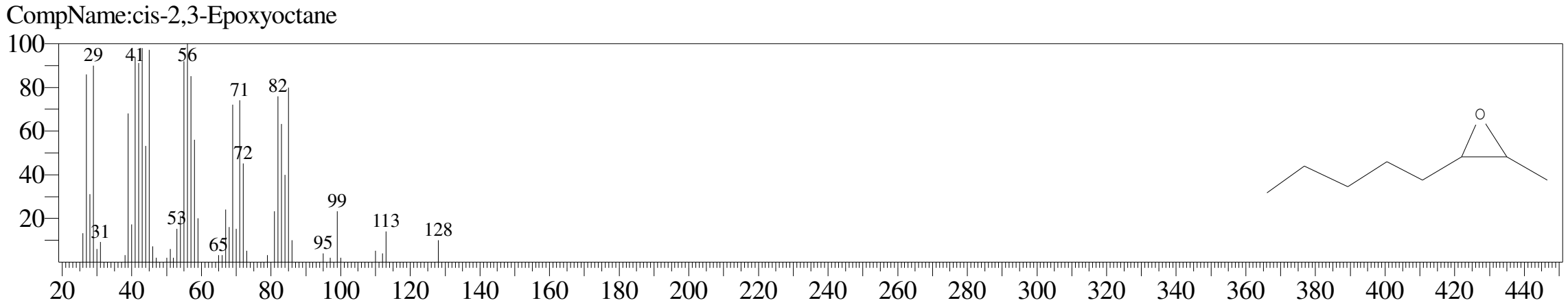

<< Target >>
Line#:19 R.Time:25.540(Scan#:5109) MassPeaks:218
RawMode:Averaged 25.535-25.545(5108-5110) BasePeak:56.00(69)
BG Mode:Calc. from Peak Group 1 - Event 1 Scan

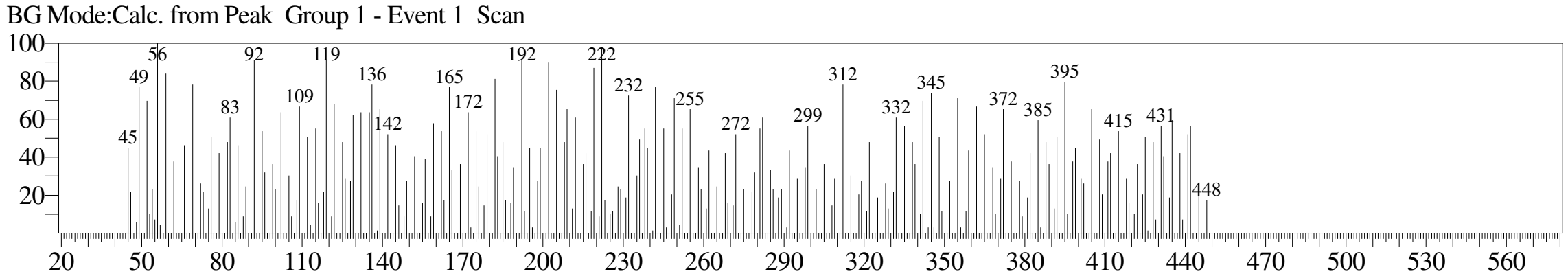

Hit#:1 Entry:265034 Library:NIST14.lib
SI:25 Formula:C27H38O8 CAS:77573-38-7 MolWeight:490 RetIndex:3169
CompName:Cyclopropa[3',4']benz[1',2':4,5]azuleno[1,8a-d]-1,3-dioxole-5b,7,7a-triol, 3a,5a,6,7,8,8a,8b,11-octahydro-10-(hydroxymethyl)-2,2,4,6,8,8-hexa

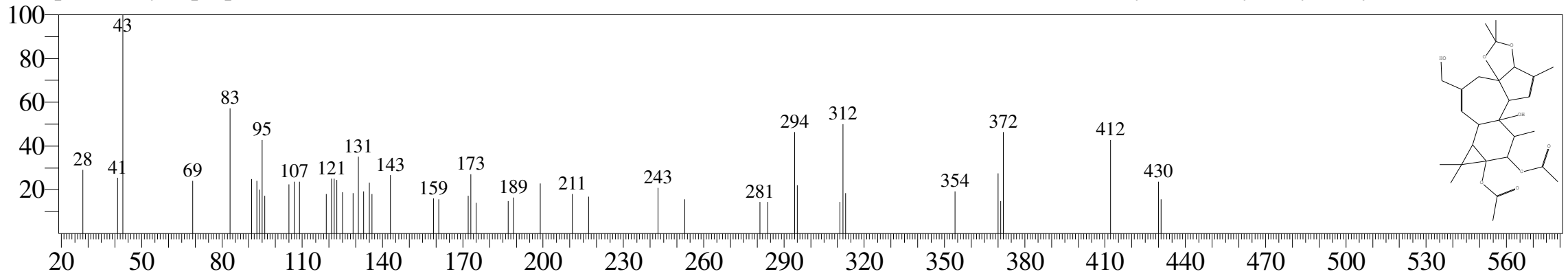

Hit#:2 Entry:272856 Library:NIST14.lib
SI:24 Formula:C12H3ClF20O2 CAS:300403-21-8 MolWeight:594 RetIndex:152
CompName:11-Chloro-2,2,3,3,4,4,5,5,6,6,7,7,8,8,9,9,10,10,11,11-eicosafluoroundecanoic acid methyl ester

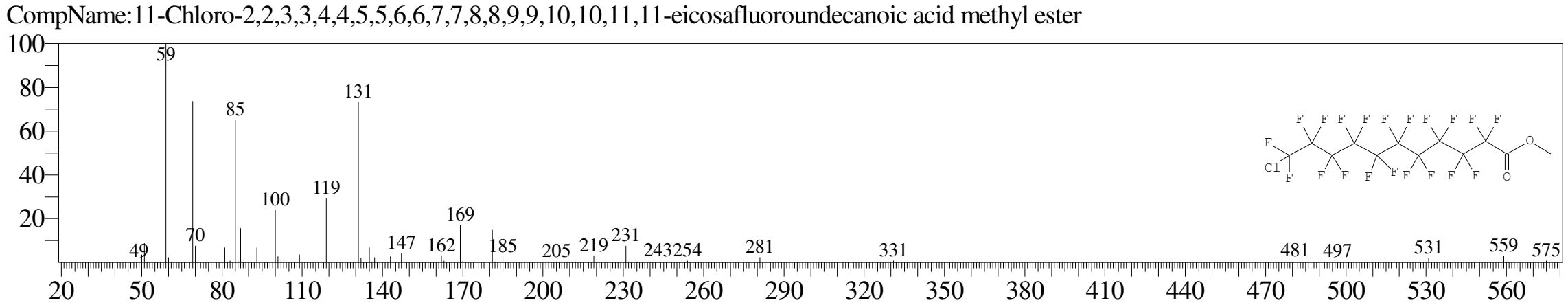

Hit#:3 Entry:143773 Library:NIST14.lib
SI:24 Formula:C15H24O5 CAS:0-00-0 MolWeight:284 RetIndex:1747
CompName:Methyl 2-[2,4a-epidioxy-3,4,5,6,7,8-hexahydro-5,5,8a-trimethyl-2H-

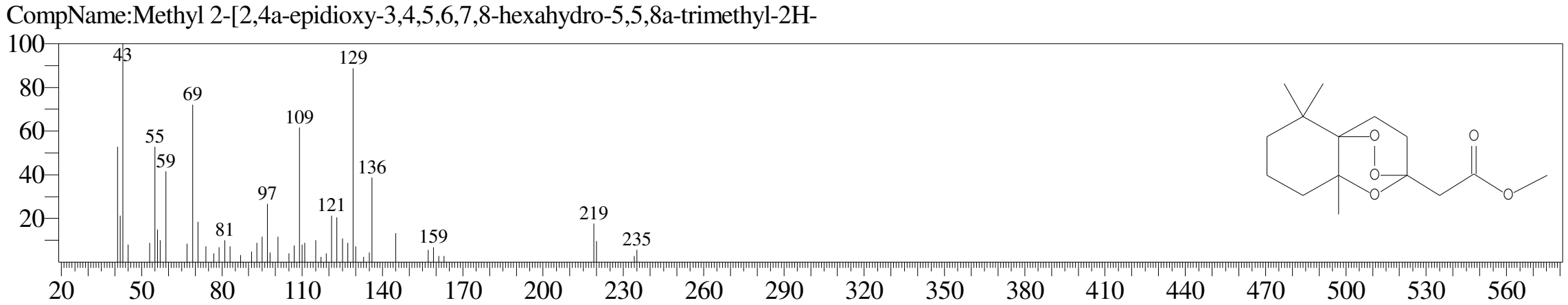

<< Target >>
Line#:20   R.Time:25.645(Scan#:5130) MassPeaks:265
RawMode:Averaged 25.640-25.650(5129-5131) BasePeak:248.00(53)
BG Mode:Calc. from Peak Group 1 - Event 1 Scan

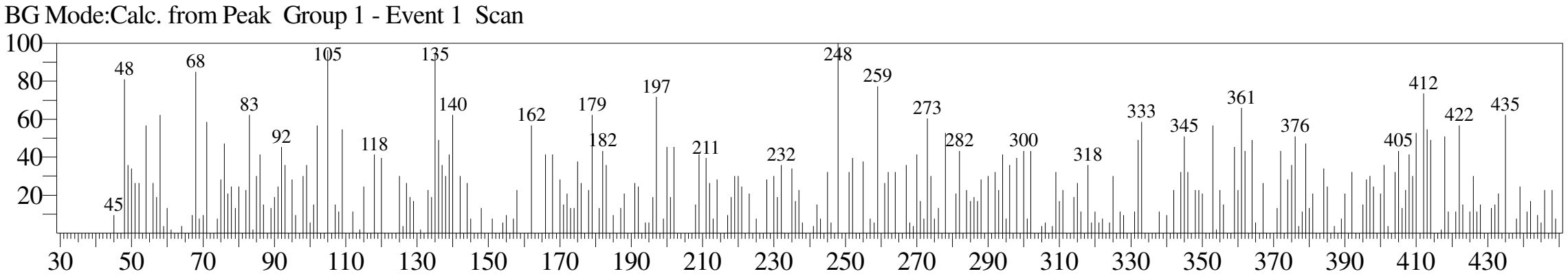

Hit#:1 Entry:88181 Library:NIST14.lib
SI:24 Formula:C7H11N7S CAS:4658-28-0 MolWeight:225 RetIndex:0
CompName:Aziprotryne

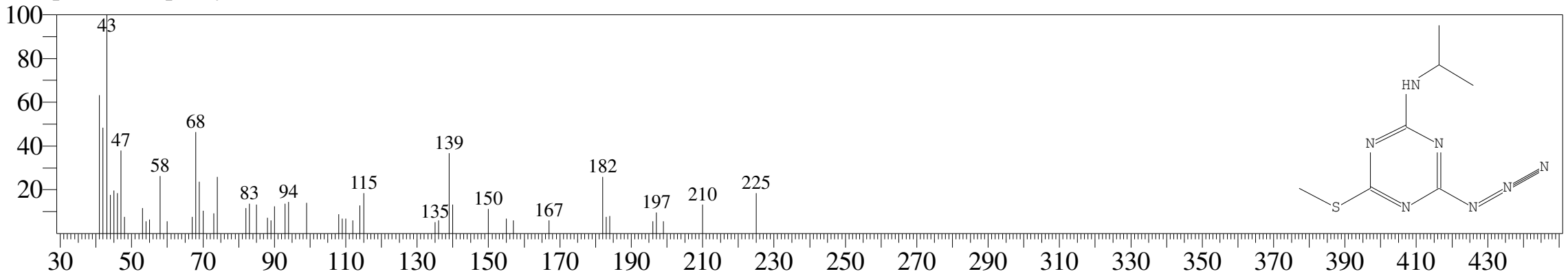

Hit#:2 Entry:190655 Library:NIST14.lib
SI:23 Formula:C15H9F2N3O2S CAS:0-00-0 MolWeight:333 RetIndex:0
CompName:4-(2H-1,3-Benzodioxol-5-yl)-5-(2,3-difluorophenyl)-1,2,4-triazole-3-thiol

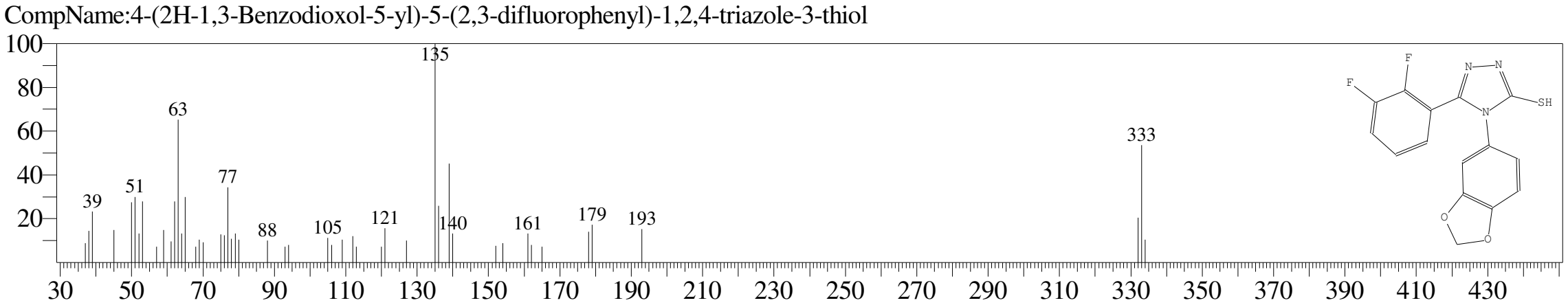

Hit#:3 Entry:190656 Library:NIST14.lib
SI:23 Formula:C15H9F2N3O2S CAS:0-00-0 MolWeight:333 RetIndex:0
CompName:4-(2H-1,3-Benzodioxol-5-yl)-5-(2,4-difluorophenyl)-1,2,4-triazole-3-thiol

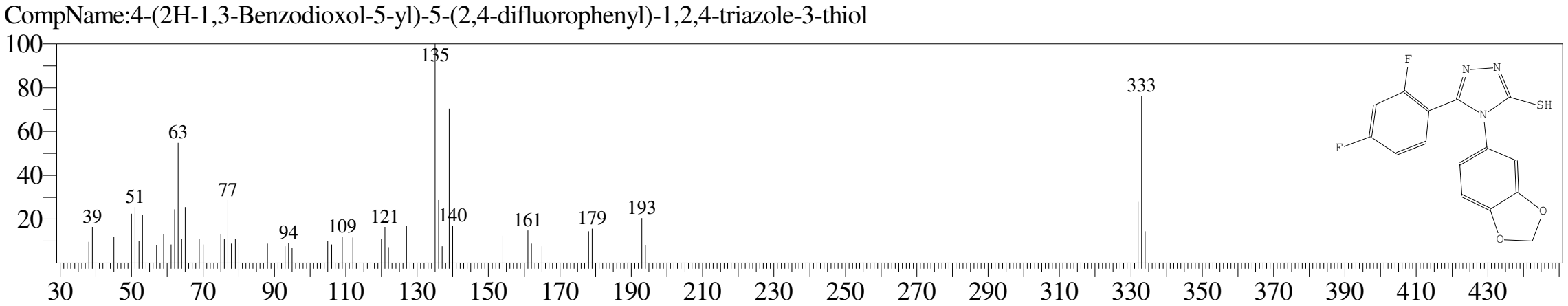

<< Target >>
Line#:21 R.Time:27.930(Scan#:5587) MassPeaks:265
RawMode:Averaged 27.925-27.935(5586-5588) BasePeak:354.90(69)
BG Mode:Calc. from Peak Group 1 - Event 1 Scan

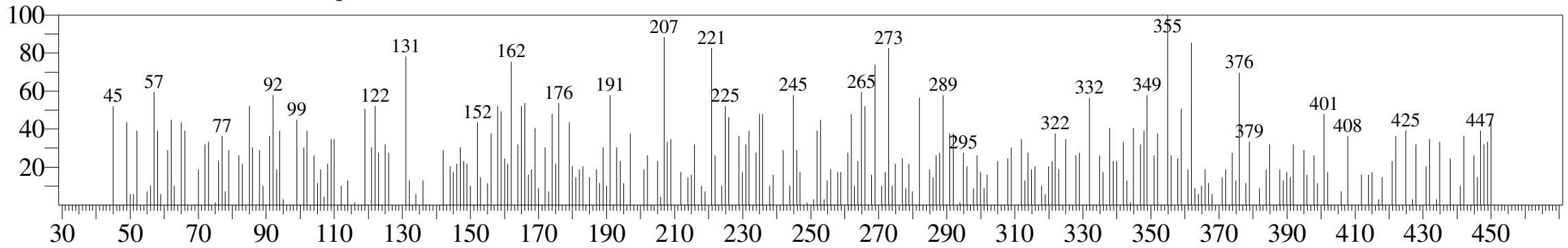

Hit#:1 Entry:272418 Library:NIST14.lib
SI:25 Formula:C24H54Sn2 CAS:813-19-4 MolWeight:582 RetIndex:0
CompName:Bis(tributyltin)

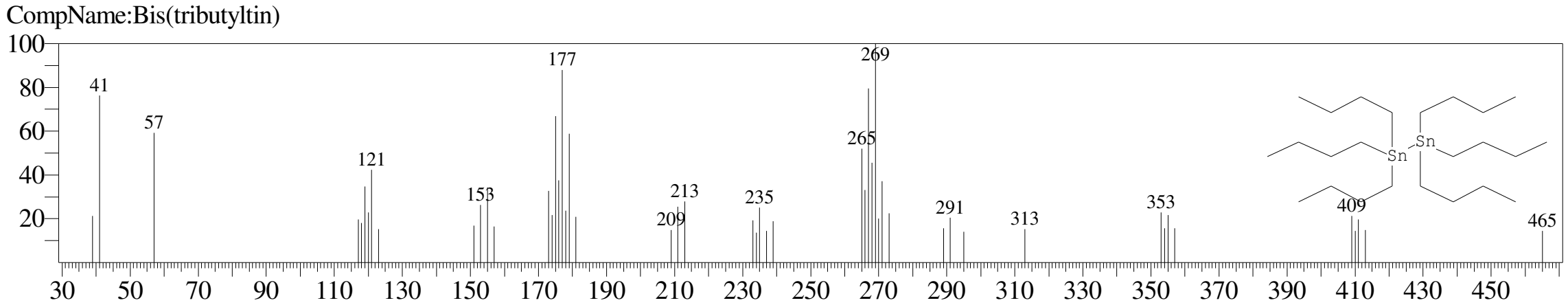

Hit#:2 Entry:185860 Library:NIST14.lib
SI:25 Formula:C17H16N2O3S CAS:0-00-0 MolWeight:328 RetIndex:2657
CompName:(3-Cyano-4-methoxymethyl-6-methyl-pyridin-2-ylsulfanyl)-acetic acid phenyl ester

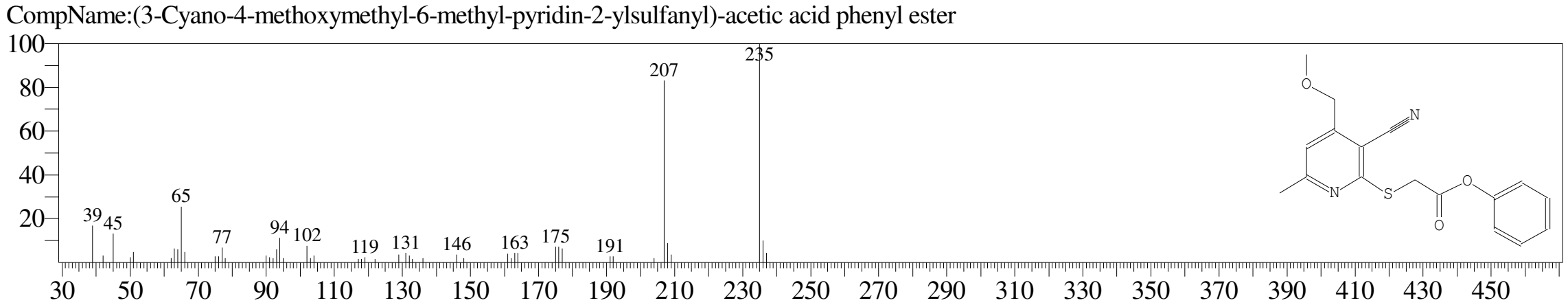

Hit#:3 Entry:189693 Library:NIST14.lib
SI:24 Formula:C16H13ClN2O4 CAS:0-00-0 MolWeight:332 RetIndex:0
CompName:Methyl 3-acetyl-6-amino-4-(3-chlorophenyl)-5-cyano-4H-pyran-2-carboxylate

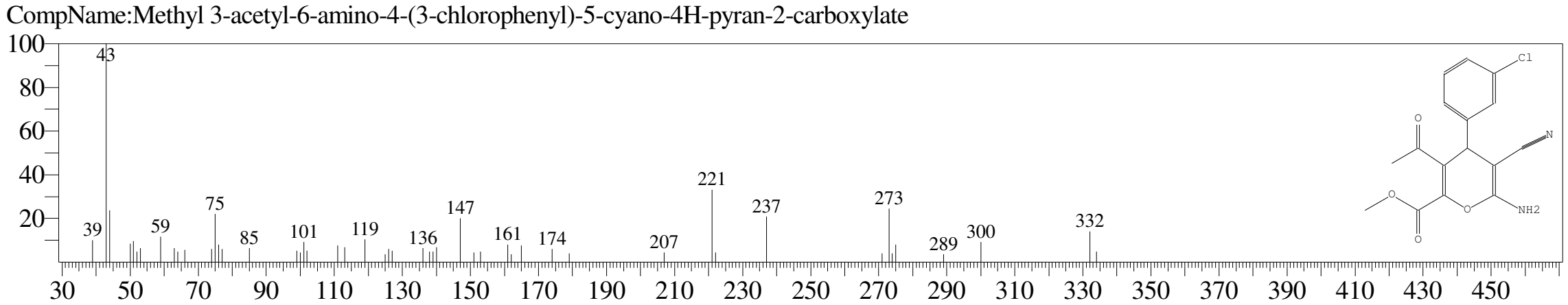

<< Target >>
Line#:22 R.Time:30.245(Scan#:6050) MassPeaks:243
RawMode:Averaged 30.240-30.250(6049-6051) BasePeak:206.95(79)
BG Mode:Calc. from Peak Group 1 - Event 1 Scan

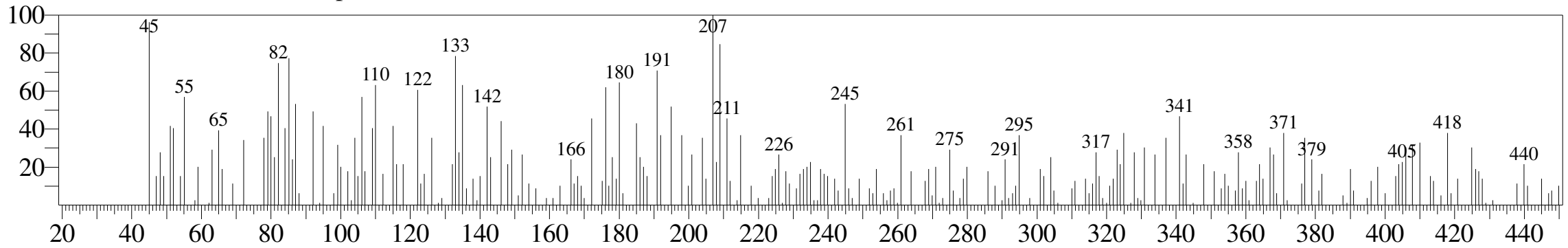

Hit#:1 Entry:98878 Library:NIST14.lib
SI:36 Formula:C16H28O CAS:0-00-0 MolWeight:236 RetIndex:1568
CompName:(-)-Isolongifolol, methyl ether

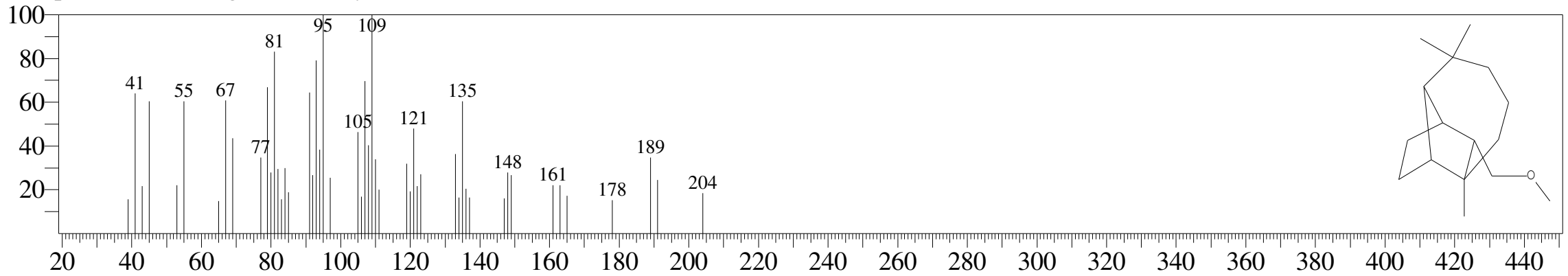

Hit#:2 Entry:204647 Library:NIST14.lib
SI:35 Formula:C13H13Cl2NO6 CAS:0-00-0 MolWeight:349 RetIndex:0
CompName:Glutaric acid, 2,2-dichloroethyl 2-nitrophenyl ester

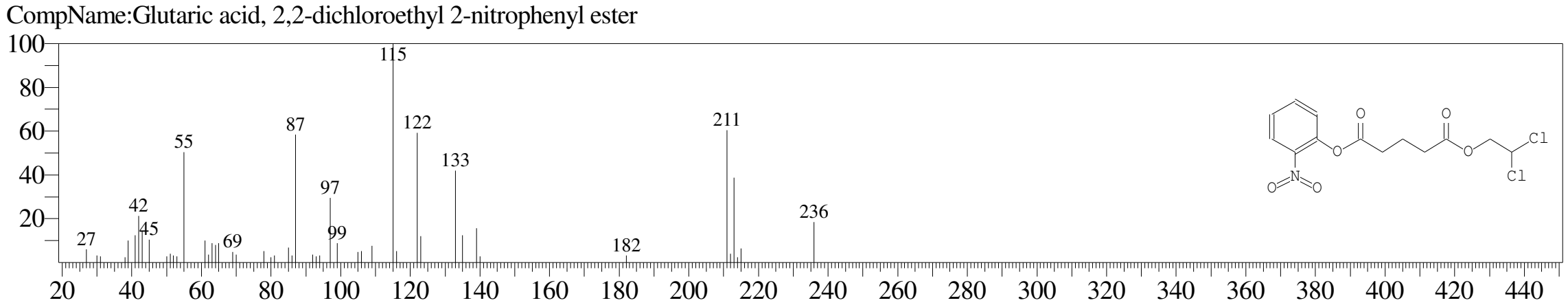

Hit#:3 Entry:71169 Library:NIST14.lib
SI:34 Formula:C5H5NO4S2 CAS:0-00-0 MolWeight:207 RetIndex:1891
CompName:4-Sulfamoyl-thiophene-2-carboxylic acid

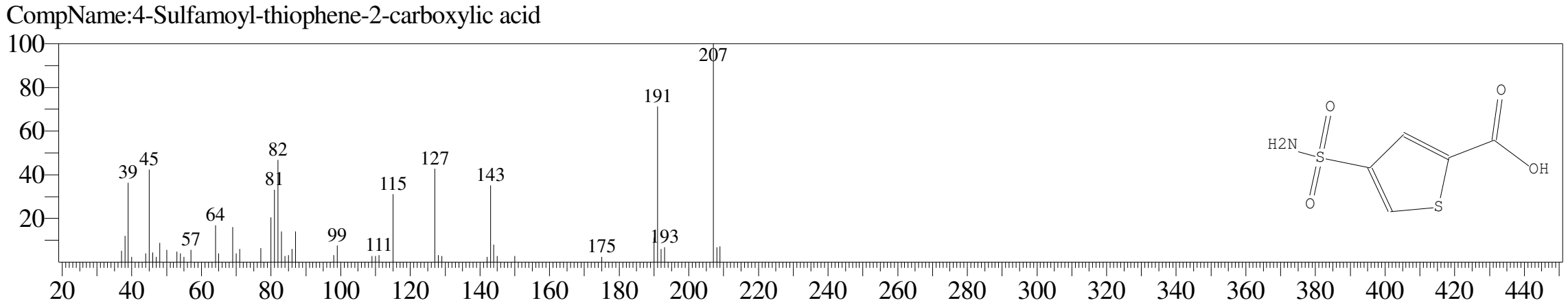

<< Target >>
Line#:23 R.Time:30.870(Scan#:6175) MassPeaks:253
RawMode:Averaged 30.865-30.875(6174-6176) BasePeak:281.00(157)
BG Mode:Calc. from Peak Group 1 - Event 1 Scan

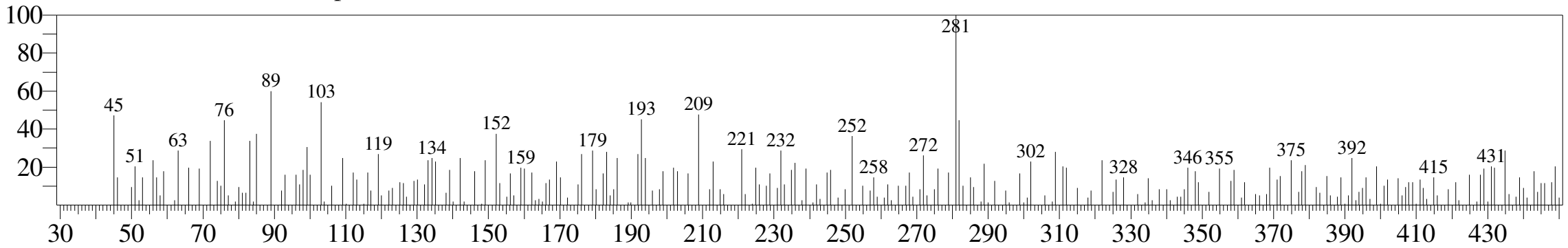

Hit#:1 Entry:100985 Library:NIST14.lib
SI:34 Formula:C10H9NO6 CAS:21021-53-4 MolWeight:239 RetIndex:2136
CompName:2-(4-Nitrophenyl)succinic acid

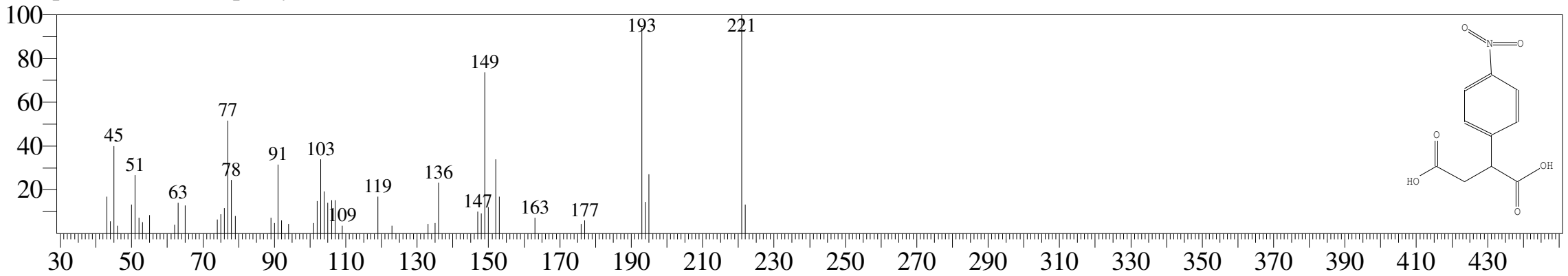

Hit#:2 Entry:140833 Library:NIST14.lib
SI:33 Formula:C15H23NO4 CAS:0-00-0 MolWeight:281 RetIndex:2202
CompName:5-Isobutyl-2-methyl-4-morpholin-4-ylmethyl-furan-3-carboxylic acid

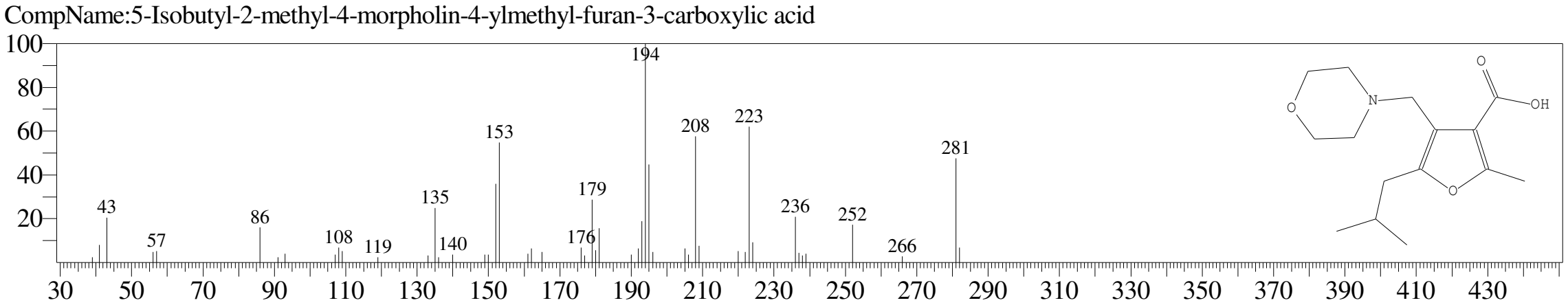

Hit#:3 Entry:155483 Library:NIST14.lib
SI:33 Formula:C17H16N2O3 CAS:351073-44-4 MolWeight:296 RetIndex:2802
CompName:N-(10,11-Dihydro-5H-dibenzo[b,f]azepin-5-yl)malonamic acid

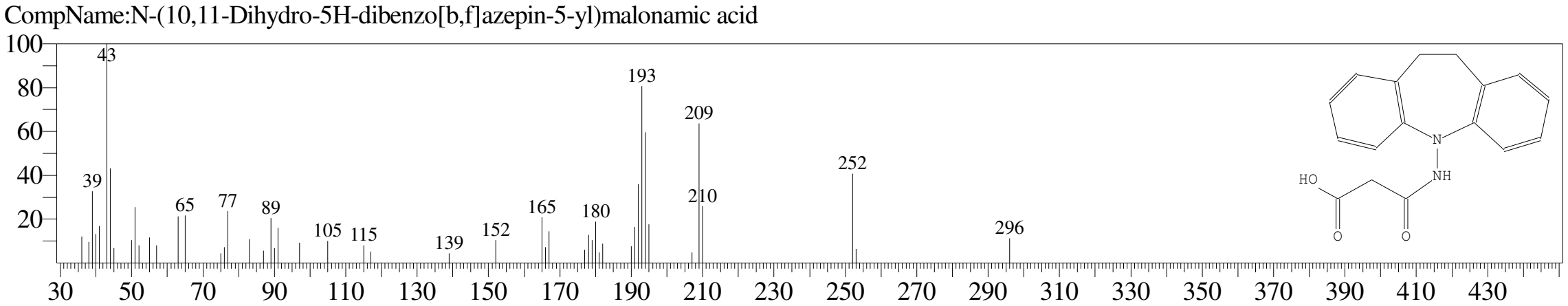

<< Target >>
Line#:24   R.Time:32.370(Scan#:6475) MassPeaks:224
RawMode:Averaged 32.365-32.375(6474-6476) BasePeak:132.95(94)
BG Mode:Calc. from Peak Group 1 - Event 1 Scan

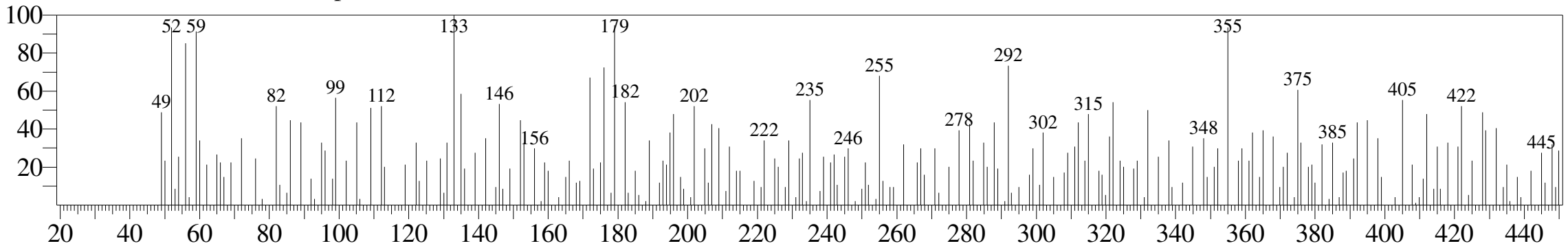

Hit#:1 Entry:255885 Library:NIST14.lib
SI:24 Formula:C29H50O3 CAS:40823-16-3 MolWeight:446 RetIndex:2914
CompName:Cholestane-3,7-diol, 3-acetate, (3.beta.,5.alpha.,7.beta.)-

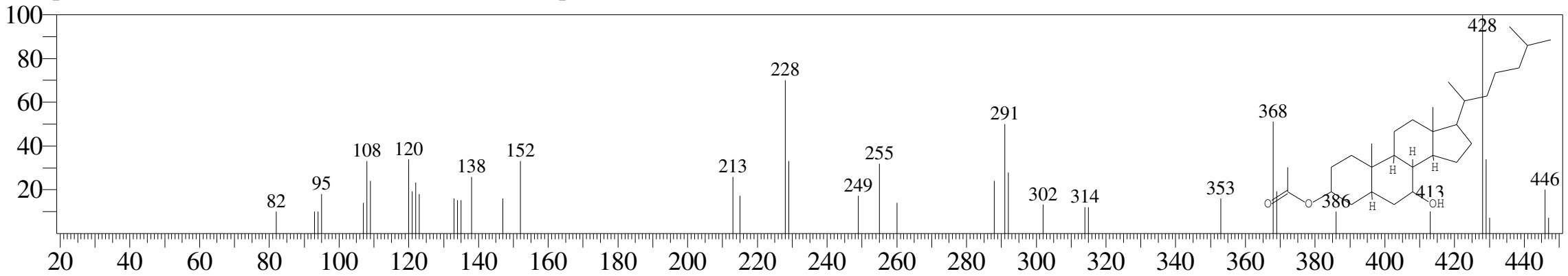

Hit#:2 Entry:124610 Library:NIST14.lib
SI:23 Formula:C14H20N2O3 CAS:0-00-0 MolWeight:264 RetIndex:2219
CompName:Benzoic acid, 2-hydroxy-5-(4-methyl-1-piperazinyl)methyl-, methyl ester

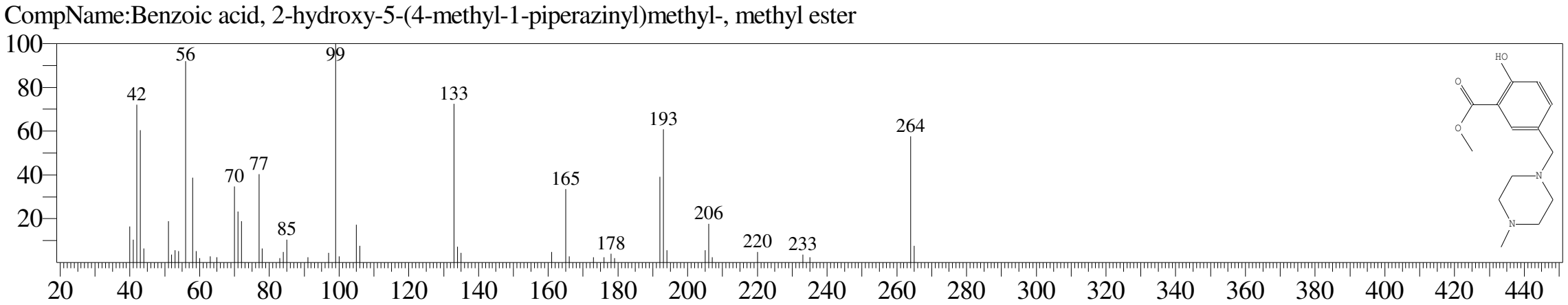

Hit#:3 Entry:89389 Library:NIST14.lib
SI:23 Formula:C12H18O4 CAS:0-00-0 MolWeight:226 RetIndex:0
CompName:benzene, 1,2-bis(dimethoxymethyl)-

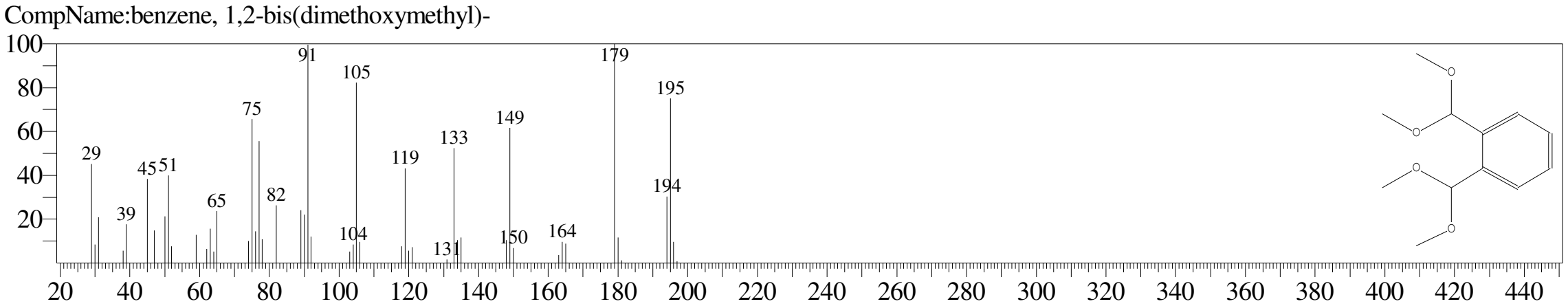

<< Target >>
Line#:25 R.Time:32.475(Scan#:6496) MassPeaks:210
RawMode:Averaged 32.470-32.480(6495-6497) BasePeak:280.95(157)
BG Mode:Calc. from Peak Group 1 - Event 1 Scan

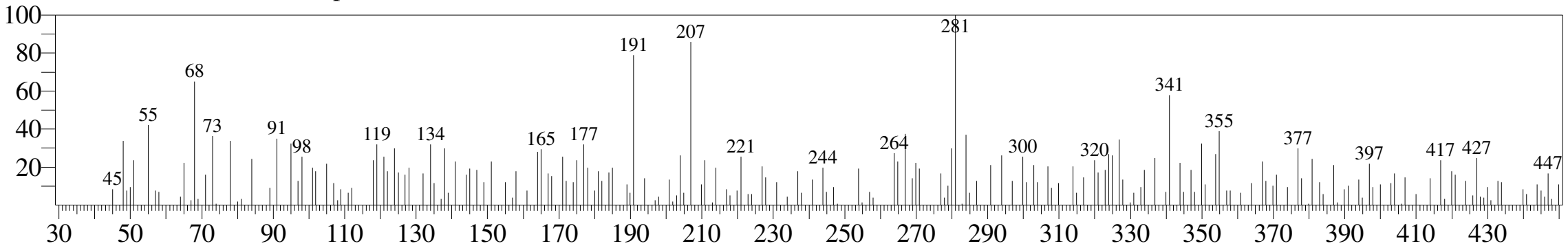

Hit#:1 Entry:139685 Library:NIST14.lib
SI:32 Formula:C14H20O4Si CAS:27750-71-6 MolWeight:280 RetIndex:1754
CompName:3,4-Dimethoxycinnamic acid, TMS derivative

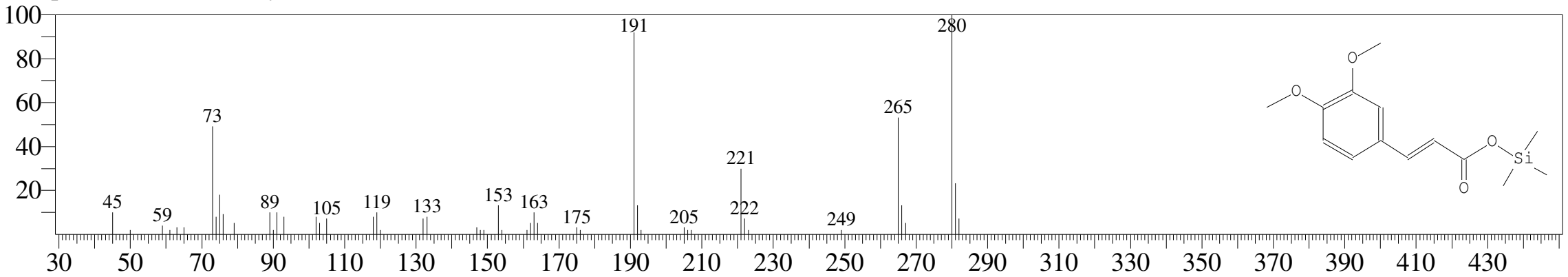

Hit#:2 Entry:198319 Library:NIST14.lib
SI:32 Formula:C22H19N3O CAS:16983-75-8 MolWeight:341 RetIndex:3224
CompName:Acrylophenone, 3,3-diphenyl-, semicarbazone

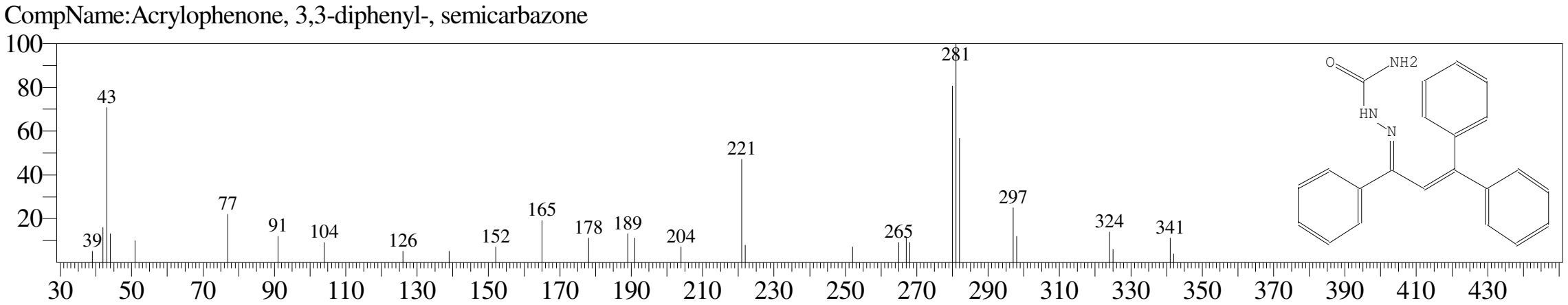

Hit#:3 Entry:276066 Library:NIST14.lib
SI:31 Formula:C26H74O11Si12 CAS:118229-41-7 MolWeight:898 RetIndex:2450
CompName:3,3,5,5,7,7,9,9,11,11,13,13-Dodecamethyl-1,15-bis(1,3,3,5,5-pentamethyl-2,4,6-trioxa-1,3,5-trisilacyclohexyl0-4,6,8,10,12-tetraoxa-3,5,7,9,11-

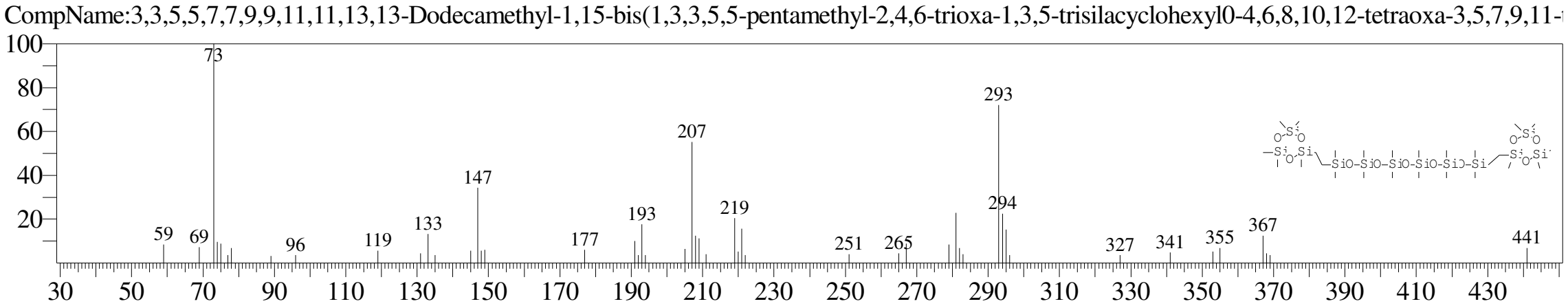

<< Target >>
Line#:26 R.Time:32.565(Scan#:6514) MassPeaks:251
RawMode:Averaged 32.560-32.570(6513-6515) BasePeak:281.00(108)
BG Mode:Calc. from Peak Group 1 - Event 1 Scan

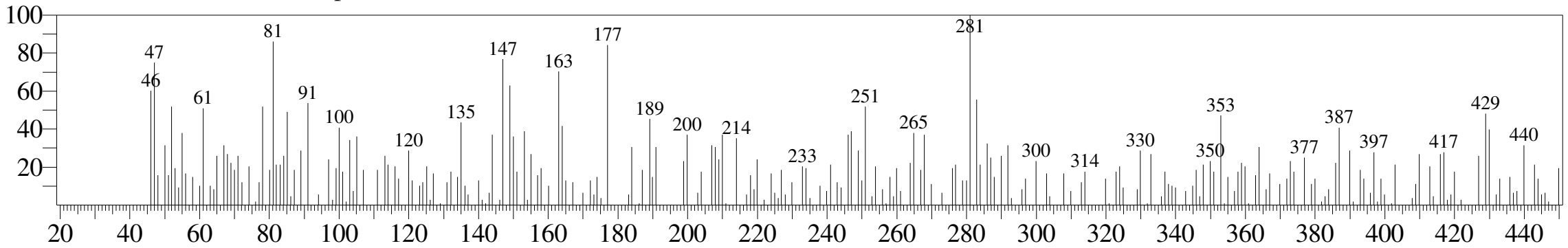

Hit#:1 Entry:74333 Library:NIST14.lib
SI:33 Formula:C11H14O4 CAS:34874-80-1 MolWeight:210 RetIndex:1899
CompName:Benzoic acid, 2-hydroxy-4-methoxy-3,5,6-trimethyl-

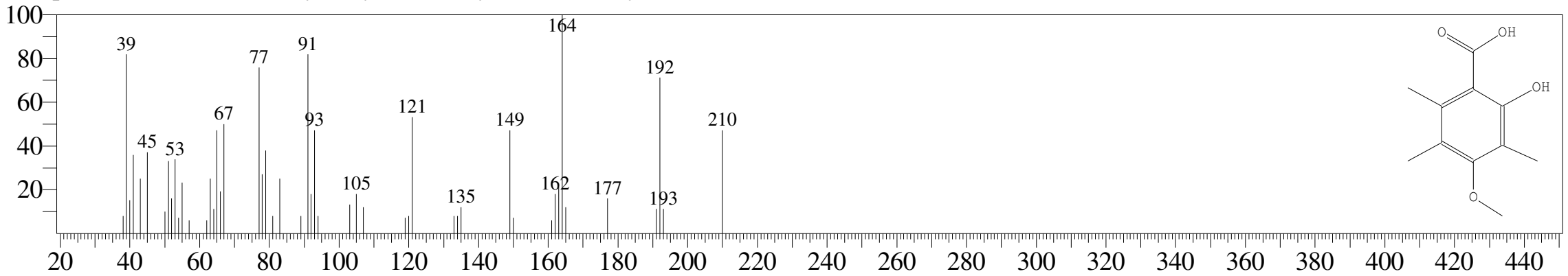

Hit#:2 Entry:100655 Library:NIST14.lib
SI:32 Formula:C15H26O2 CAS:62600-05-9 MolWeight:238 RetIndex:1786
CompName:Cedran-diol, (8S,14)-

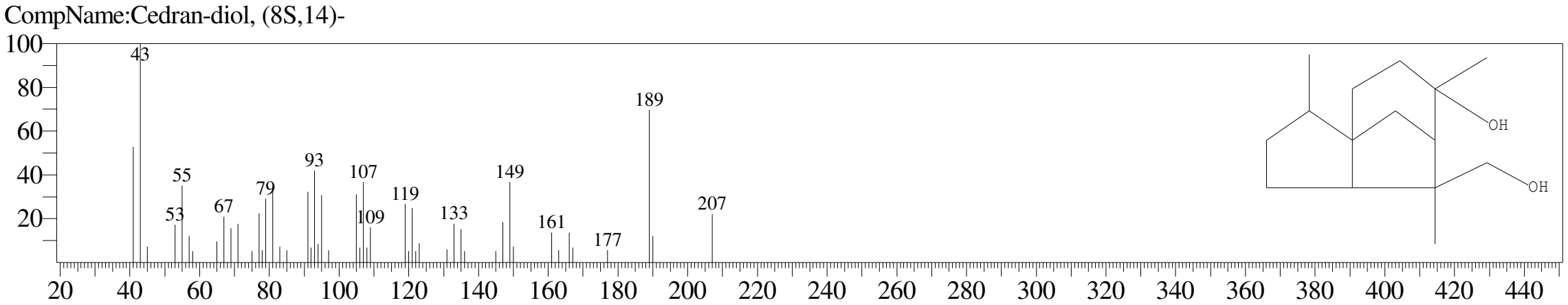

Hit#:3 Entry:124407 Library:NIST14.lib
SI:31 Formula:C13H12O4S CAS:0-00-0 MolWeight:264 RetIndex:0
CompName:Ethyl 2-[(2-oxochromen-4-yl)sulfanyl]acetate

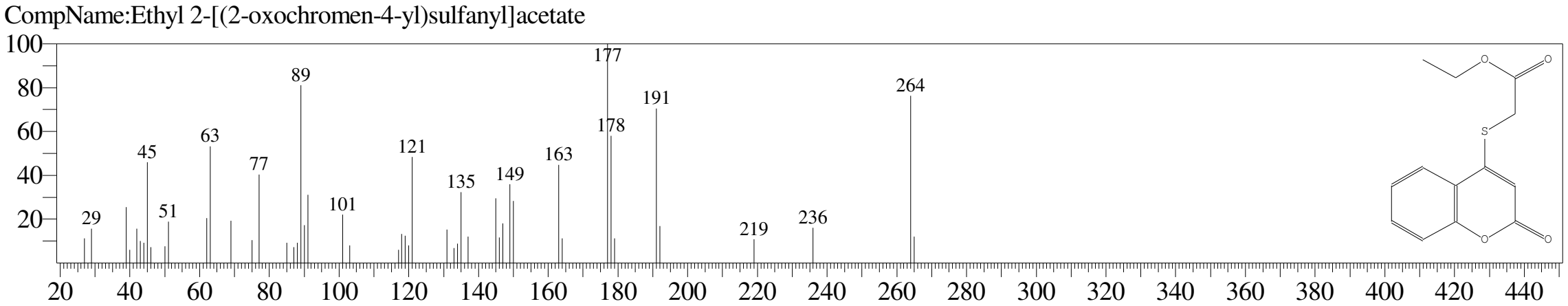

<< Target >>
Line#:27   R.Time:32.720(Scan#:6545) MassPeaks:243
RawMode:Averaged 32.715-32.725(6544-6546) BasePeak:280.95(211)
BG Mode:Calc. from Peak Group 1 - Event 1 Scan

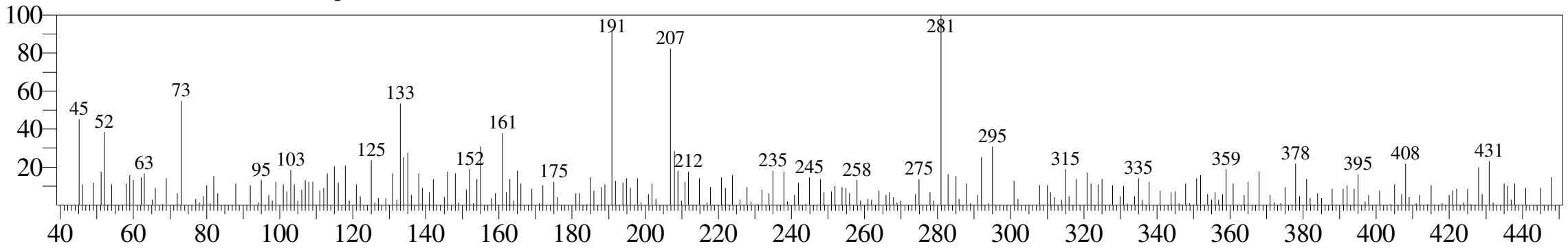

Hit#:1 Entry:155085 Library:NIST14.lib
SI:44 Formula:C14H24O3Si2 CAS:0-00-0 MolWeight:296 RetIndex:1580
CompName:Trimethylsilyl 3-methyl-4-[(trimethylsilyl)oxy]benzoate

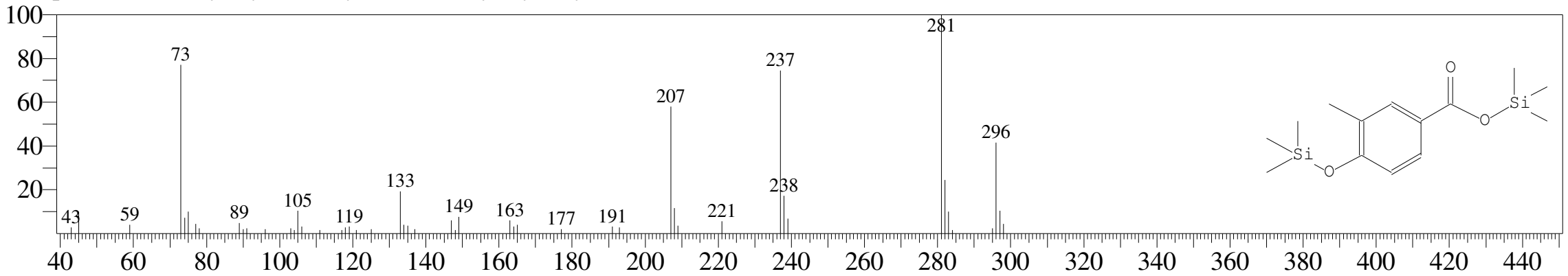

Hit#:2 Entry:139314 Library:NIST14.lib
SI:43 Formula:C9H28O2Si4 CAS:139347-50-5 MolWeight:280 RetIndex:851
CompName:Trimethylsilyl-di(timethylsiloxy)-silane

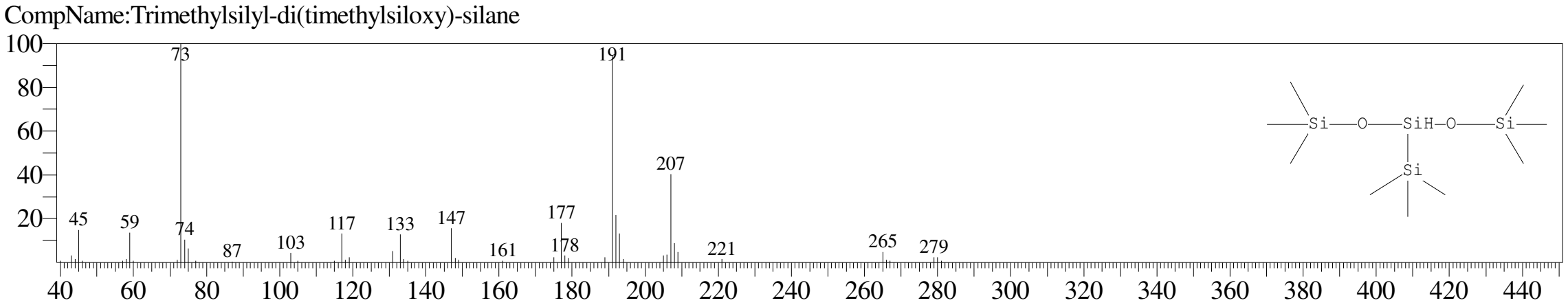

Hit#:3 Entry:168344 Library:NIST14.lib
SI:42 Formula:C10H30O3Si4 CAS:17928-28-8 MolWeight:310 RetIndex:883
CompName:Methyltris(trimethylsiloxy)silane

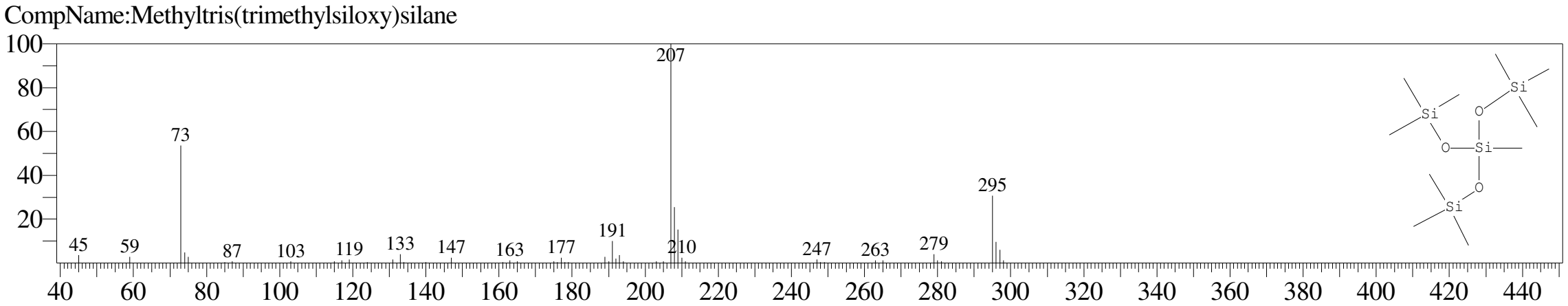

<< Target >>
Line#:28 R.Time:34.785(Scan#:6958) MassPeaks:223
RawMode:Averaged 34.780-34.790(6957-6959) BasePeak:45.00(100)
BG Mode:Calc. from Peak Group 1 - Event 1 Scan

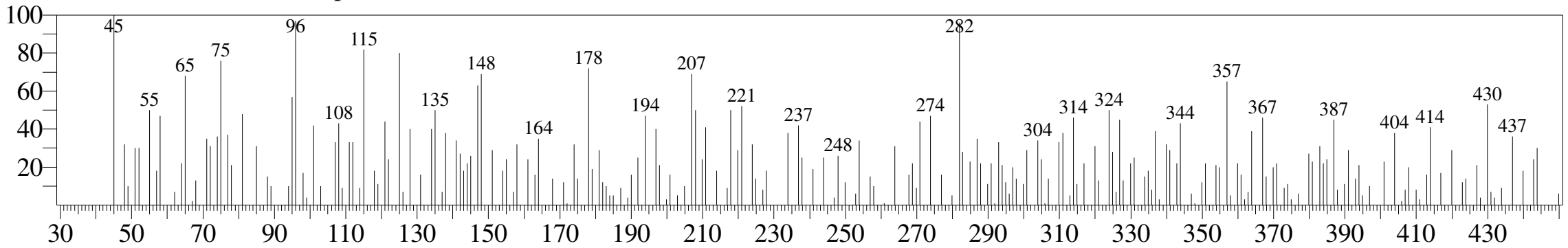

Hit#:1 Entry:63668 Library:NIST14.lib
SI:33 Formula:C12H22O2 CAS:41766-63-6 MolWeight:198 RetIndex:1375
CompName:Naphthalene, decahydro-1,4-dimethoxy-, (1.alpha.,4.alpha.,4a.alpha.,8a.beta.)-

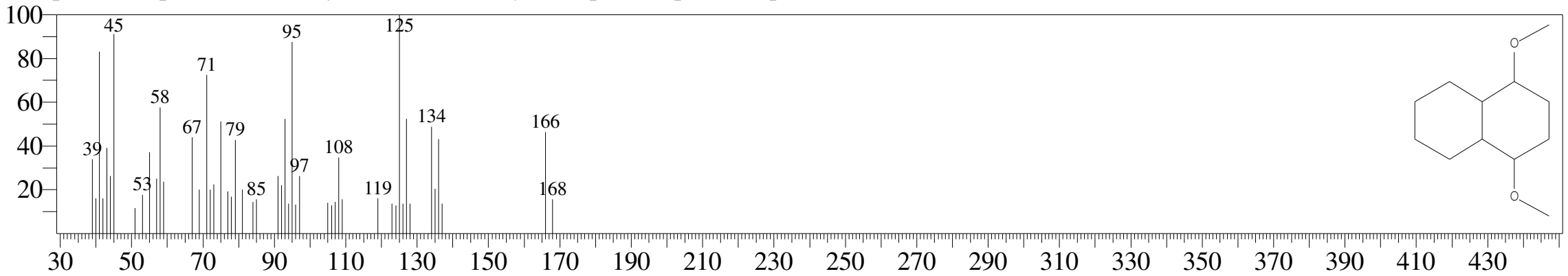

Hit#:2 Entry:63666 Library:NIST14.lib
SI:31 Formula:C12H22O2 CAS:41766-64-7 MolWeight:198 RetIndex:1375
CompName:Naphthalene, decahydro-1,4-dimethoxy-, (1.alpha.,4.alpha.,4a.alpha.,8a.alpha.)-

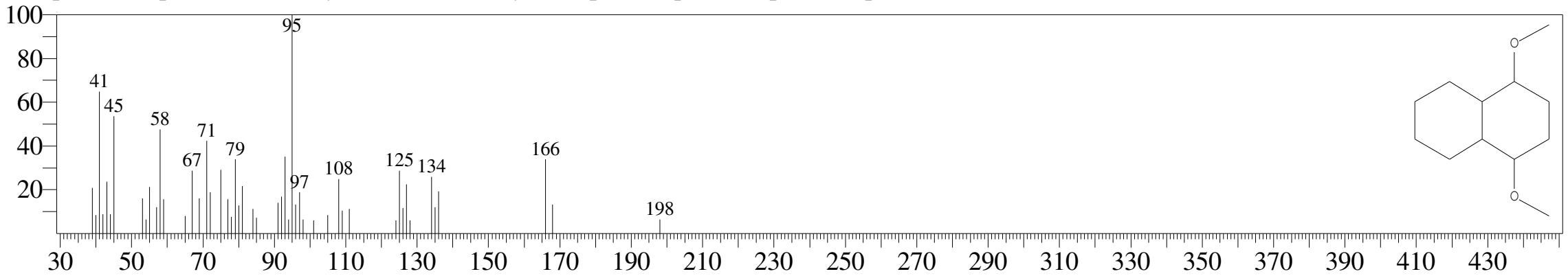

Hit#:3 Entry:63667 Library:NIST14.lib
SI:31 Formula:C12H22O2 CAS:42067-56-1 MolWeight:198 RetIndex:1375
CompName:Naphthalene, decahydro-1,4-dimethoxy-, (1.alpha.,4.alpha.,4a.beta.,8a.beta.)-

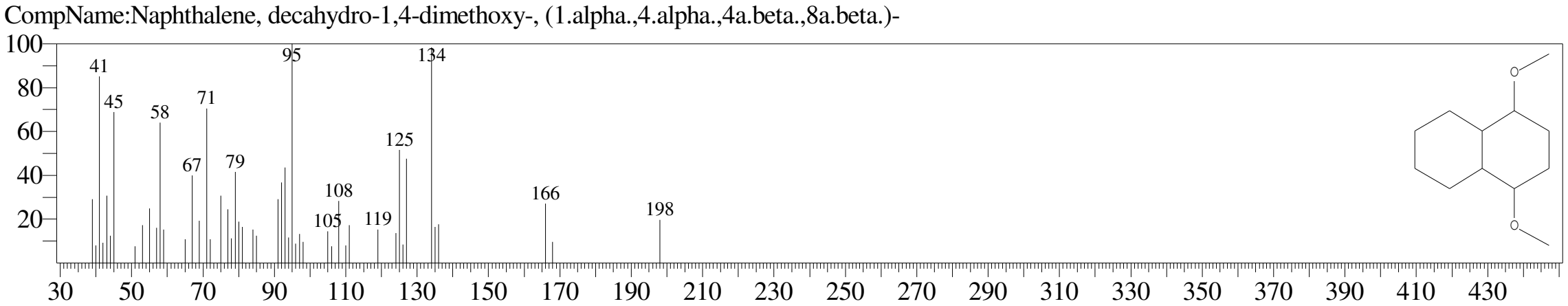

<< Target >>
Line#:29 R.Time:34.895(Scan#:6980) MassPeaks:247
RawMode:Averaged 34.890-34.900(6979-6981) BasePeak:206.90(596)
BG Mode:Calc. from Peak Group 1 - Event 1 Scan

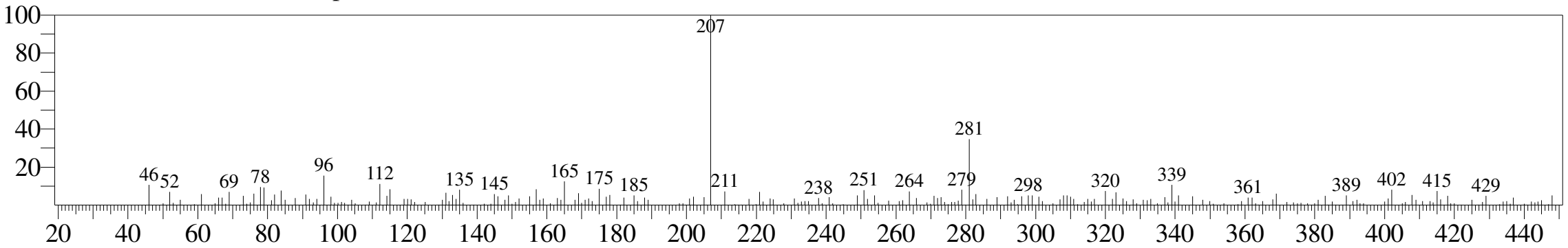

Hit#:1 Entry:124936 Library:NIST14.lib
SI:44 Formula:C16H28OSi CAS:0-00-0 MolWeight:264 RetIndex:1553
CompName:Carvacrol, TBDMS derivative

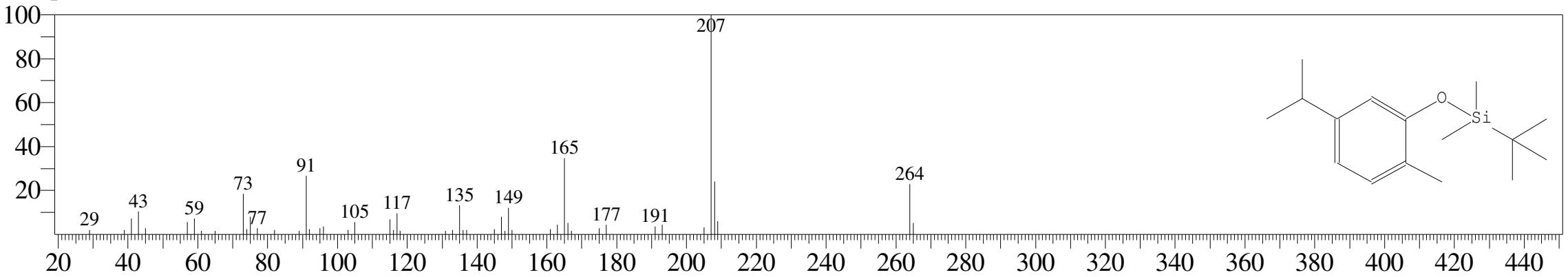

Hit#:2 Entry:166020 Library:NIST14.lib
SI:42 Formula:C15H21N3O4 CAS:89790-63-6 MolWeight:307 RetIndex:2471
CompName:4-Acetamido-N,N-diisopropyl-3-nitrobenzamide

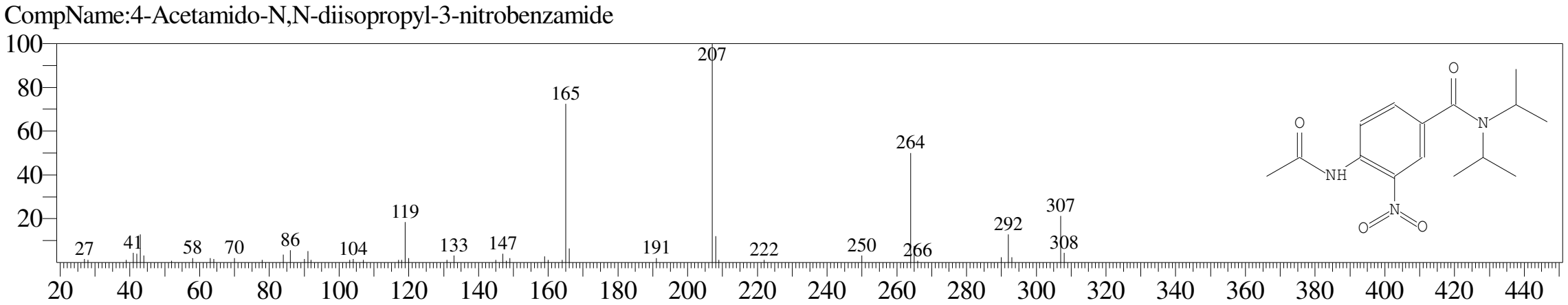

Hit#:3 Entry:84723 Library:NIST14.lib
SI:42 Formula:C6H18O3Si3 CAS:541-05-9 MolWeight:222 RetIndex:620
CompName:Cyclotrisiloxane, hexamethyl-

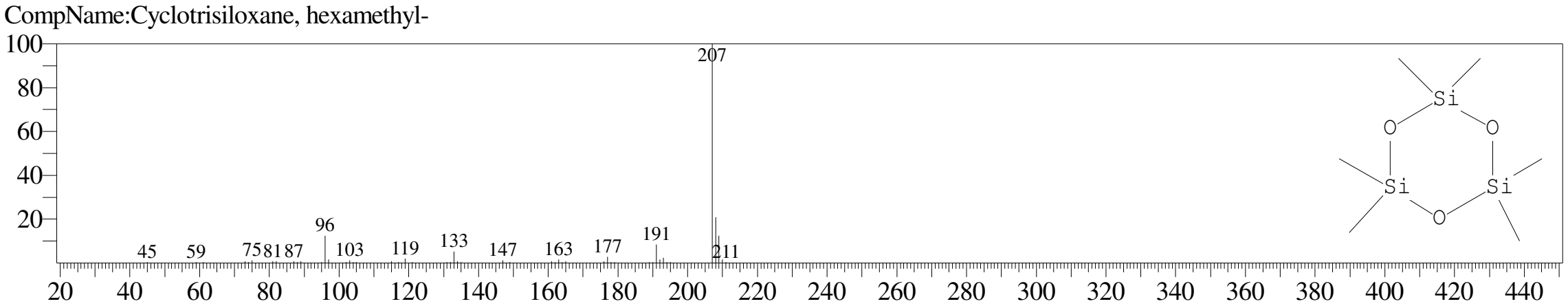

<< Target >>
Line#:30 R.Time:35.605(Scan#:7122) MassPeaks:213
RawMode:Averaged 35.600-35.610(7121-7123) BasePeak:103.00(131)
BG Mode:Calc. from Peak Group 1 - Event 1 Scan

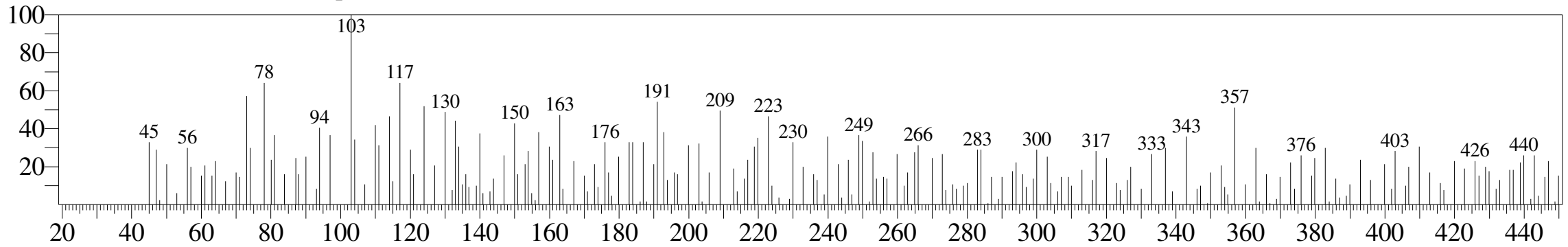

Hit#:1 Entry:126003 Library:NIST14.lib
SI:34 Formula:C11H14N4O4 CAS:26820-41-7 MolWeight:266 RetIndex:2337
CompName:2-Cyclohexylamino-3,5-dinitropyridine

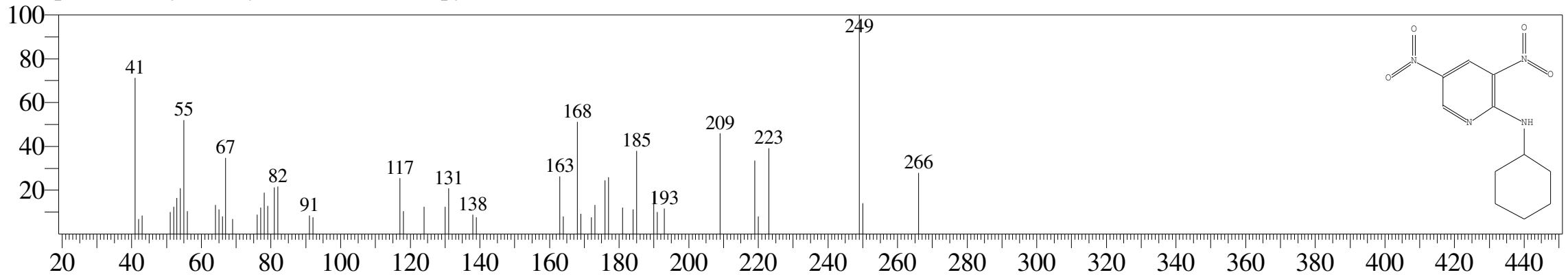

Hit#:2 Entry:193274 Library:NIST14.lib
SI:32 Formula:C14H36O3Si3 CAS:74685-21-5 MolWeight:336 RetIndex:1242
CompName:3,8-Dioxa-2,9-disiladecane, 2,2,4,9,9-pentamethyl-5-[(trimethylsilyl)oxy]-

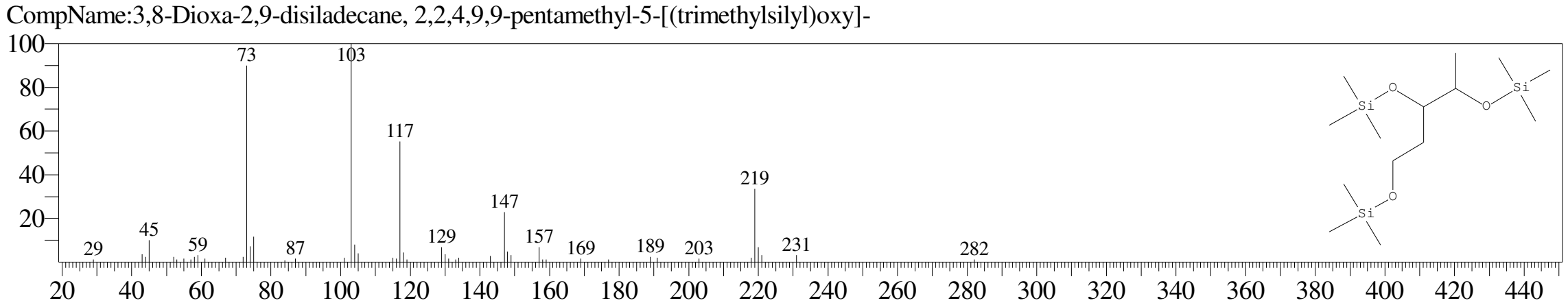

Hit#:3 Entry:241612 Library:NIST14.lib
SI:32 Formula:C19H19Cl2N3OS CAS:339352-38-4 MolWeight:407 RetIndex:3293
CompName:3-(3,4-Dichlorophenyl)-1-(5-methyl-2-thiazolin-2-yl)-1-(3,4-xylyl)urea

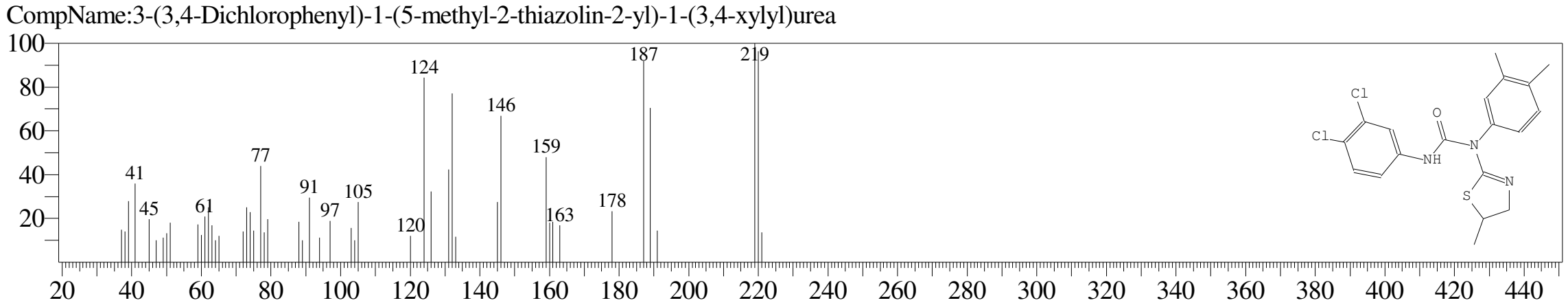

Supplement: Supplementary file 1 [file IJM-2026-4151399-s002.pdf]
